# Diabatization with Electrostatic Embedding for Studying Photophysics in Organic Molecular Crystals

**DOI:** 10.1021/acs.jctc.5c01934

**Published:** 2026-02-23

**Authors:** Michael Ingham, Mohammad Aarabi, Samuele Giannini, Marco Garavelli, Fabrizio Santoro, Roberto Improta, Rachel Crespo-Otero

**Affiliations:** † Department of Chemistry, 4919 University College London, 20 Gordon Street, WC1H 0AJ London, United Kingdom; ‡ Dipartimento di Chimica Industriale “Toso Montanari”, Universitá di Bologna - Alma Mater Studiorum, Via Piero Gobetti 85, 40129 Bologna, Italy; § Department of Chemistry and Industrial Chemistry, 9310 University of Pisa, Via Giuseppe Moruzzi, 56124 Pisa, Italy; ∥ Consiglio Nazionale delle Ricerche, 9296 Istituto di Chimica dei Composti Organo Metallici (ICCOM-CNR), I-56124 Pisa, Italy; ⊥ 201844 Istituto di Biostrutture e Bioimmagini-CNR (IBB-CNR), Via De Amicis 95, I-80145 Napoli, Italy

## Abstract

Highly emissive organic molecular crystals find applications in several areas, such as organic electronics, solar cells, and sensors. Understanding the excited-state mechanisms underlying these applications is essential for optimizing and controlling them effectively. Exciton models coupled with nonadiabatic dynamics, particularly quantum dynamics, provide crucial insights into photochemical and photophysical processes in molecular crystals. Nevertheless, there remains a lack of general tools and automated workflows to facilitate such simulations. In this paper, we present a computational strategy to investigate the photoactivated dynamics of organic molecular crystals, bridging methodologies traditionally used for molecular systems and materials science, with a particular focus on the interplay between local excitations and charge transfer (CT) processes. We have implemented an interface between the fromage and Overdia programs, enabling the construction of vibronic Hamiltonians for molecular crystals within an excited-state ONIOM­(QM:QM′) framework, incorporating long-range electrostatics through a RESP-based Ewald summation. Fragment-based diabatization provides a route to quantum dynamics simulations in weak-to-intermediate coupling regimes. The method was applied to the photophysics of dibenzo­[g,p]­chrysene (DBC) crystals using time-dependent DFT. The fromage/Overdia interface was employed to compute the couplings of local excitations and CT states for 18 unique DBC dimers in the crystal and to quantify the influence of electrostatic embedding, which was found to be modest (10–20%). Simulations on π-stacked dimers reproduced the small red shift observed experimentally from solution to crystal, attributed to electronic interactions among fixed monomers rather than crystal electrostatics. Quantum dynamics simulations revealed ultrafast population transfer from bright local excitations to CT states. This approach establishes a robust framework linking molecular and solid-state excited-state dynamics, with potential applications for studying excitations, defects, and impurities in molecular crystals.

## Graphical abstract

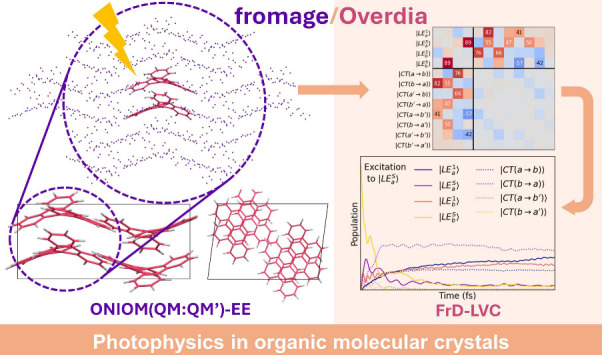

## 1 Introduction

Understanding the interaction between light and matter is crucial for harnessing a wide range of phenomena in biological and materials sciences, from photosynthesis and oxidative DNA damage to organic light-emitting diodes (OLEDs) and solar cells.^1–3^ These processes occur in the condensed phase, either in solution or in the solid state, and involve molecular aggregates composed of multiple chromophores. In such systems, electronic excitations can delocalize over spatially separated molecular units, forming excitons. Both Frenkel and charge transfer excitons are known to play significant roles in the photochemistry and photophysics of multichromophoric (MC) systems.^3–7^ A detailed understanding of excited-state mechanisms in MC assemblies can help improve control over relevant applications. However, describing realistic MC systems presents significant challenges due to the need for nonadiabatic dynamics simulations, which become computationally unfeasible as the number of species and the size of the molecules increase, including the number of excited states and vibrational modes. In this context, exciton models based on the excited states of individual molecular units have proven extremely useful for exploring excited-state dynamics with good accuracy and manageable computational costs.^8–12^

Significant efforts have been devoted to designing highly emissive organic crystals,^13,14^ which are examples of MC systems in which the molecular chromophores are held together by weak interactions, finding applications in optoelectronics and OLEDs. Understanding emission behavior requires a detailed knowledge of the excited-state potential energy surfaces (PESs), including the S_1_ minima from which emission occurs, how efficiently these states are populated following light absorption, as well as the fluorescence process itself. Indeed, excited-state dynamics in molecular crystals are governed by a competition between radiative and nonradiative processes. Depending on the strength of excitonic couplings and their interactions with vibrational modes in comparison to molecular reorganization energies, excitations may become delocalized or localized over a single molecule or a small group of molecules. The investigation of exciton dynamics in these crystals can help decipher the competition between localization and delocalization and the role of charge transfer (CT) states in the nonequilibrium regime (i.e., without thermalisation effects). In this paper, we focus on developing automated tools and workflows to investigate excitonic quantum dynamics (QD) in molecular crystals using fragment-based diabatization (FrD) and the linear vibronic coupling (LVC) model, based on electrostatically embedded (EE) excited-state calculations.

Excitonic states can be defined using various methods based on the excited states of individual molecular units.^3,15–17^ Fragment-based diabatization is particularly effective since it projects the TD-DFT adiabatic states of a supramolecular complex onto a quasi-diabatic basis built from the adiabatic states of its molecular units.^12,18,19^ Additional CT states can be arbitrarily defined in terms of the relevant molecular orbitals, providing an intuitive description of key intermolecular processes within the aggregate.^18–21^ Similarly, rigid molecules can be accurately described using LVC Hamiltonians derived from vibrational modes. These models are combined (FrD-LVC) to yield a complete vibronic description of the aggregate.^19^ FrD-LVC can be used also to model the photophysics in solution, by electrostatically embedding the diabatic states with point charges from the solvent molecules.^22,23^ Although not uniquely defined, the diabatic representation is an advantageous framework for modeling exciton dynamics as the nonadiabatic coupling vanishes by definition. Consequently, the physical character of the diabatic states, defined to be equivalent to the adiabatic states at the FC point, is preserved during the dynamics.

Burghardt’s group has pioneered the investigation of excitonic QD with multiconfigurational time-dependent Hartree (MCTDH) simulations in organic semiconductors and interfaces using fragment-based diabatization schemes.^24–26^ Surface hopping nonadiabatic dynamics based on Frenkel exciton models within QM/MM embedding approaches have been implemented and are available in the Newton-X and SHARC codes.^27–29^ Despite these promising advances, there is a lack of general implementations for describing QDin crystalline systems, where molecules are often strongly electrostatically and mechanically coupled to the wider crystal environment. A complete description of the photophysics and photochemistry should indeed account not only for the short-range interactions captured by exciton models but also for the longer-range interactions within the material.

Electrostatic embedding based on Ewald cluster models enables the description of long-range interactions in molecular crystals.^30–36^ In combination with ONIOM­(QM:QM′)-EE, Ewald embedding has been employed to describe excited-state processes and PESs in molecular crystals. A combined approach that integrates these electrostatic embedding techniques into the FrD-LVC Hamiltonian represents a promising route for understanding excitonic behavior in organic/molecular crystalline solids.

In this paper, we report our efforts to provide open-source tools and workflows that enable the description of excited states and their dynamics in MC systems. While our main focus is on molecular crystals, these algorithms are also applicable to the study of MC systems in biological soft materials and biological media. We implemented an interface, fro_overdia.py, between two codes, Overdia^37^ and fromage,^31,38^ to obtain and track exciton states in molecular aggregates within realistic environments. Overdia parametrizes the LVC Hamiltonian^39^ for describing PESs through fragment diabatization based on the maximum overlap criteria,^18,19^ while fromage provides various embedding schemes using ONIOM­(QM:QM′)-EE to account for long-range interactions in complex environments. The resulting Hamiltonian can be used to perform MCTDH quantum dynamics simulations, considering several excited states and many (100+) vibrational modes, thanks to the multilayer implementation (ML-MCTDH).^40–44^ Example calculations using fro_overdia.py are provided in tutorial-overdia/.

We apply these newly implemented tools to investigate the excitonic dynamics of dibenzo­[g,p]­chrysene (DBC). Recently, the photoluminescence spectra of DBC has been characterized both in solution and in the solid state (Figure 1).^45^ DBC shows a large bathochromic shift (≈30 nm, 0.21 eV) going from solid to solution, and similarly, an increase in emission intensity with increasing water fraction with no change in spectral line shape. Clearly, crystallization and aggregation play an important role in the photophysics. DBC has a high degree of rigidity and through-bond conjugation, making it an excellent test system for our interface. Additionally, DBC is an excellent scaffold for functionalization toward optoelectronics, particularly OLEDs.^46–49^

**Figure 1:**
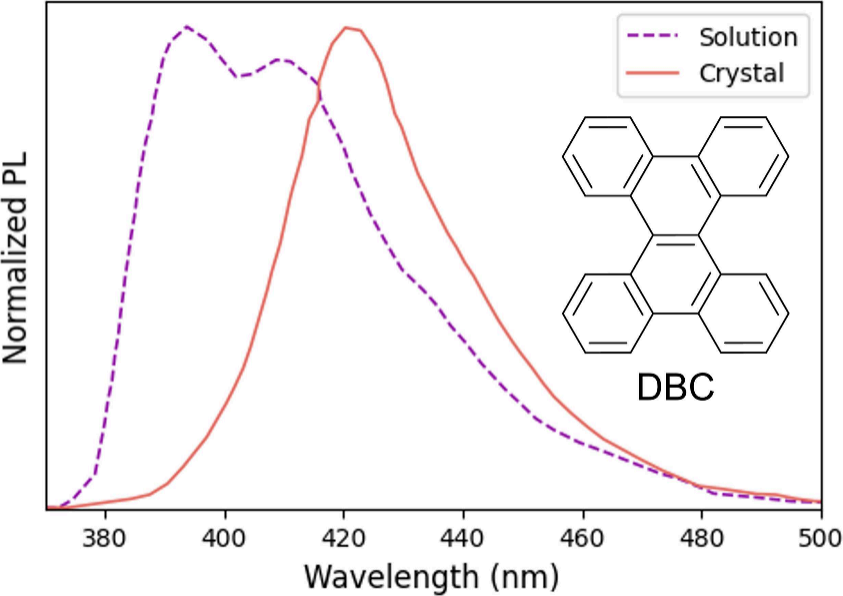
Experimental photoluminescence spectrum of dibenzo­[g,p]­chrysene (DBC, CCDC: 1481424) in dilute THF (10^–5^ M) and crystal. The molecular structure is shown. The spectral data was obtained from ref 45.

The present work is divided into two sections. First, we exhaustively screen dimers within the crystal, using our new interface to rapidly calculate excitonic couplings and energies, leveraging the suite of automated tools in fromage. Second, we identify the dimer in the π-stacked configuration (hereafter referred to as the π-dimer) as the most important dimer in the crystal. On this dimer, we perform ONIOM­(QM:QM′)-EE optimizations (on S_0_ and S_1_) to obtain critical information on the emission properties of the crystal, although the radiative and nonradiative decay rates are not directly computed. Finally, we parametrize an FrD-LVC Hamiltonian with electrostatic embedding (FrD-LVC­(EE)) and perform exploratory QD LVC simulations including 90 vibrational modes, to study the interplay between local excitated states (LEs) and CT states on the most representative DBC dimer, and the effect of the electrostatic field of the crystal on the population dynamics. In this way, we obtain a detailed description of the photophysics of DBC in the solid state, and provide a reproducible protocol which can be rapidly deployed to other molecular crystals, such as those displaying aggregate-induced emission.^50^ Due to the tendency of the excitations to localize, dimeric models should be sufficient to capture the most significant effects ruling the emission process further explanation in Section 4.4). This picture was further confirmed by considering a trimeric model.

## 2 Background Theory

### 2.1 FrD-LVC Hamiltonian Model with Electrostatic Embedding Scheme

As our LVC approach has been described in detail elsewhere,^18,19,39^ we here simply recall the basic constituents of the Hamiltonian. It can be built by considering a set of coupled electronic states in the diabatic basis |**d**⟩ = (|*d*_1_⟩, |*d*_2_⟩, ..., |*d*_*n*_⟩)

1 $$H=\underset{i}{\sum }\left(K\left(p\right)+V_{ii}^{d}\left(q\right)\right)\left|d_{i}\right\rangle \left\langle d_{i}\right|+\underset{i,j>i}{\sum }V_{ij}^{d}\left(q\right)\left(\left|d_{i}\right\rangle \left\langle d_{j}\right|+\left|d_{j}\right\rangle \left\langle d_{i}\right|\right)$$

which depends on the dimensionless normal mode coordinates **q** of the ground electronic state S_0_ and their conjugate momenta **p**. The kinetic *K* and potential *V* terms of the Hamiltonian are defined as

2 $$K=\frac{1}{2}p^{T}\Omega p$$

3 $$V_{ii}^{d}\left(q\right)=E_{ii}^{d}\left(0\right)+\lambda_{ii}^{T}q+\frac{1}{2}q^{T}\Omega q$$

4 $$V_{ij}^{d}\left(q\right)=E_{ij}^{d}\left(0\right)+\lambda_{ij}^{T}q$$

Here, **Ω** is the diagonal matrix of S_0_ vibrational frequencies, *E*_*ii*_^d^(0) is the diabatic vertical energy of state *i* and *E*_*ij*_^d^(0) is a constant electronic coupling between diabatic states *i* and *j*, both at the reference geometry (0). The vectors **λ**_*ii*_ and **λ**_*ij*_, with components λ_*ii*,α_, λ_*ij*,α_ (where α labels the normal mode and *j* ≠ *i*), are the gradients of the diabatic PESs in the reference geometry (**λ**_*ii*_) and the linear coupling parameters between pairs of electronic states along the normal modes that couple them (**λ**_*ij*_). The adiabatic–to-diabatic transformation is obtained by the recently developed FrD-LVC approach.^19,39^ According to this approach, we defined diabatic states of the dimer on the basis of reference states that are either the adiabatic states of the fragments/monomers of the dimer (for LE states) or one-electron transitions between orbitals on different fragments (for CT states). This strategy yields comparable results to property-based diabatization approaches like the Multistate Fragment Exciton Difference-Fragment Charge Difference method^51^ (MS-FED-FCD) and the Fragment Hole–Electron diabatization approach.^16^ In practice, performing the adiabatic-to-diabatic transformation yields a diabatic Hamiltonian matrix, which contains the diabatic energies *E*_*ii*_^d^(0) on diagonal and interstate couplings *E*_*ij*_^d^(0) on off-diagonal. To obtain the linear parameters λ_*ij*,α_ we displace the geometry of the dimer along each dimensionless normal coordinate *q*_α_ by a small amount ± Δ_α_, and we perform a numerical differentiation of the diabatic Hamiltonian. The effect of the crystal environment on the Hamiltonian model was incorporated through an electrostatic embedding scheme, where the point charges of the surrounding environment were included in the calculation of the Hamiltonian parameters, thus obtaining a so-called FrD-LVC­(EE) Hamiltonian Model.

### 2.2 The ONIOM­(QM:QM′)-EE Method

In order to include the effect of the crystal on the different cluster models, we resort to a QM:QM′ approach, where the molecules in the cluster (the model region) are treated with a more accurate QM method, and the remaining molecules (the environment) with a lower level (QM′). The interaction energy is catered for implicitly by the following ONIOM equation,^30,31,52^ by calculating the energy and gradients of the entire system (the real region) at the QM′ level and subtracting an additional QM′ calculation on the model region, to remove the double-counted contributions.

5 $$E_{\mathrm{QM}:\mathrm{QM}\prime }^{\mathrm{EE}}\left(R\right)=E_{\mathrm{QM},\mathrm{model}}^{\mathrm{EE}}\left(r\right)+E_{\mathrm{QM}\prime ,\mathrm{real}}\left(R\right)-E_{\mathrm{QM}\prime ,\mathrm{model}}^{\mathrm{EE}}\left(r\right)$$

The superscript EE denotes electrostatic (point charge) embedding on the model region wave function. The use of a semiempirical method (QM′) enables a high-quality charge distribution to be preserved on the model-high wave function. Here, we use the Ewald method of Derenzo and co-workers^53,54^

6 $$V_{\mathrm{Ewald}}\left(r\right)=\underset{L,s}{\sum }q_{s}\frac{\mathrm{erfc}\left(\gamma \left|r-L-R_{s}\right|\right)}{\left|r-L-R_{s}\right|}+\frac{4\pi }{V_{c}}\underset{G\neq 0}{\sum }\frac{1}{G^{2}}\;\exp\left(-\frac{G^{2}}{4\gamma^{2}}\right)\left[\underset{s}{\sum }q_{s}\;\exp\left(iG\cdot \left(r-R_{s}\right)\right)\right]$$

In this expression, **R**_*s*_ is the unit cell lattice site, γ is the Ewald constant, *V*_*c*_ is the unit cell volume, *q*_*s*_ is the charge at each site and **L** and **G** are the real (**L**) and reciprocal (**G**) lattice translations.^30^ This approach generates a charge distribution (∼10,000 point charges) about the model region converging on the periodic potential, while removing any artificial dipole moment (see ref 30 for our modified Ewald code).

### 2.3 Interfacing Overdia and fromage

One of the goals of this study is to integrate the fromage and Overdia programs, in order to extend the electrostatic embedding techniques (already used profitably in the QD simulation of supramolecular systems in solution)^19^ to crystal structures, utilizing the host of ONIOM methods available in fromage (Figure 2). The interface is now available as a new script, fro_overdia.py, in the latest version of fromage.

**Figure 2:**
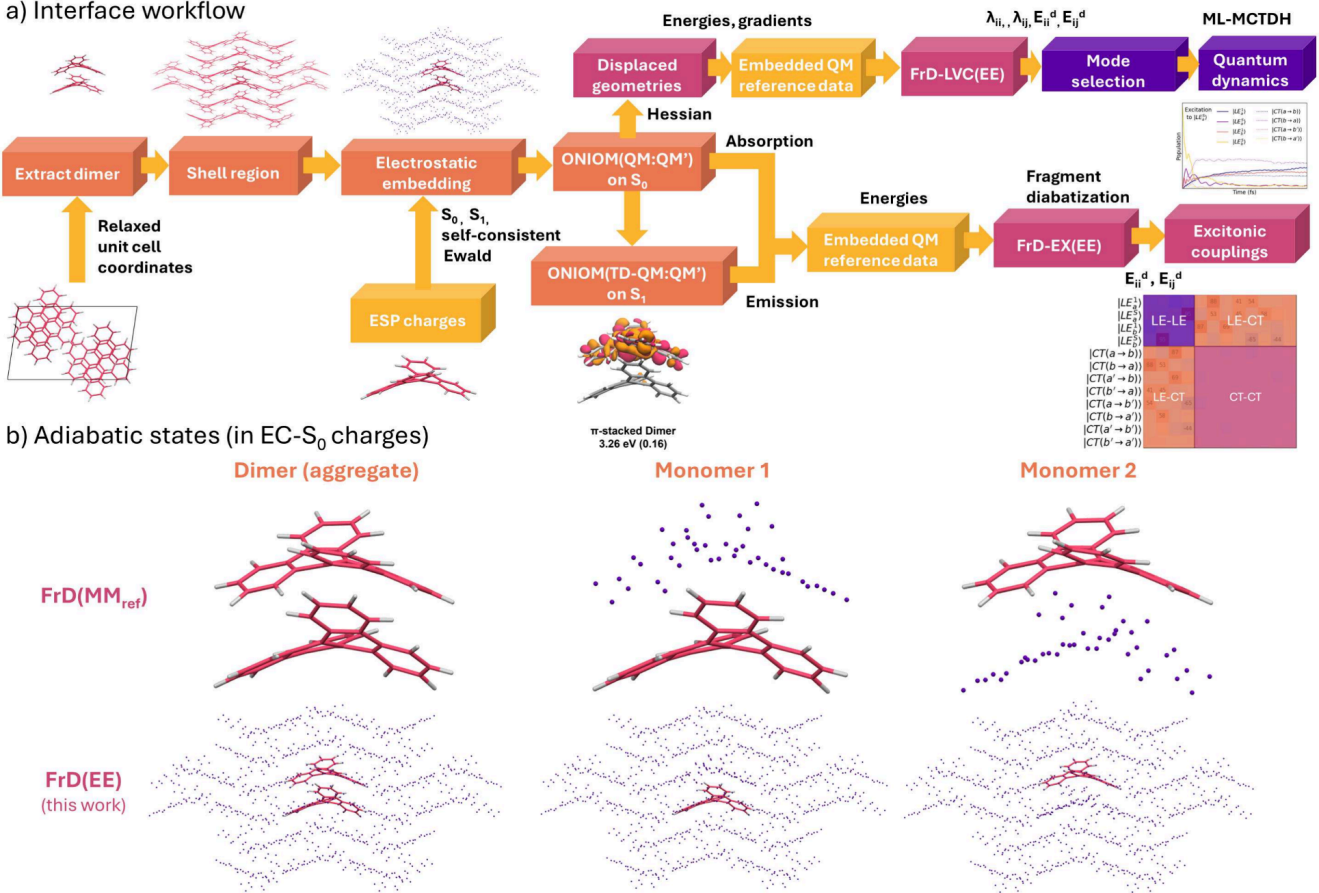
(a) Workflow combining methods in fromage and Overdia, where fromage (orange) is used to automatically select a dimer from the crystal, generate a large cluster region, generate point charge embedding from gas-phase RESP populations, and run embedded cluster ONIOM­(QM:QM′) calculations. Overdia (pink) is used for fragment-based diabatization (FrD), generation of displaced structures along vibrational modes, and the full FrD-LVC­(EE) calculation. Gaussian (yellow) is used externally for the population analyses, the generation of the adiabatic states for diabatization, and the calculation of the normal modes needed for the LVC model. The wavepacket is propagated with Quantics (purple). (b) Visualization of the EC-S_0_ electrostatic embeddingelectrostatic embedding used in the calculation of the adiabatic statesAdditional charges are introduced using the EE methods infromage.

Our combined workflow, shown for the π-dimer as an example, starts from the periodic DFT-relaxed crystallographic coordinates (e.g., from the Cambridge Structural Database). Using automated tools in fromage, the MC dimer is extracted from the unit cell and a large spherical cluster is generated, containing 42 molecules. Next, the MC system is embedded in point charges assigned to each atom in the cluster (i.e., an EC-S_0_ model, see ref 30 for the available point charge embedding schemes), or via the Ewald summation (EEC-S_0_). Using the ONIOM algorithm, the MC system can be relaxed on S_0_ or on S_1_ to investigate absorption or emission, respectively. Finally, the FrD^18^ calculation is performed using the adiabatic states of the dimer and monomers (reference states). The interface generates a TD-DFT input for each calculation. FrD­(EE) indicates the inclusion of electrostatic embedding in this workflow, where the same embedding scheme was used for the monomers and for the dimer. The reference states on the monomer can also be computed by considering the restrained electrostatic potential (RESP) charges (computed either for the ground or the excited states) of the other monomers in the MC (Figure 2b), as it happens in the FrD­(MM_ref_) scheme introduced in ref 19. The diabatic Hamiltonian is then determined as explained above in Section 2.1.^19^

## 3 Computational Details

### 3.1 Periodic Calculations

The experimental crystallographic unit cell of DBC (CCDC: 1481424)^55^ was relaxed using the *Vienna Ab initio Simulation Package* (VASP) with the PBE exchange-correlation functional and the D3 dispersion correction.^56^ The atomic coordinates were relaxed with the conjugate gradient algorithm, where the lattice parameters were fixed to the experimental values. The projector-augmented wave (PAW) method was employed with PBE pseudopotentials. The Brillouin zone was sampled from a Γ-centered 1 × 2 × 1 Monkhorst–Pack *k*-point mesh, with a plane-wave energy cutoff of 500 eV. The SCF cycle was converged to 10^–6^ eV. Symmetry was not used.

### 3.2 Cluster Calculations

All nonperiodic electronic structure calculations were performed with TD-DFT in Gaussian 16 ^57^ unless otherwise specified. The M06-2X (54% HF exchange) exchange-correlation functional was used throughout,^58^ which includes a long-range dispersion correction. The cc-pVDZ basis set was used. This level of theory provides a good balance of cost and accuracy, and comparison to TD-CAM-B3LYP results indicated there was no spurious charge transfer in our models (Figure S4). Tight convergence criteria were used for calculations of the ground and excited states. C_1_ symmetry was used throughout.

For the embedded cluster calculations, ONIOM­(QM:QM′) calculations were performed with fromage, using (TD)-M06-2X/cc-pVDZ as the high-level QM method. Extended Tight-Binding (xTB) was used as the low-level method, with the GFN2-xTB Hamiltonian including the D4 dispersion correction.^59^ This choice of semiempirical method provides approximate DFT accuracy at a much lower computational cost (required by the large size of the system, ≥1800 atoms). For each dimer, large spherical clusters were generated automatically by fromage. Electrostatic embedding was generated by either the EC-S_0_ (embedded cluster) and EEC-S_0_ (Ewald embedded cluster) using RESP charges from an S_0_ population analysis (M06-2X/cc-pVDZ) on the gas-phase DBC monomer (see ref 31 for details). For the ONIOM­(QM:QM′) geometry optimizations, the shell region was fixed to the relaxed crystal geometry, and the model region was optimized with the L-BFGS algorithm, with a convergence threshold of 0.001 eV Å^–1^.

### 3.3 Dimer Screening from Crystal

An initial screening was performed on the crystal geometry to identify key dimeric configurations. Eighteen unique dimers (Figure S1) were screened from the relaxed unit cell using the fro_dimer_tools.py script in fromage.^31,38^ A similarity threshold of 0.0001 Å was used to distinguish equivalent dimers. The centroid distance, contact distance, and slip angle were calculated for each dimer. All the dimers with a distance between centroids smaller than 16 Å were selected, meaning several non-nearest neighbor dimers are included in the set. This enables a systematic investigation of the electrostatic screening induced by molecules located between a given dimer, for instance. The set of dimers is ordered by ascending centroid distance between monomers. An EC-S_0_ model was built for each dimer. The spherical clusters contain between 32 and 44 DBC molecules, depending on the specific dimeric arrangement. RESP point charges (M06-2X/cc-pVDZ) from the ground state were used to generate the embedding. The Frenkel (LE–LE) couplings were estimated for each dimer using three different coupling estimators (V_EET_, V_Coul_, and V_TrESP_) (see Sections S1.1 and S2.1.1 for specific computational details), in addition to diabatisation (V_Diab_). For two of the these approaches, V_TrESP_ and V_Diab_, electrostatic embedding was included, as previously defined (Section 2.3)

### 3.4 FrD Hamiltonian and Diabatic States

For a DBC dimer, we defined 12 diabatic states (Figures S2 and S3 in the SI), i.e. two LEs for each monomer and eight CT states. The two local excitations defined to be identical to the two most intense bright adiabatic excited states of DBC monomer (i.e., S_1_ and S_4_; see below). In the dimer, these are labeled as |LE_*a*_^1^⟩ and |LE_*a*_^S^⟩ (where the superscript ‘S’ stands for strong absorption), depending on the monomer (*a* or *b*) they are localized on. As for the CT states, |CT­(*a* → *b*)⟩, and |CT­(*a* → *b*′)⟩ involve the transfer of an electron from the HOMO of monomer *a* to the LUMO or the LUMO+1 of monomer *b*, respectively. Similarly, |CT­(*a*′ → *b*)⟩, and |CT­(*a*′ → *b*′)⟩ involve the transfer of an electron from the HOMO–1 orbital to the LUMO and to the LUMO+1 orbtials. |CT­(*b* → *a*)⟩ and |CT­(*b* → *a*′)⟩ are the corresponding diabatic states when the electron is transferred from monomer *b* to monomer *a*. Note, a smaller diabatic basis (4 LEs and 2 CTs) was used during the dimer screening, as it was used for check purposes only. Having defined these states, the adiabatic-to-diabatic transformation was performed as described above. The first 60 singlet excited states were used to converge the projection. The visualizations of the CT diabatic states were made using the density difference between the electron density of the LUMO and HOMO, with Multiwfn.^60^

### 3.5 FrD-LVC­(EE) Hamiltonian and Quantum Dynamics of the Wavepacket

To study the photoexcited dynamics of the most representative dimer of the system, in this case Dimer 1 (represented in Figure 3 and Figure S1), we parametrized a full FrD-LVC­(EE) Hamiltonian model, which takes into account the environment effect in the crystal through the electrostatic point charge embedding scheme detailed in Section 2.1. In the approach adopted in this work, vibrational motion is described in terms of the normal modes of the individual fragments. Accordingly, the FrD-LVC­(EE) Hamiltonian was parametrized including only so-called intramolecular vibrational modes, calculated with the normal modes of the individual monomers (i.e., *N* = 240). Consequently, the large-amplitude motions associated with intermolecular vibrational modes, which may be anharmonic and therefore not correctly treated within a LVC model, were excluded. In practice, nuclear dynamics is described using the ensemble of the normal coordinates of the two independent monomers as a basis.

A normal mode analysis was thus performed (including EEC-S_0_ point charges) on the two individual monomers constituting the ground-state minimum structure of the dimer. Then, a set of 2N molecular structures were generated by displacing the equilibrium structure of Dimer 1 by an amount ± Δ_*a*_ = 0.1 along the dimensionless normal coordinates of each individual monomer. The displaced structures were then directly employed to generate the LVC Hamiltonian following a numerical differentiation of the diabatic Hamiltonian, as implemented in the Overdia code.^37^ The LVC Hamiltonian was parametrized using 8 diabatic states, defined in previous section. Of these states, only CT states involving the HOMO, LUMO, and LUMO+1 orbitals were included, as these states showed the lowest diabatic excitation energies and were most likely to be populated during the dynamics. The reduced selection was justified in the QD simulations, where the |CT­(*a* → *b*′)⟩ and |CT­(*b* → *a*′)⟩ were not strongly populated. The electrostatic embedding effect was used in all of the calculations (through point charges), including on the displaced geometries and the reference states (monomers). To investigate the effect of the electrostatic embedding environment, a second FrD-LVC Hamiltonian was parametrized in vacuum by removing the point charges while retaining the same reference and displaced geometries as used for the FrD-LVC­(EE) model. The vibrational frequencies were kept identical to those used in the FrD-LVC­(EE) model. In any case, it should be noticed that the effect of the embedding on the modes is small (≤13%), as shown in the SI (Figure S20).

The QD of the nuclear wavepacket were performed according to the FrD-LVC­(EE) Hamiltonian model, adopting a selected set of 90 vibrational modes for 250 fs, as implemented in the Quantics package.^61^ In fact, the first 90 normal modes with the largest gradients and interstate couplings were selected to run the dynamics, while ensuring a symmetric distribution of equivalent normal modes across the two monomers, for a balanced description. The convergence of the QD simulations with respect to the number of selected modes was also assessed by progressively including vibrational modes with the largest inter- and intrastate couplings. Accordingly, a series of QD simulations was performed using 30, 36, 46, 70, 90, and 110 vibrational modes. As shown in Figures S23–S26, full convergence is reached with 90 modes, and further increasing the number of modes to 110 does not lead to any noticeable change in the population dynamics. Despite the very clear outcome of these convergence tests, we should note that we recover ∼70% of the reorganization energy of the different states, on average, with 110 modes. It is possible that the inclusion of the neglected 130 modes, each bringing a slight contribution, might partially change the final yield of the nonadiabatic transitions on the long-time limit. See Section S2.4 for more details on the ML-MCTDH setup and the graphical representation of the ML trees used for the dimer structure studied in this work.

## 4 Results

According to M06-2X/cc-pVDZ calculations on the monomer (see the SI), the lowest energy excited state (S_1_, ∼3.9 eV) corresponds to a bright ππ* transition, with a major HOMO → LUMO character (a schematic depiction of the associated natural transition orbitals, NTO, can be found in Figure S11 of the SI). S_2_, very close in energy to S_1_, is instead weak, and corresponds to the combination of the HOMO–1 → LUMO and HOMO → LUMO+1 excitations. The most intense transition is S_4_, ∼0.7 eV towards the blue with respect to S_1_. The S_1_ minimum is emissive and it is responsible for the experimental fluorescence spectrum. S_2_ and S_4_ are expected to decay to S_1_ on the ultrafast scale (∼100 fs). As anticipated in the introduction, according to experiments, the emission of DBC is red-shifted from solution to the molecular crystal. To explore the molecular interactions responsible for this outcome, in the first part of this study, we have identified the species exhibiting the strongest intermonomer interactions in the crystal, starting from the dimers.

### 4.1 Fragment Diabatization in 18 DBC Crystal Dimers

In the 18 dimers embedded in the crystal we have examined, the two lowest energy excited states can be described as the combination of the two lowest energy LE of each monomers, with the relative stability of the bright and the weak exciton depending on the J- or H-type aggregation of the specific dimer. The energy shift with respect to the monomer S_1_ transition is quite small (Table S2). Dimer 1 (the π-dimer) stands out, with the lowest energy excited state (in this case the weak exciton) being ∼0.1 eV red-shifted with respect to the average values of the other dimers. Dimer 1 has the smallest centroid distance and is in a π-stacked, face-to-face configuration (Figure S1). The plane of each DBC monomer is oriented so that the overlap between π-electrons is maximized. The adiabatic states of Dimer 1 will be discussed in detail in Section 4.2.

#### 4.1.1 Excitonic Couplings

As the next step, we have computed the excitonic couplings in the 18 dimers identified in the crystal by using different methods (see Section S1.1). In particular we compared the predictions of the FrD method with the excitonic couplings computed based on the monomer transition densities (*V*_EET_), and verified the effect of the crystal electrostatic field on the computed couplings.

Figure 4 summarizes the results of our analysis. For what concerns the excitonic couplings, Fragment diabatization FrD and EET methods provide similar trends. The largest Frenkel coupling between the LEs localized on monomers *a* and *b* is found for Dimer 1, (≈27 meV), roughly twice the size of the next largest coupling found for Dimer 3 (≈15 meV). From the quantitative point of view, in the DBC crystal, the methods considering only Coulomb effects provide a good approximation of the FrD couplings (which also considers the effect of the overlap of the monomer wave functions). Not surprisingly, the quality of this approximation declines when the monomers are closer. For Dimer 1, the coupling predicted by FrD is indeed 10% larger than the *V*_TrESP_ and *V*_Coul_ ones (see Section S1.1). This effect is also observed for |LE_*a*_^S^⟩ – |LE_*b*_^S^⟩ couplings (involving the most intense LEs), where in most cases the magnitude of the coupling is larger (≥100 meV).

**Figure 3:**
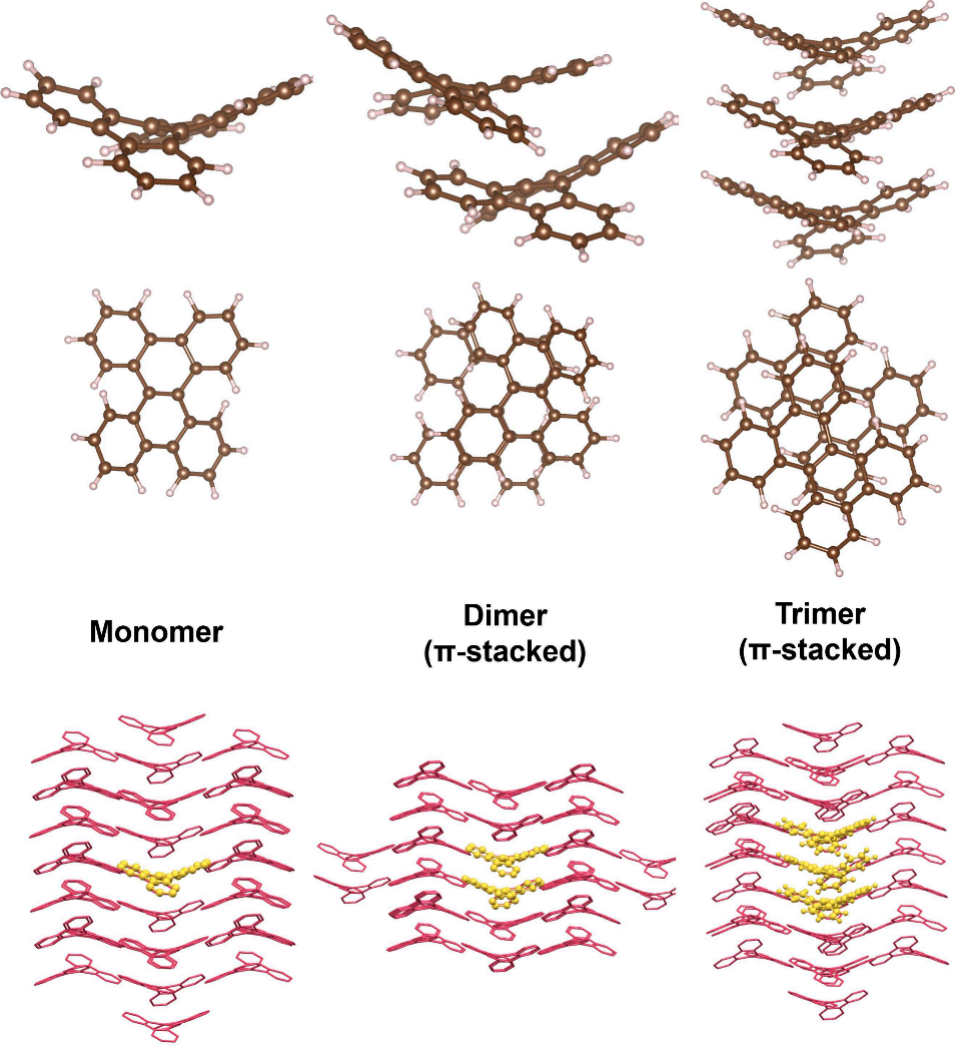
The ONIOM models used in this work (QM: yellow, QM′: pink) For the side view of each cluster, a cross-section is taken to aid visualization. Hydrogens are not shown for clarity. The model region of each cluster is shown above.

**Figure 4:**
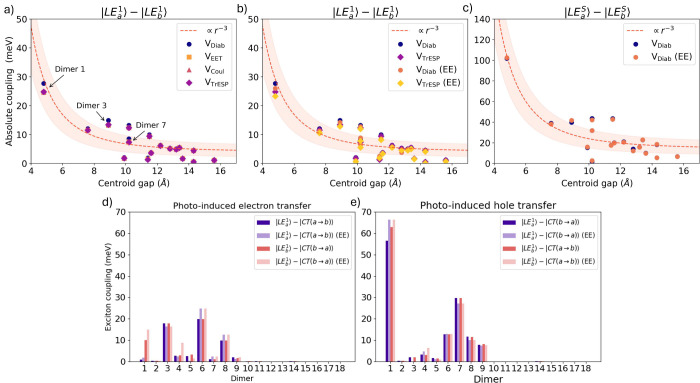
(a–c) Excitonic couplings between S_1_ LEs on each monomer (|LE_*a*_^1^⟩ – |LE_*b*_^1^⟩) and S_4_ LEs on each monomer (|LE_*a*_^S^⟩ – |LE_*b*_^S^⟩), plotted against the centroid distance between monomers. Couplings were calculated with electronic energy transfer (*V*_EET_ and *V*_Coul_, Eqns. S1 and S2), the transition monopole approximation with TrESP charges (*V*_TrESP_, Eqn. S3), and fragment diabatization (*V*_Diab_) calculations. A nonlinear regression fit (∝ *r* ^–3^) is plotted for the *V*_Diab_ couplings, where the shaded region is the standard deviation in the fit on the Overdia data. EE indicates electrostatic embedding with EC-S_0_ RESP charges. (d, e) LE–CT couplings from FrD, divided into LEs coupled to the unoccupied HOMO (photoinduced electron transfer) or the occupied LUMO (photoinduced hole transfer). All calculations were performed with (TD)-M06-2X/cc-pVDZ.

Diabatization further enables couplings between LE and CT states to be obtained in a single calculation and in a consistent manner. The strongest |LE⟩–|CT⟩ interaction is found in Dimer 1 (63 meV), between |LE_*b*_^1^⟩ and |CT­(*a* → *b*)⟩, and is much larger than the related |LE_*b*_^1^⟩ – |CT­(*b* → *a*)⟩ coupling (10 meV). That is to say, photoinduced hole transfer is more likely to occur than photoinduced electron transfer.

Closer inspection of Figure 4 provides interesting insight on the effect of modulating the different hole and electron transfer couplings. First, it is clear that the intercentroid distance is not the only influential parameter. Indeed, as shown more clearly in the Figure S7, dimers with similar intercentroid distance can exhibit coupling parameters differing by 1 order of magnitude. Second, depending on the stacking geometry, photoinduced hole- and electron-transfer coupling parameters can differ substantially. For example, Dimers 7 and 1 show a strong preference for hole transfer with respect to the related electron transfer, whereas Dimer 6 exhibits the opposite trend. As discussed in Section S3.4, the combined analysis of the stacking geometry and the shapes of the overlapping frontier orbitals allows rationalizing of the observed trends.

Electrostatic embedding introduces moderate changes in both the LE–LE and LE–CT couplings. For the larger |LE_*a*_^1^⟩ – |LE_*b*_^1^⟩ couplings (≥5 meV), embedding typically reduces the magnitude by 5–10%, with Dimer 6 showing largest suppression (24%). V_TrESP_(EE) couplings showed a similar reduction. For the much larger couplings between strongly bright states (|LE_*a*_^S^⟩ – |LE_*b*_^S^⟩), the relative effect of the electrostatic embedding was smaller (a decrease of 1–3 meV).

The LE–CT couplings responded less predictably than LE–LE to embedding. Generally, the embedding effect was larger for LE–CT couplings than Frenkel couplings. For instance, in Dimer 1 the |LE_*b*_^1^⟩ – |CT­(*b* → *a*)⟩ increased from 10 to 15 meV, a change of 50%. However, in some cases the embedding also reduced LE–CT couplings. Alternative charge models (based on excited-state charges, Ewald charges, or self-consistent charges) produced similar results to the EC-S_0_ model used here (Figure S8).

Finally, as preliminary exploration of more polar crystals,^35^ in Section S3.4.3, we discuss the results obtained by artificially enhancing the polarity of the charge distribution, showing the dielectric screening effect can also be enhanced with embedding (Figure S9).

### 4.2 The π Stacks

In the previous section, we have shown that the π stacks, those involving Dimer 1 (see Figure 3), are those exhibiting the largest intermonomer electronic couplings. In the next step of our analysis, we shall focus on this arrangement, studying a dimer and a trimer. The mechanical and electrostatic effects of the crystal are included in the ONIOM models. We aim to get additional insights on the effects modulating the spectral properties of DBC in crystals and verify whether the physics of this supramolecular system is sufficient to give account of the experimental trends. To this aim, in the Section S3.4, we also report an analogous discussion of π stacks in the gas phase.

In the FC region, the two lowest energy excited states in the dimer are derived by the combination of the lowest energy excited state of the monomers ( Table 1). The lowest energy one is weak, whereas the other is intense. Analogously, the two following excited states, S_3_ and S_4_, derive from the combination of the second lowest energy excited states of the monomer, and are also very weak. In the trimer, the lowest energy excited state is mainly localized on the ‘central’ DBC, whereas S_2_ and S_3_ mainly correspond to the symmetric/antisymmetric combination of the external DBC LEs.

**Table 1:** Energies (eV) and Oscillator Strength (in Parentheses) of the Lowest Energy Adiabatic States of DBC Monomer, *π*-Dimer, and *π*-Trimer in the Crystal at the S_0_ Optimized Geometry^a^

State	Monomer	Dimer	Trimer
S_1_	3.91 (0.168)	3.82 (0.003)	3.79 (0.012)
**S**_**1**_ **-min**	3.32 (0.249)	3.26 (0.156)	3.23 (0.100)
S_2_	3.95 (0.040)	3.86 (0.227)	3.84 (0.005)
S_3_	4.30 (0.005)	3.92 (0.037)	3.87 (0.243)
S_4_	4.62 (0.840)	3.92 (0.008)	3.89 (0.037)
S_5_		4.18 (0.009)	3.93 (0.014)
S_6_		4.26 (0.001)	3.94 (0.020)
S_7_		4.32 (0.020)	4.13 (0.010)
S_8_		4.43 (0.007)	4.20 (0.002)
S_9_			4.24 (0.002)

a For S_1_, the energy in its optimized minimum is also reported. Each geometry was optimized with ONIOM (QM: (TD)-M06-2X, QM′: xTB) in Ewald charges (EEC-S_0_).

Our Ewald-embedded cluster calculations on the monomer, π-dimer, and π-trimer (Table 1), show a comparable red-shift in both the absorbing and fluorescent geometries. First, for absorption, the vertical transition energy of the π-dimer and π-trimer are red-shifted relative to the monomer by 0.09 and 0.12 eV. Similarly, the S_1_-minima are shifted by 0.06 and 0.09 eV for the dimer and trimer, respectively.

The experimental emission peak lies at 2.95 eV in the crystal and 3.16 eV in solution, indicating that a shift of 0.21 eV should be found in the fully converged model. Moreover, direct visualization of the electronic density difference between S_1_ and S_0_ (Figure 5) reveals that the excited state is localized predominantly on a single DBC molecule. In the case of the dimer and trimer, in the optimized minimum very little electron density is transferred to the neighboring molecules, suggesting these models are generally converged with respect to the crystal, at least for the emissive state. In other words, adding molecules to the aggregate (i.e., a tetramer) would provide only a modest correction at larger computational expense.

**Figure 5:**
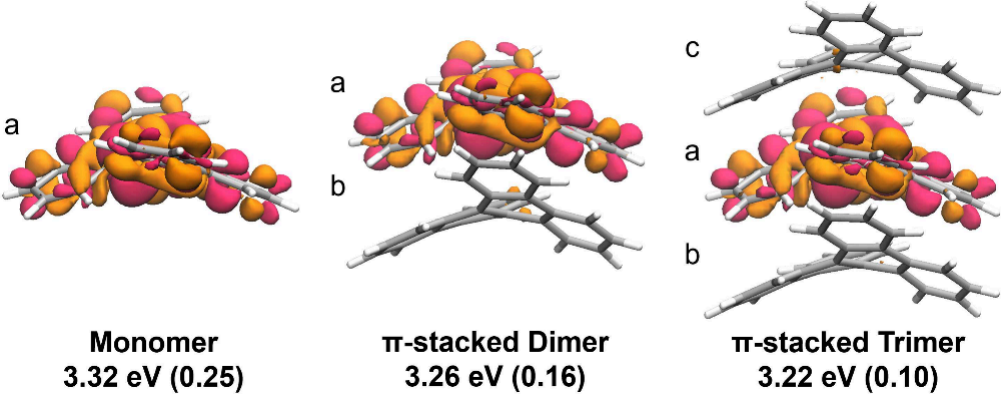
S_1_–S_0_ density difference (ρ = 0.0005) plots at the S_1_ geometry of the DBC monomer, dimer, and trimer in the π stack, obtained from ONIOM-EEC-S_0_ geometry optimizations (QM: M06-2X/cc-pVDZ, QM′: xTB). The monomers in each model are labeled a, b, and c. The S_1_ energy and oscillator strength (parentheses) are reported for each structure (TD-M06-2X).

The results of partial geometry optimizations in the gas phase for a dimer and a trimer (see S4.3), where the DBC monomers keeps the same stacking arrangement adopted in the crystal, fully confirm the conclusions obtained by the ONIOM calculations in the crystal. The excited state minima are essentially localized on a single monomer, though the small delocalization on the stacked partner induces a small red-shift of the absorption and emission energy, which is slightly larger than that obtained in the crystal.

Our calculations on the π stack thus indicate that (i) there is a clear tendency to localize the excitation in the S_1_ minimum, on a single monomer, and that the intermonomer exciton coupling is not large enough to overcome the large reorganization energy associated with localized minima; (ii) at the same time, in the S_1_ minimum there is a small, but nonzero, contribution by the MO of the stacked partners, likely due to the coupling with the CT states, (iii) as a consequence, we observe a ∼0.1 eV red-shift of the emission energy, with a strong decrease of the oscillator strength, on par with experimental observations in Figure 1.

### 4.3 FrD and FrD­(EE) of the π-Dimer

Figure 6 summarizes the results of FrD and FrD­(EE) on the π-dimer (Dimer 1) at three important geometries: the ‘crystal’ one (extracted from the DFT-relaxed unit cell coordinates), the S_0_-ONIOM minimum, and S_1_-ONIOM minimum. This probes the effect of embedding on the diabatic states during absorption and emission, fully utilizing the interface between fromage and Overdia. The S_0_-ONIOM, in contrast to the ‘crystal’ geometry, is locally relaxed, enabling a tighter packing of the dimer. The absolute value of the couplings is generally larger than at the ‘crystal’ coordinates, aside from the largest coupling (|LE_*a*_^S^⟩ – |LE_*b*_^S^⟩).

**Figure 6:**
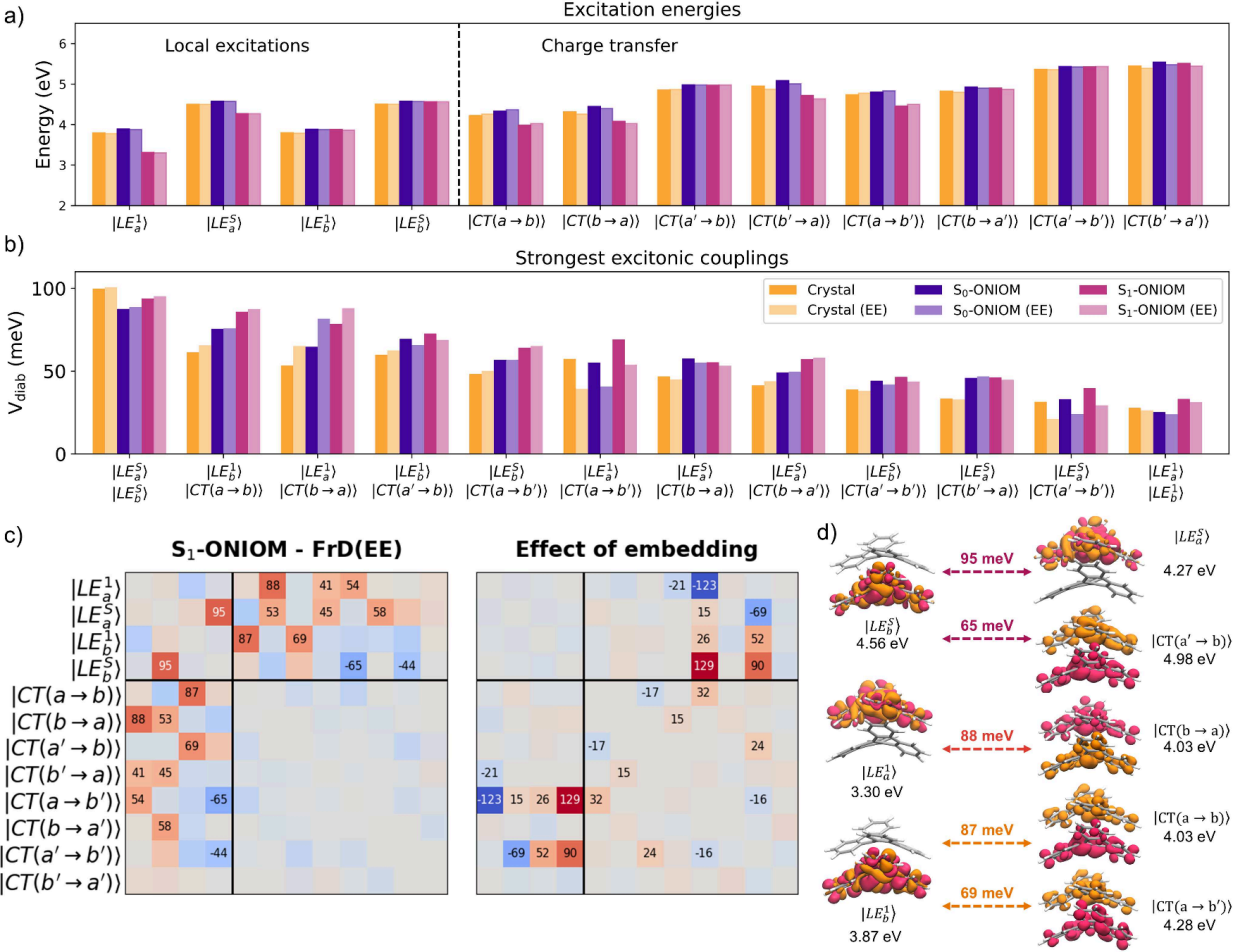
Analysis of the FrD and FrD­(EE) models at three important geometries: crystal (from DFT-relaxed unit cell coordinates), S_0_-ONIOM, and S_1_-ONIOM. (a and b) Excitation energies and the largest excitonic couplings. (c) At the S_1_-ONIOM geometry: Heat map (in meV) of the symmetric FrD­(EE) excitonic couplings minimum and difference matrix between FrD­(EE) and FrD couplings. (d) Excited state density difference plots for the most strongly coupled diabatic states. Orange and pink indicate a negative and positive change in density, respectively.

In contrast, at the (adiabatic) S_1_ minimum of the dimer, the S_1_ excitation is localized on monomer *a*. Consequently, diabatic states involving the HOMO/LUMO orbitals of monomer *a* (which dominate the transition) (|LE_*a*_^1^⟩, |LE_*a*_^S^⟩, |CT­(*a* → *b*)⟩, etc.) are lowered in energy, whereas those states that do not involve these orbitals (|LE_*b*_^1^⟩, |CT­(*a* → *b*′)⟩, etc.) are unchanged with respect to the S_0_-ONIOM. Overall, the couplings are larger than in the ‘crystal’ and S_0_-ONIOM structures. On average, electrostatic embedding changes the larger (≥5 meV) couplings by 10–15%, with the |CT­(*a*′ → *b*)⟩–|CT­(*a*′ → *b*)⟩ coupling reduced by ≥ 50%. Finally, the S_0_-ONIOM of the π-dimer provides similar results to FrD­(EE) on the π-trimer, once again validating the model for describing effects in the crystal (Figure S19).

An analysis of the resulting FrD-LVC­(EE) Hamiltonian in the diabatic representation is shown in Table 2. The diabatic states |LE_*a*_^1^⟩ and |LE_*b*_^1^⟩ and also |LE_*a*_^S^⟩ and |LE_*b*_^S^⟩ are almost degenerate, with the former states being more stable than the latter ones. The same is observed for the CT states, as CT­(*a* → *b*) and CT­(*b* → *a*) and also CT­(*a* → *b*′) and CT­(*b* → *a*′) states are almost degenerate. Interestingly, both CT­(*a* → *b*) and CT­(*b* → *a*) states appeared to be more stable than local states |LE_*a*_^S^⟩ and |LE_*b*_^S^⟩ by ∼0.2 eV. The same trend is also observed in the LVC minima (see Table 3).

**Table 2:** Constant Values of the LVC Hamiltonian in the Diabatic Representation for Dimer 1 at the S_0_-ONIOM Minimum^a^

State	|LE_*a*_^1^⟩	|LE_*a*_^S^⟩	|LE_*b*_^1^⟩	|LE_*b*_^S^⟩	|CT(*a* → *b*)⟩	|CT(*b* → *a*)⟩	|CT(*a* → *b*′)⟩	|CT(*b* → *a*′)⟩
|LE_*a*_^1^⟩	3.879							
	[3.890]							
|LE_*a*_^S^⟩	0.006	4.577						
	[0.002]	[4.577]						
|LE_*b*_^1^⟩	0.024	0.013	3.877					
	[0.025]	[0.009]	[3.886]					
|LE_*b*_^S^⟩	0.003	0.089	0.003	4.573				
	[0.002]	[0.087]	[0.009]	[4.574]				
|CT(*a* → *b*)⟩	0.006	0.034	0.076	0.011	4.373			
	[0.001]	[0.028]	[0.075]	[0.009]	[4.334]			
|CT(*b* → *a*)⟩	0.082	0.055	0.012	0.026	0.001	4.399		
	[0.065]	[0.058]	[0.007]	[0.019]	[0.000]	[4.449]		
|CT(*a* → *b*′)⟩	0.041	0.003	0.019	0.057	0.025	0.002	4.836	
	[0.055]	[0.004]	[0.006]	[0.056]	[0.010]	[0.002]	[4.798]	
|CT(*b* → *a*′)⟩	0.003	0.050	0.019	0.003	0.001	0.012	0.000	4.902
	[0.023]	[0.049]	[0.033]	[0.006]	[0.001]	[0.000]	[0.000]	[4.929]

a Absolute couplings are shown; the coupling-sign relationship between the coupling elements has been checked in Figures S17 and S18. The locally excited (LE) states are defined to correspond to the adiabatic S_1_ and S_4_ bright states of the monomers, parameterized with the FrD-LVC­(EE) and FrD-LVC (in square brackets) Hamiltonian models at the M06-2X/cc-pVDZ level of theory. The matrix is symmetric, and only half is reported. Terms in the diagonal represent the vertical energies, *E*_*ii*_^*d*^(0), while the off-diagonal terms are the constant couplings, *E*_*ij*_^*d*^(0). All terms are in eV.

**Table 3:** LVC Energies (in eV) of Reorganization (ReE), Franck–Condon (FC) and Minimum (Min) of the Eight Diabatic States for DBC Dimer, Where the Locally Excited (LE) States Are Defined as the Adiabatic S_1_ and S_4_ Bright Monomer States, Calculated with the Contributions of 90 Coordinates^a^

Energies	|LE_*a*_^1^⟩	|LE_*a*_^S^⟩	|LE_*b*_^1^⟩	|LE_*b*_^S^⟩	|CT(*a* → *b*)⟩	|CT(*b* → *a*)⟩	|CT(*a* → *b*′)⟩	|CT(*b* → *a*′)⟩
*E* ^FC^	3.879	4.577	3.877	4.573	4.373	4.399	4.836	4.902
	[3.890]	[4.577]	[3.886]	[4.574]	[4.334]	[4.449]	[4.798]	[4.929]
*E* ^ReE^	0.265	0.143	0.253	0.145	0.243	0.228	0.213	0.220
	[0.281]	[0.152]	[0.278]	[0.150]	[0.251]	[0.236]	[0.215]	[0.222]
*E* ^Min^	3.614	4.433	3.624	4.428	4.129	4.171	4.622	4.682
	[3.609]	[4.425]	[3.608]	[4.424]	[4.083]	[4.213]	[4.583]	[4.707]
*f*	0.145	0.685	0.137	0.646	0.000	0.001	0.000	0.000
	[0.156]	[0.696]	[0.149]	[0.656]	[0.000]	[0.001]	[0.000]	[0.000]

a FrD-LVC­(EE) and FrD-LVC (in square brackets) Hamiltonian models including 8 diabatic states parameterized at the M06-2X/cc-pVDZ level of theory.

As far as the linear couplings are concerned, here we focus on those obtained with the FrD-LVC­(EE) parametrization and reported in Table S9 of the SI. As expected, they are systematically much stronger between LEs localized on the same monomer, whereas the couplings between states localized on different monomers are much smaller, often close to zero. A similar trend is observed among the CT states. However, it is interesting to notice that the linear couplings between LE and CT states are relatively larger than those among LEs or among CTs.

### 4.4 ML-MCTDH Quantum Dynamics of the Electronic Population

Finally, we exploited the FrD-LVC­(EE) Hamiltonian to simulate of the photoexcited dynamics of Dimer 1, after exciting the four bright LEs. In Figure 7, we report the population dynamics computed by considering the effect of the crystal on the parameters of the FrD-LVC Hamiltonian. The predicted population dynamics confirms that the driving force toward delocalization is small. As reported in Table 2, at the FC point the two degenerate excitations |LE_*a*_^1^⟩ and |LE_*b*_^1^⟩ are coupled (by −0.024 eV), giving rise to two exciton states. In the perfectly symmetrical case, one would expect the population to relax equally into these exciton states, leading to a 50:50 distribution of population over the two LEs. However, the dynamics shown in Figure 7 reveal that following photoexcitation to either |LE_*a*_^1^⟩ or |LE_*b*_^1^⟩, the population transfer between the two LEs is rather slow, reaching only ∼30% after 250 fs when starting from |LE_*a*_^1^⟩ and ∼40% when starting from |LE_*b*_^1^⟩, thus remaining far from complete delocalization. It is also evident that upon excitation to |LE_*a*_^1^⟩ or |LE_*b*_^1^⟩, the contribution of other diabatic states remains negligible, with their populations staying below 5% throughout the entire dynamics time. The slight asymmetric behavior observed in the population transfer arises from the fact that the two monomers (as part of the same unit cell) are not symmetrically equivalent. This asymmetry is also reflected in the minor differences that exist between corresponding LVC Hamiltonian parameters of the two interacting monomers (see Table 2). Further analysis in Figure S28 shows that the purity of the state decays to less than 0.6 in 250 fs and that coherence between the two most populated states (|LE_*a*_^1^⟩ or |LE_*b*_^1^⟩) is ∼0.05. Interestingly, when the dynamics is initiated from |LE_*a*_^1^⟩, a coherence of comparable magnitude develops with |CT­(*b* → *a*)⟩, even though the latter state remains essentially unpopulated. This behavior arises from the strong coupling between these two states, which induces rapid oscillations in the coherence and gives rise to small-amplitude, high-frequency oscillations dressing the time evolution of the |LE_*a*_^1^⟩ population.

**Figure 7:**
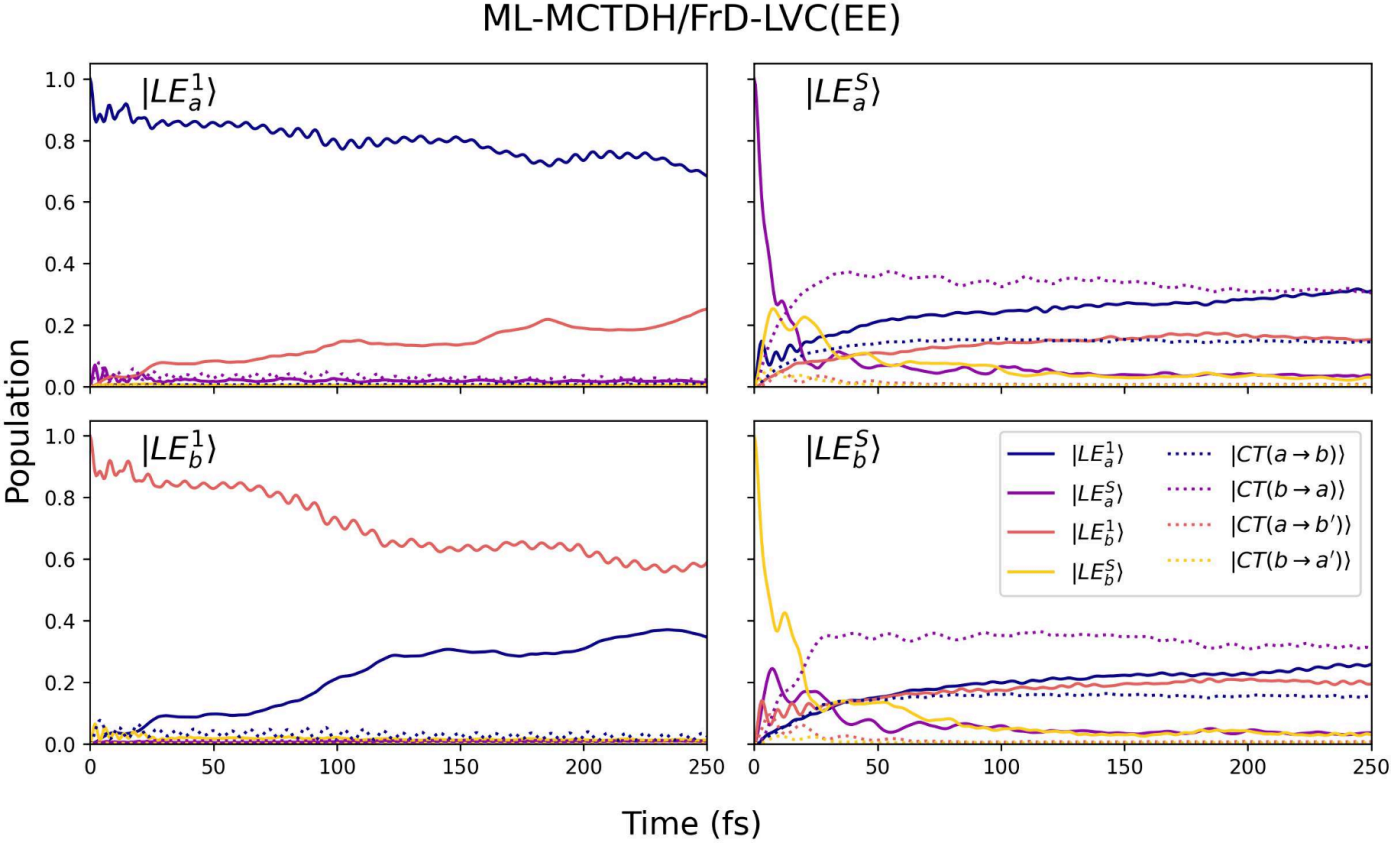
Population dynamics of the diabatic states of the DBC dimer, where the locally excited (LE) states are defined to correspond to the S_1_ (LE^1^) and S_4_ (LE^S^) states of the monomers, following photoexcitation from different initial diabatic states. The simulations are based on a FrD-LVC­(EE) Hamiltonian comprising 8 diabatic states, parametrized at the M06-2X/cc-pVDZ level of theory with Ewald embedding (EEC-S_0_). ML-MCTDH dynamics, including a selected set of 90 normal modes.

When exciting |LE_*a*_^S^⟩ or |LE_*b*_^S^⟩, we observe a strong involvement of CT states. In the initial stage, a noticeable population appears in the complementary LE state (i.e., |LE_*b*_^S^⟩ when exciting |LE_*a*_^S^⟩, and vice versa) due to their large interstate coupling (0.089 eV). This population rapidly decays into |LE_*a*_^1^⟩ and |LE_*b*_^1^⟩, and more prominently into the two CT states. After 100 fs, the population of the most intense LEs drops below 0.1, while |CT­(*b* → *a*)⟩ emerges as the dominant diabatic state, independently of the initially excited LE, accumulating ∼40% of the population. This CT state is also the one most strongly coupled to |LE_*a*_^S^⟩ and |LE_*b*_^S^⟩. Some small differences, arising from the partial structural asymmetry, are again observed in the dynamics initiated from the corresponding states |LE_*a*_^S^⟩ and |LE_*b*_^S^⟩ localized on different monomers. In particular it is worth noting that, in the long-time limit, |LE_*a*_^1^⟩ becomes the most populated LE state both when we excite on monomer *a* (|LE_*a*_^S^⟩) and on monomer *b* (|LE_*b*_^S^⟩). This finding is in line with the fact that, as discussed previously, exciting |LE_*b*_^1^⟩ we have more transfer to |LE_*a*_^1^⟩ than in the opposite process. Some memory of the initial excitation however still survives and, in fact, the transfer of population to |LE_*a*_^1^⟩ is predicted to be slightly larger from |LE_*a*_^S^⟩ than from |LE_*b*_^S^⟩. Finally, for completeness, we also examined the QD involving the weak local excitations (S_2_ on each monomer, see Figure S29).

Exciting the LEs of a single monomer is a convenient way to study photoinduced energy and charge transfer; however, it does not accurately represent the initial state created by photoexcitation in the crystal. While a precise simulation of this process would require a dedicated study, to assess the sensitivity of the dynamics to a delocalized excitation, we also examine the time evolution following excitation of each of the two bright states of the dimer. More precisely, we performed QD simulations photoexciting to combinations of the diabatic states, corresponding to the two lowest bright adiabatic LVC states at the FC position, namely, S_2_ and S_6_ (see Table S8). It is noteworthy that the new delocalized states are combinations of diabatic states with fixed coefficients. In other words, they correspond to the adiabatic states at the FC point, but remain diabatic in nature. The true adiabatic states depend on the coordinates making the computation of truly adiabatic populations much more challenging. The QD simulations (Figure S27) reveal that photoexcitation to S_2_ leads to ultrafast (∼5 fs) population of S_1_ (∼50%). Quite interestingly, the population is ∼50% even on both |LE_*a*_^1^⟩ and |LE_*b*_^1^⟩. Moreover, coherence between S_2_ and S_1_ is practically zero and coherence between LE_*a*_^1^ and LE_*b*_^1^ starts from 0.5 and drops to around 0.05 in ∼5 fs. consequently, the purity of the state drops to 0.5, the value for an incoherent mixture of two states (Figure S28).

Finally, the influence of the crystal environment on the population dynamics can be assessed by inspecting Figure 8, where the FrD-LVC Hamiltonian parameters were computed in the absence of electrostatic embedding. Comparison of diabatic energies and couplings in vacuum with those including the crystal environment reveals only minor differences. As expected, the most noticeable effect concerns the CT states. In the absence of electrostatic embedding, the population of |CT­(*b* → *a*)⟩ is slightly larger, particularly within the first 100 fs. The minor differences in population transfer do not strongly affect the overall photophysical properties. For instance, we obtain nearly identical absorption spectra with and without electrostatic embedding (Figure S22). More prominent effects on the wavepacket would be expected when there is a larger electrostatic effect (i.e., polar systems) or when geometric effects are fully accounted for (i.e., full relaxation at the FC point).

**Figure 8:**
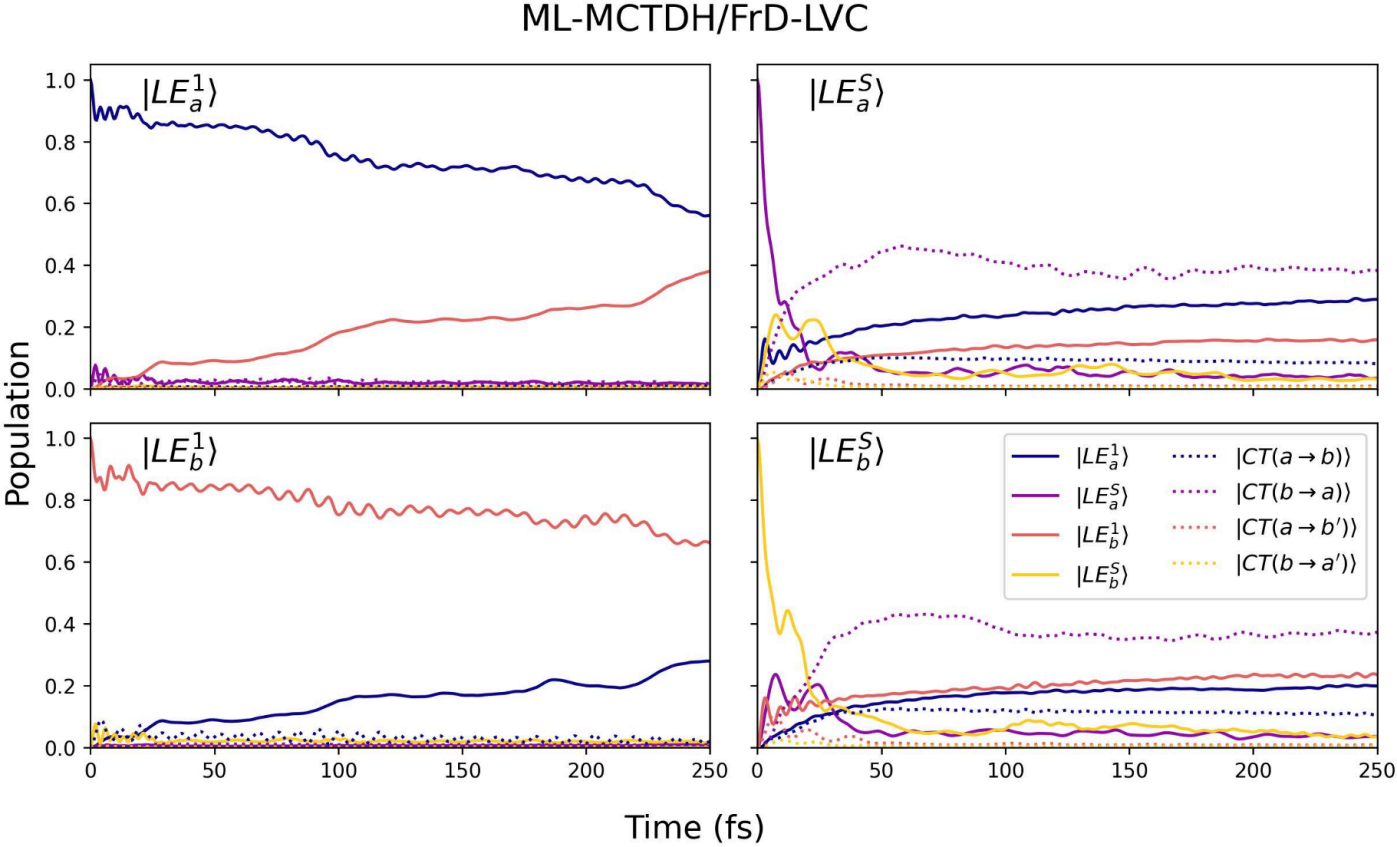
Population dynamics of the diabatic states of the DBC dimer obtained from an 8-state FrD-LVC diabatic Hamiltonian, parametrized at the M06-2X/cc-pVDZ level of theory without electrostatic embedding. The ML-MCTDH simulations include a selected set of 90 normal modes.

## 5 Concluding Remarks

In this work, we propose a computational strategy for studying the photoactivated dynamics of organic molecular crystals, aimed at integrating some of the tools developed for molecular systems with those more routinely applied in materials science. We have therefore described a new interface between the fromage and Overdia programs, extending the capabilities of the latter for the study of QD in molecular crystals. The integration provides an adaptable framework tailored to scenarios in which excitations are relatively localized in a molecular crystal (i.e., when the couplings are significantly smaller than the reorganization energies, *V*_diab_ < *E* ^ReE^), but are still appreciably coupled (mechanically and/or electrostatically) to the wider crystal environment. In contrast to traditional models based on periodic boundary conditions, we employed molecular cluster models combined with the ONIOM­(QM:QM′) methodology implemented in fromage to parametrize vibronic models with Overdia, which is more suitable for capturing the tendency of excitations to localize. The effect of long-range electrostatic interactions in the molecular crystal is included via an Ewald embedding model based on RESP charges. The fragment-based diabatization scheme implemented in Overdia enables the systematic investigation of the interplay between energy- and charge-transfer processes, which are often crucial for understanding the properties of organic semiconducting materials. This combined approach ultimately provides a framework for studying the coupled dynamics of vibrations and electronic states at the QD level in photoexcited crystal-like models, operating within the weak to intermediate coupling regimes.

We employed this new methodology to carry out a comprehensive study of the photophysics of DBC crystals, using time-dependent density functional theory calculations for the QM region. We compared the excitonic couplings and excitation energies across a large set of crystal dimers identified with fromage, where we built FrD and FrD­(EE) models to characterize the coupling between the lowest-energy local bright excitations and charge transfer between the fragments. We validated our approach for the Frenkel couplings by comparison with methods based on transition densities (*V*_EET_, *V*_Coul_, *V*_TrESP_), and additionally characterized LE–CT couplings. Our inclusion of electrostatic embedding had a modest but non-negligible effect (10–20%) on the excitonic couplings, and can be further enhanced, depending on the the charge distribution.

The most strongly interacting dimer is characterized by its π-stacked configuration, where S_1_ corresponds to a weak exciton and S_2_ to the first bright exciton. Using ONIOM­(QM:QM′) calculations, we optimized both the monomer and the dimer in the S_0_ and S_1_ states within the crystal environment. At the emissive S_1_ minimum, although the excitation is essentially localized on a single monomer, the small couplings with intermonomer CT states lead to a slight red shift in the emission energy, fully consistent with that observed experimentally when moving from solution to crystal. As similar behavior is observed when keeping the same geometry but repeating the computations in the gas phase, this effect arises not from the electrostatic field created by the crystal but from the geometrical arrangement of the dimers imposed by the crystalline structure. ONIOM­(QM:QM′) geometry optimization of a π-trimer fully confirms the picture obtained for the dimer, showing localization of the excitation on a single monomer accompanied by a slight red shift in the emission energy.

For the π-stacked dimer model, we parametrized a full LVC Hamiltonian capable of describing both intramonomer and intermonomer couplings between different local excitations, as well as couplings to various CT states. This was based on the normal modes of each monomer embedded in point charges, using a selected set of 90 normal modes. The analysis was performed both with Ewald embedding and in vacuum, allowing us to probe the effect of electrostatic embedding; QD simulations were carried out in both cases. Following photoexcitation to the |LE^1^⟩ diabatic states (S_1_ on each monomer), some population transfer occurred between monomers, with minimal participation from other diabatic states. In contrast, photoexcitation to the very bright |LE^S^⟩ states (S_4_ on each monomer) led to ultrafast decay (∼100 fs), primarily to the |CT­(*a* → *b*)⟩ state, along with local excitations on both monomers. The slight asymmetry observed in the QD results arises from asymmetry in the π-stacked model. It is worth noting that, although we limited ourselves here to parametrizing full LVC models for a dimer as a proof of concept (following similar approaches for other systems^21^), the new interface between Overdia and fromage can, in principle, be used to generate models for larger oligomers if needed to study the system’s physics.

This workflow has broader implications for excited-state nonadiabatic dynamics in molecular crystals, connecting the tools used by the molecular and crystal communities. While approaches for obtaining embedded diabatic Hamiltonians have been explored previously,^51,62,63^ the wide range of highly tunable embedding schemes in fromage opens up many new avenues, depending on the photophysical properties of a molecular crystal. When absorption is mostly a localized process, the long-range crystal electrostatics are accurately captured by the Ewald embedding approach. Similarly, for a highly excited crystal, our workflow can generate embedding based on S_1_ point charges, creating a scenario in which an excitation is localized within an environment of excited chromophores. By using localized molecular models in crystal studies, this approach could be particularly useful for investigating defects or impurities in crystals.^64^ Future work will build on this protocol to study materials in the weak to intermediate coupling regimes.

## Supplementary Material



## Data Availability

Data, including geometries and input files for FrD­(EE), vibrational analysis, FrD-LVC parametrization, and QD calculations, are available at 10.5281/zenodo.18299223.

## Notes

Additional computational details of the static calculations and QDs (PDF)
