# Supplementary material for: Diabatization with Electrostatic Embedding for Studying Photophysics in Organic Molecular Crystals
Source: J Chem Theory Comput. 2026 Feb 23;22(5):2427–41. doi: 10.1021/acs.jctc.5c01934 (PMC12980709; doi:10.1021/acs.jctc.5c01934)
Supplement: Supplementary file 1 [file ct5c01934_si_001.pdf]

Supporting Information: Diabatization with electrostatic embedding for studying photophysics in organic molecular crystals

Michael Ingham,[a] Mohammad Aarabi,[b] Samuele Giannini,[c] Marco Garavelli,[b] Fabrizio Santoro,*[d] Roberto Improta,*[e] and Rachel Crespo-Otero*[a]

[a]*Department of Chemistry, University College London, 20 Gordon Street, WC1H 0AJ London, United Kingdom*

[b]*Dipartimento di Chimica Industriale "Toso Montanari", Universitá di Bologna - Alma Mater Studiorum, Via Piero Gobetti 85, 40129 Bologna, Italy*

[c]*Department of Chemistry and Industrial Chemistry, University of Pisa, Via Giuseppe Moruzzi, 56124 Pisa, Italy*

[d]*Consiglio Nazionale delle Ricerche, Istituto di Chimica dei Composti Organo Metallici (ICCOM-CNR), I-56124 Pisa, Italy*

[e]*Istituto di Biostrutture e Bioimmagini-CNR (IBB-CNR), Via De Amicis 95, I-80145 Napoli, Italy*

E-mail: fabrizio.santoro@pi.iccom.cnr.it, roberto.improta@cnr.it, r.crespo-otero@ucl.ac.uk

Contents

S1 Background theory **S3**

S1.1 Excitonic coupling . . . S3

S2 Computational details **S4**

S2.1 Dimer screening models . . . S4

S2.1.1 Coupling estimators . . . S4

S2.2 Diabatic states . . . S5

S2.3 Level of theory . . . S7

S2.4 ML-MCTDH setting . . . S7

S3 Crystal dimer screening **S9**

S3.1 Adiabatic states . . . S9

S3.2 Diabatic states . . . S10

S3.3 Excitation energies . . . S10

S3.4 Excitonic couplings . . . S11

S3.4.1 Influence of electrostatic embedding . . . S13

S3.4.2 Effect of Ewald, S_1, and self-consistent electrostatic embedding schemes . . . S13

S3.4.3 Effective charge screening (V_{eff}) in DBC π-stack . . . S13

S4 DBC monomer and π stacks **S16**

S4.1 The π stacks in the crystal (No embedding) S16

S4.2 Natural transition orbitals S17

S4.3 Partial geometry optimizations S20

S4.4 FrD and FrD(EE) calculations on the π-stacked dimer S22

S4.5 Consistency of adiabatic eigenstates between FrD-LVC and FrD-LVC(EE) models S23

S4.6 FrD(EE) on the π Trimer S25

S5 FrD-LVC and quantum dynamics **S26**

S5.1 Monomer properties S26

S5.2 FrD-LVC(EE) and quantum dynamics involving the strong ($|LE^S\rangle$) local excitations S27

S5.3 Normal mode convergence S29

S5.4 Photoexciting adiabatic states S35

S5.5 FrD-LVC(EE) and quantum dynamics involving the weak ($|LE^W\rangle$) local excitations S36

S1 Background theory

S1.1 Excitonic coupling

The excitonic coupling, V_{ab}, between chromophores plays a key role charge transport and energy transfer in crystals.[1] The size of the coupling compared to the reorganization energy determines if energy transfer occurs within the weak coupling regime (dipole-dipole interactions) or the strong coupling regime (fully delocalised excitations). In addition to diabatization, a number of approaches can be used to calculate the coupling.[2]

Firstly, the approach from transition densities provides a formal description of the exciton coupling using the transition densities from TD-DFT calculations on each fragment (ρ^T).[3,4] In this approach, long-range effects are accounted for by the Coulomb integral, and the exchange-correlation kernel, g_{XC}, introduces additional short-range quantum effects. The parameter ω_0 is the resonance frequency of the unperturbed states. As such, the exciton couplings are naturally decomposed into contributions from the Coulomb, exchange, and wavefunction overlap.

$$\begin{aligned} V_{\text{EET}} &= \iint_{ab} \rho_a^{T*}(\mathbf{r}) \left[\frac{1}{|\mathbf{r}-\mathbf{r}'|} + g_{XC}(\mathbf{r},\mathbf{r}',\omega_0) \right] \rho_b^{T}(\mathbf{r}')\, d\mathbf{r}\, d\mathbf{r}' - \omega_0 \int \rho_a^{T*}(\mathbf{r})\, \rho_b^{T}(\mathbf{r})\, d\mathbf{r} \\ &= V_{\text{Coul}} + V_{\text{XC}} + V_{\text{overlap}} \end{aligned} \tag{S1}$$

In general, the long-range Coulombic term dominates short-range effects (i.e. $V_{\text{ab}} \approx V_{\text{Coul}}$) in the EET calculation, particularly at distances over 5 Å,[5]

$$V_{\text{Coul}} = \iint_{ab} \frac{\rho_a^{T*}(\mathbf{r})\rho_b^{T}(\mathbf{r}')}{|\mathbf{r}-\mathbf{r}'|}\, d\mathbf{r}\, d\mathbf{r}' \tag{S2}$$

Consequently, low-cost coupling estimators target the Coulomb integral. The point-dipole approximation (PDA) is the simplest approach, using just the transition dipole moment of each fragment and the centroid distance between fragments, but significantly overestimates the couplings at small intermolecular distances. Instead, the transition monopole approximation (TMA) provides a more accurate approach, using transition electrostatic potential (TrESP) charges (based off of the transition density of each fragment), q, which are assigned to atomic sites (i and j) in each fragment. The resulting V_{TrESP} couplings are comparable to the transition density cube (TDC) method.[6]

$$V_{\text{TrESP}} = \sum_{i \in a} \sum_{j \in b} \frac{q_i q_j}{|\mathbf{r}_i - \mathbf{r}_j|} \tag{S3}$$

In the main text, we compare V_{EET}, V_{Coul}, and V_{TrESP} couplings to the constant couplings obtained from results from our fragment diabatization model. Indeed, the transition density (used to derive the TrESP charges for each fragment) can be calculated in the presence of electrostatic (point charge) embedding. Consequently, we use this a reference for our FrD(EE) model.

S2 Computational details

S2.1 Dimer screening models

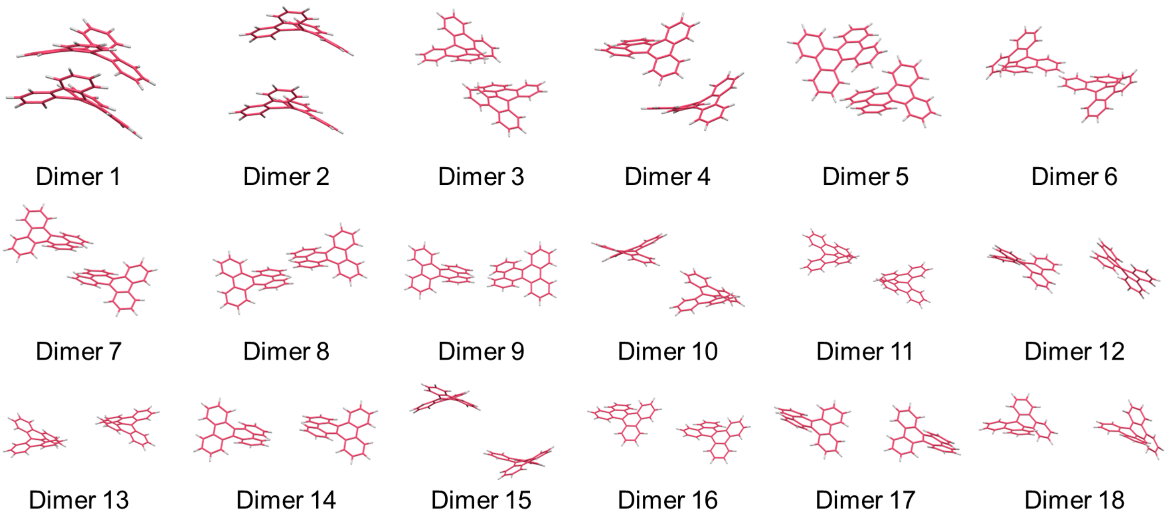

Figure S1: 18 unique dimers screened from relaxed unit cell, ordered by ascending centroid distance.

S2.1.1 Coupling estimators

Firstly, the approach from the transition densities was performed using the `EET` keyword in Gaussian16. This provides the total excitonic couplings (V_{Tot}, Eqn. S1).[7] The two reference states were defined as the monomers in either dimer. Ten singlet excited states were included in the EET calculation. The Coulomb (V_{Coulomb}, Eqn. S2) term from V_{Tot} was read from the EET calculation output.

The V_{TrESP} coupling was evaluated from the TMA (Eqn S3) using an in-house script. The TrESP charges were fitted to atoms in each monomer using the transition density of the S_1 state with the ground state, calculated using Gaussian16 for each monomer. For the V_{TrESP} couplings with electrostatic embedding, this fitting was calculated in the presence of EC-S_0 charges, which were in turn generated with `fromage`. Finally, for the fragment diabatization approach (V_{Diab}), 45 singlet excited states were calculated for each dimer, and 8 single states for the each monomer. This number of excited-states was converged with respect to the diabatic projection. The diabatization was performed with `Overdia`.[8–10] The $|LE_a^1\rangle$, $|LE_b^1\rangle$, $|LE_a^S\rangle$, $|LE_b^S\rangle$, $|CT(a \rightarrow b)\rangle$, and $|CT(b \rightarrow a)\rangle$ states were used in the diabatization, as defined in Figures S2 and S3, and the main text. In the resulting diabatic Hamiltonian, the off-diagonal elements indicate the coupling between diabats, and the diagonal elements give the excitation energies. Finally, the dimer screening was repeated with electrostatic embedding for the V_{TrESP} and V_{Diab} approaches using the FrD(EE) diabatization. The charge distribution was generated by a EC-S_0 charge distribution for each shell region.

S2.2 Diabatic states

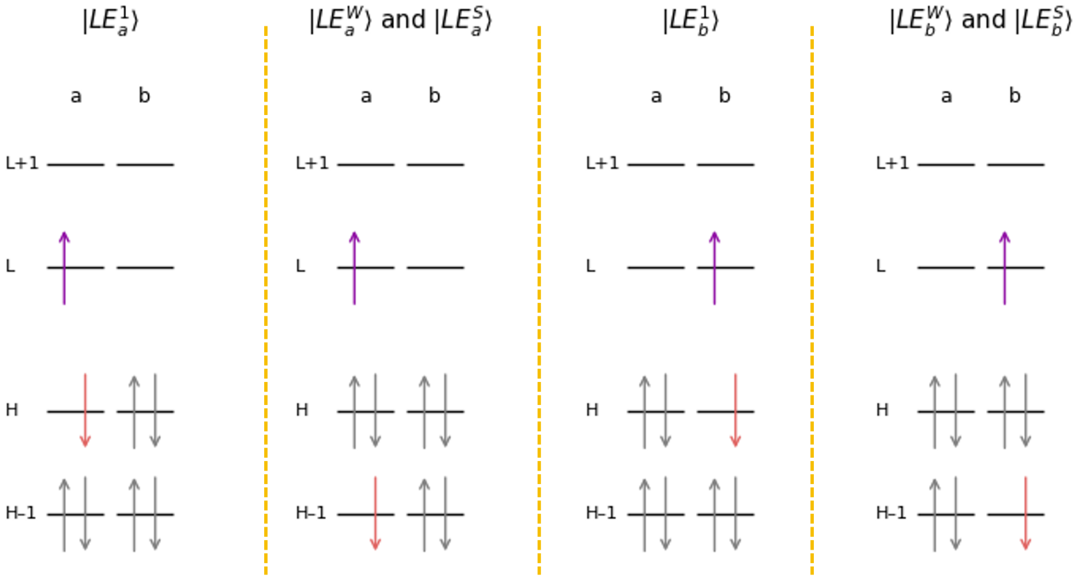

Figure S2: Schematic diabatic states for local excitations on each monomer (a and b). The diagram shows the dominant orbital transition for S_1 $|LE_i^1\rangle$, S_2 $|LE_i^W\rangle$, and S_4 $|LE_i^S\rangle$. The S_4 and S_2 excited states are symmetric and antisymmetric linear combinations of the same transitions.

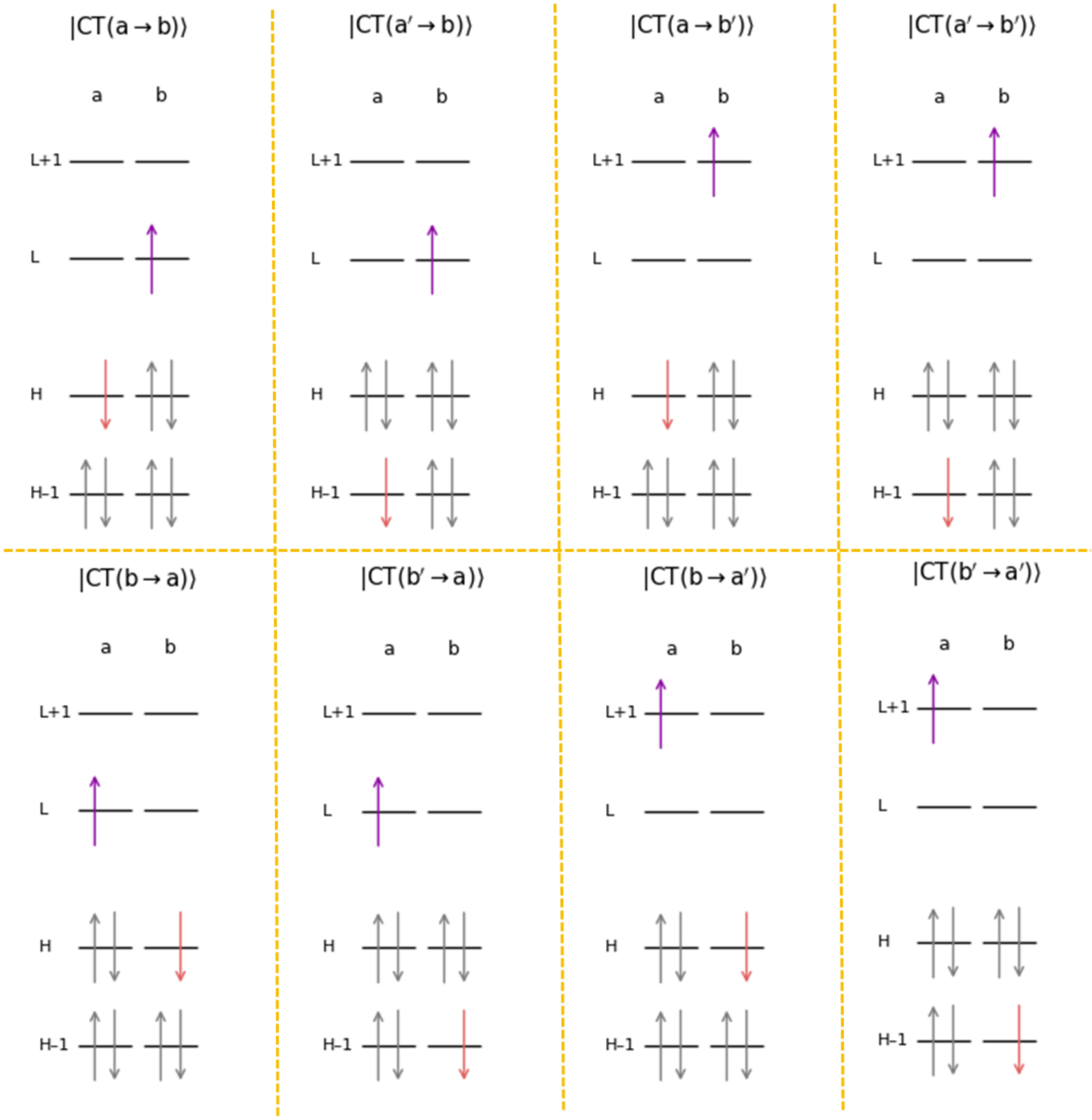

Figure S3: Schematic diabatic states for charge transfer states between monomers a and b.

S2.3 Level of theory

This combination of basis set and functional was chosen to balance accuracy and computational cost. Illustrated for Dimer 1 at the unit cell coordinates (no embedding), use of a triple-ζ basis or adding diffuse functions does not substantially change the relative energies of the first 10 excited states (Figure S4). However, the larger basis sets substantially increase the computational cost. Finally, M06-2X provides similar results to CAM-B3LYP, a range-separated hybrid functional, indicating no spurious charge transfer is present.

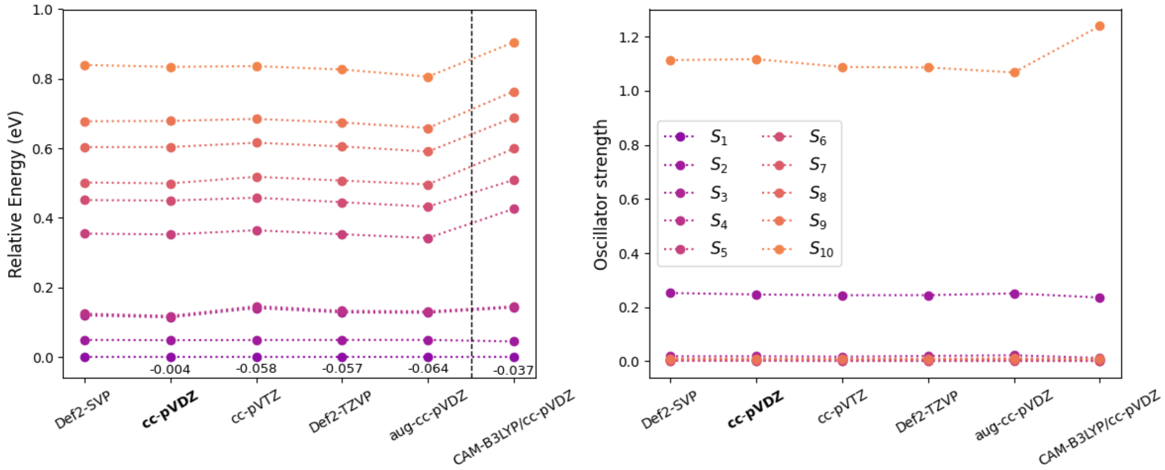

Figure S4: Relative energies and oscillator strength for Dimer 1 (no embedding) calculated a range of basis sets and with TD-CAM-B3LYP. Each energy is annotated with the energy difference (in eV) from the M06-2X/Def2-SVP S_1 calculation.

S2.4 ML-MCTDH setting

We used a variable mean field (VMF) with a Runge-Kutta integrator of order 5 and accuracy 10^{-7}. For the primitive basis set, we adopted Hermite DVR functions. Convergence of the QD propagations was checked by monitoring the populations at the beginning and end of the grid using the rdgpop tool provided in Quantics package, ensuring that they did not exceed 10^{-9}. Further convergence checks were done by monitoring the eigenvalues of the density matrices of each node in the ML tree, ensuring that the smallest natural weight was always $< 1\%$ as indicated in the Quantics manual. Finally, convergence was also confirmed by changing the number of SPFs and the dimension of the primitive basis set. The graphical representation of the ML-MCTDH tree used for DBC dimer is shown in Figures S5.

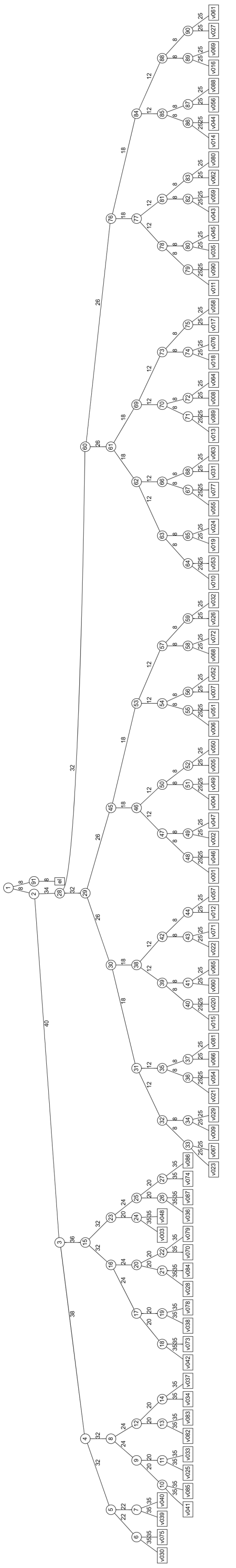

Figure S5: Graphical representation of the ML-MCTDH tree adopted in the QD computations of the DBC π-dimer. The numbers close to the lines indicate the single particle functions used to combine each mode or the number of primitive functions to describe each normal coordinate.

S3 Crystal dimer screening

S3.1 Adiabatic states

Table S1: Adiabatic states (S_1 to S_8) of the 18 dimers screened from the crystal (TD-M06-2X/cc-pVDZ) without embedding.

Dimer	S_1	f	S_2	f	S_3	f	S_4	f	S_5	f	S_6	f	S_7	f	S_8	f
1	3.73	0.00	3.78	0.25	3.84	0.02	3.85	0.00	4.08	0.00	4.18	0.01	4.23	0.01	4.33	0.01
2	3.81	0.00	3.83	0.32	3.87	0.00	3.87	0.01	4.23	0.00	4.23	0.00	4.51	0.00	4.59	1.57
3	3.80	0.52	3.82	0.00	3.87	0.01	3.87	0.00	4.22	0.00	4.22	0.00	4.48	0.00	4.57	1.65
4	3.81	0.38	3.82	0.02	3.87	0.01	3.87	0.01	4.22	0.00	4.23	0.00	4.52	1.85	4.55	0.03
5	3.80	0.52	3.83	0.00	3.87	0.01	3.87	0.01	4.23	0.00	4.23	0.00	4.50	0.03	4.57	1.58
6	3.80	0.00	3.82	0.37	3.87	0.02	3.87	0.00	4.23	0.00	4.23	0.00	4.49	2.10	4.58	0.00
7	3.80	0.45	3.82	0.00	3.87	0.00	3.87	0.02	4.23	0.00	4.23	0.00	4.53	0.00	4.54	1.74
8	3.81	0.39	3.82	0.00	3.87	0.00	3.87	0.02	4.23	0.00	4.23	0.00	4.53	1.87	4.57	0.00
9	3.81	0.01	3.83	0.34	3.87	0.02	3.87	0.01	4.23	0.01	4.23	0.00	4.50	2.10	4.59	0.02
10	3.81	0.41	3.82	0.00	3.87	0.00	3.87	0.02	4.23	0.00	4.23	0.00	4.53	0.03	4.57	1.64
11	3.81	0.43	3.82	0.00	3.87	0.00	3.87	0.02	4.23	0.00	4.23	0.00	4.53	0.00	4.57	1.66
12	3.81	0.00	3.82	0.35	3.87	0.02	3.87	0.00	4.23	0.00	4.23	0.00	4.54	1.82	4.57	0.02
13	3.81	0.42	3.82	0.00	3.87	0.00	3.87	0.02	4.23	0.00	4.23	0.00	4.54	0.00	4.57	1.68
14	3.81	0.00	3.82	0.35	3.87	0.02	3.87	0.00	4.23	0.00	4.23	0.00	4.53	1.92	4.57	0.00
15	3.82	0.39	3.82	0.00	3.87	0.00	3.87	0.02	4.23	0.00	4.23	0.00	4.54	0.03	4.56	1.68
16	3.82	0.39	3.82	0.00	3.87	0.02	3.87	0.00	4.23	0.00	4.23	0.00	4.55	1.80	4.56	0.00
17	3.81	0.00	3.82	0.36	3.87	0.02	3.87	0.00	4.23	0.00	4.23	0.00	4.53	1.89	4.57	0.00
18	3.82	0.39	3.82	0.00	3.87	0.02	3.87	0.00	4.23	0.00	4.23	0.00	4.54	1.79	4.56	0.03

Table S2: Adiabatic states (S_1 to S_8) of the 18 dimers screened from the crystal (TD-M06-2X/cc-pVDZ) with EC-S_0 embedding.

Dimer	S_1	f	S_2	f	S_3	f	S_4	f	S_5	f	S_6	f	S_7	f	S_8	f
1	3.72	0.00	3.77	0.23	3.84	0.03	3.84	0.00	4.07	0.01	4.18	0.00	4.23	0.01	4.34	0.01
2	3.80	0.00	3.82	0.30	3.87	0.00	3.88	0.04	4.22	0.00	4.22	0.01	4.51	0.00	4.59	1.55
3	3.79	0.50	3.81	0.00	3.87	0.04	3.87	0.00	4.21	0.01	4.21	0.00	4.48	0.00	4.57	1.63
4	3.81	0.33	3.81	0.05	3.87	0.04	3.87	0.01	4.21	0.01	4.22	0.00	4.52	1.84	4.56	0.02
5	3.79	0.50	3.82	0.00	3.87	0.03	3.87	0.01	4.22	0.00	4.22	0.01	4.50	0.03	4.57	1.56
6	3.79	0.00	3.81	0.36	3.87	0.00	3.87	0.05	4.21	0.03	4.22	0.00	4.49	2.08	4.58	0.00
7	3.79	0.43	3.81	0.00	3.87	0.00	3.87	0.04	4.21	0.00	4.22	0.02	4.53	0.00	4.54	1.71
8	3.81	0.38	3.81	0.00	3.87	0.00	3.87	0.04	4.22	0.00	4.22	0.01	4.53	1.85	4.57	0.00
9	3.80	0.01	3.82	0.32	3.87	0.00	3.87	0.06	4.22	0.02	4.22	0.01	4.50	2.06	4.59	0.02
10	3.81	0.39	3.81	0.00	3.87	0.02	3.87	0.02	4.22	0.00	4.22	0.01	4.53	0.03	4.57	1.62
11	3.80	0.41	3.81	0.00	3.87	0.04	3.87	0.00	4.22	0.00	4.22	0.02	4.53	0.00	4.57	1.64
12	3.80	0.01	3.81	0.33	3.87	0.02	3.87	0.03	4.22	0.01	4.22	0.01	4.54	1.79	4.57	0.02
13	3.81	0.40	3.81	0.00	3.87	0.04	3.87	0.00	4.22	0.00	4.22	0.01	4.54	0.00	4.57	1.66
14	3.80	0.00	3.81	0.33	3.87	0.05	3.87	0.00	4.22	0.02	4.22	0.00	4.53	1.89	4.57	0.00
15	3.81	0.37	3.81	0.00	3.87	0.02	3.87	0.03	4.22	0.01	4.22	0.01	4.54	0.03	4.56	1.66
16	3.81	0.31	3.81	0.06	3.87	0.04	3.88	0.01	4.22	0.01	4.22	0.00	4.55	1.78	4.56	0.00
17	3.81	0.00	3.81	0.34	3.87	0.00	3.87	0.05	4.22	0.02	4.22	0.00	4.53	1.86	4.57	0.00
18	3.81	0.31	3.81	0.06	3.87	0.04	3.87	0.00	4.22	0.01	4.22	0.01	4.55	1.76	4.56	0.03

S3.2 Diabatic states

S3.3 Excitation energies

The excitation energy of each diabatic state is important as only states close in energy are likely to interact. Table S3 shows the excitation energies of each diabatic state at the crystal geometry, in vacuum (FrD) and with electrostatic embedding (Frd(EE)). At the FrD level, the LE energies on each monomer remain generally invariant to the conformation of the dimer. Dimer 1 has the lowest energy LE states, with $|LE_a^1\rangle$ and $|LE_b^1\rangle$ at 3.78 and 3.80 eV, respectively. For comparison, these states lie at 3.81-3.82 eV in the other dimers, showing the geometry only has a small effect on the local excitations of each monomer. In contrast, the CT states are stabilised in the π-stacked dimer (Dimer 1), where closer stacking lowers the CT energies significantly. The most stable states $|CT(a \to b)\rangle$ (4.22 eV) and $|CT(b \to a)\rangle$ (4.29 eV). For the other 17 dimers, these states lie in the range 4.71-5.17 eV. Generally, the energies of CT($a \to b$) and CT($b \to a$) are similar in energy, however in Dimer 1, charge transfer from the HOMO of monomer a to the LUMO of monomer b, $|CT(a \to b)\rangle$, is lower in energy (4.22 eV) than the reverse transition (4.29 eV). This likely arises from some small asymmetry of the stacking arrangement. The distribution of excitation energies is shown in Figure S6.

Table S3: Excitation energies (in eV) of LE and CT states in the diabatic basis for each dimer at the relaxed crystal geometry (TD-M06-2X/cc-pVDZ). Values computed in vacuum (FrD) are reported, with the corresponding electrostatically embedded (FrD(EE)) values shown in parentheses.

Dimer	Centroid distance (Å)	Slip angle (°)	Excitation energy (eV)			
			$\lvert LE_a^1\rangle$	$\lvert LE_b^1\rangle$	$\lvert CT(a \to b)\rangle$	$\lvert CT(b \to a)\rangle$
1†	4.8	29.2	3.79 (3.78)	3.80 (3.79)	4.22 (4.28)	4.29 (4.22)
2	7.6	9.2	3.82 (3.81)	3.82 (3.81)	4.75 (4.80)	4.74 (4.68)
3†	8.9	78.1	3.81 (3.80)	3.81 (3.80)	4.76 (4.76)	4.76 (4.76)
4†	9.9	58.5	3.81 (3.81)	3.82 (3.81)	4.99 (4.91)	4.71 (4.78)
5†	10.2	76.5	3.82 (3.81)	3.82 (3.81)	4.90 (4.84)	4.87 (4.91)
6†	10.2	56.5	3.82 (3.81)	3.82 (3.81)	4.88 (4.88)	4.88 (4.88)
7†	10.2	82.3	3.81 (3.80)	3.81 (3.80)	4.83 (4.83)	4.83 (4.83)
8†	11.4	57.7	3.82 (3.81)	3.82 (3.81)	4.96 (4.95)	4.96 (4.95)
9†	11.5	75.7	3.82 (3.81)	3.82 (3.81)	4.93 (4.90)	4.95 (4.97)
10	11.6	55.7	3.82 (3.81)	3.82 (3.81)	4.99 (5.03)	5.02 (4.98)
11	12.2	74.3	3.82 (3.81)	3.82 (3.81)	5.03 (5.02)	5.03 (5.02)
12	12.8	56.7	3.82 (3.81)	3.82 (4.01)	5.03 (5.05)	5.07 (5.05)
13	13.2	71.6	3.82 (3.81)	3.82 (3.81)	5.08 (5.07)	5.08 (5.07)
14	13.4	73.7	3.82 (3.81)	3.82 (3.81)	5.07 (5.06)	5.07 (5.06)
15	13.6	43.7	3.82 (3.81)	3.82 (3.81)	5.11 (5.10)	5.10 (5.10)
16	14.3	67.2	3.82 (3.81)	3.82 (3.81)	5.10 (5.10)	5.13 (5.13)
17	14.3	75.0	3.82 (3.81)	3.82 (3.81)	5.11 (5.10)	5.11 (5.10)
18	15.6	66.8	3.82 (3.81)	3.82 (3.81)	5.17 (5.16)	5.16 (5.15)

† Nearest neighbors

In the FrD(EE) calculations, electrostatic embedding has a small effect on the LEs states, reducing their excitation energies by ≤ 0.01 eV for the LEs across all dimers. However, the effect of the embedding is more pronounced. For instance, in Dimer 1, the electrostatic embedding reverses the relative stability of CT states. At the FrD level, the $|CT(a \to b)\rangle$ is lower in energy than $|CT(b \to a)\rangle$, however this ordering is

reversed under electrostatic embedding. This highlights the importance of accounting for polarization effects in the crystal when considering charge transfer.

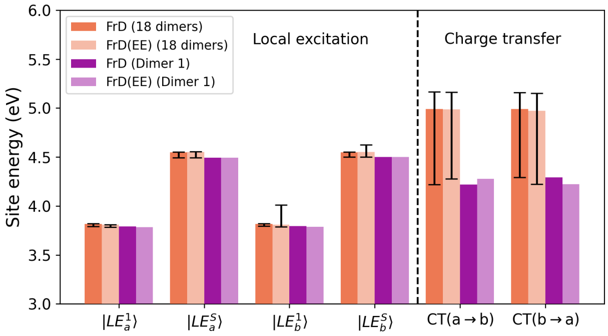

Figure S6: Median excitation energies (in eV) of local excitation (LE) and charge-transfer (CT) states in the diabatic basis, computed using the FrD and FrD(EE) models. Error bars represent the minimum and maximum energies of each state across all dimers. The specific case of the π-stacked dimer (Dimer 1), which has the lowest-energy CT states, is also shown.

S3.4 Excitonic couplings

Excitonic couplings were calculated using the estimators outlined in Section S1.1 and compared to those obtained from diabatization. Table S4 shows that the largest Frenkel coupling between the $|LE_a^1\rangle$ and $|LE_b^1\rangle$ states (i.e. the S_1 on each monomer, f $\approx$ 0.2) occurs in Dimer 1 ($\approx$ 27 meV), roughly twice the size of the next strongest, Dimer 3 ($\approx$ 15 meV). The enhanced coupling of Dimer 1 is due to the π-stacked configuration, which features a slip angle of 30°and centroid gap of 4.8 Å. The π-stacking provides maximum overlap between the monomer wavefunctions, and therefore favors the formation of delocalised excitonic states. Dimer 2 is also from the π-stack, but its monomers are not nearest neighbors, being separated by a DBC molecule, and the centroid distance is 7.6 Å. Nevertheless, the Frenkel coupling is still appreciable ($\approx$ 12 meV) and larger than most of the other dimers. In general, dimers from the π-stack, or with some partial overlap of π-systems, have stronger couplings those in side-to-side configurations. For instance, Dimer 8 is in the side-to-side configuration, has a very low excitonic coupling (1-2 meV). This suggests some interactions in the π-stack occur over more than one molecule in the DBC crystal.

For each bright state, the inter-centroid distance generally governs the magnitude of the coupling, following the $|V| \propto r^{-3}$ relationship observed in organic molecular crystals.[1] This trend is illustrated is as a fitted curve in the figure. This dependence is also shown in Frenkel couplings between the intense bright states of each monomer (i.e., S_4, f≈1). The lowest bright adiabatic state (S_1 or S_2) of each dimer varies depending on the alignment of the transition dipole moments (TDMs) (i.e. H- or J-type aggregation). Across all dimers, the CT states exhibit TDM with the ground state very close to zero, whereas the LEs of the first bright state are $\approx$ 1.4 eV.

Interestingly, the LE-CT couplings vary with centroid distance, and show a greater dependence on the closest intermolecular C-C contact between between monomers (Figure S7a). Only nearest neighbor dimers have strong LE-CT couplings. However, within this subset of dimers (3.3−4.2 Å), the LE-CT couplings

Table S4: Excitonic couplings in the diabatic basis (TD-M06-2X/cc-pVDZ) for each dimer at the relaxed crystal geometry in vacuum. The values of the diabatization with point charge embedding are shown in parentheses.

Dimer	Centroid distance (Å)	Slip angle (°)	V_{Diab} (meV)				
			$\|LE_a^1\rangle$ -$\|LE_b^1\rangle$	$\|LE_a^1\rangle$ -$\|CT(a \rightarrow b)\rangle$	$\|LE_b^1\rangle$ -$\|CT(b \rightarrow a)\rangle$	$\|LE_a^1\rangle$ -$\|CT(b \rightarrow a)\rangle$	$\|LE_b^1\rangle$ -$\|CT(a \rightarrow a)\rangle$
1†	4.8	29.2	-27.7 (-26.0)	0.9 (1.8)	-9.9 (-15.0)	56.6 (66.4)	62.9 (66.4)
2	7.6	9.2	12.0 (11.2)	0.3 (0.4)	0.3 (0.2)	-0.3 (-0.3)	-0.3 (-0.2)
3†	8.9	78.1	14.9 (14.2)	-17.8 (-16.4)	-17.8 (-16.4)	1.9 (-0.0)	1.9 (-0.0)
4†	9.9	58.5	2.1 (1.0)	2.8 (2.2)	3.0 (3.0)	-3.2 (-4.8)	-3.1 (-6.5)
5†	10.2	76.5	-13.2 (-12.8)	-2.5 (0.2)	-3.1 (-1.3)	-1.7 (-1.0)	-1.4 (-0.9)
6†	10.2	56.5	8.4 (8.9)	-19.9 (-24.7)	-19.9 (-24.7)	12.7 (12.9)	12.7 (12.9)
7†	10.2	82.3	-8.5 (-6.5)	1.1 (2.3)	1.1 (2.3)	-29.7 (-27.1)	-29.7 (-27.1)
8†	11.4	57.7	1.9 (2.5)	9.7 (12.5)	9.7 (12.5)	-11.5 (-10.0)	-11.5 (-10.0)
9†	11.5	75.7	10.0 (8.5)	-1.9 (-1.2)	-1.7 (-2.0)	7.7 (7.4)	8.1 (7.7)
10	11.6	55.7	-3.8 (-3.5)	0.0 (0.0)	0.1 (0.1)	0.0 (-0.0)	0.0 (0.0)
11	12.2	74.3	6.3 (5.8)	-0.1 (0.0)	-0.1 (0.0)	-0.0 (-0.0)	-0.0 (-0.0)
12	12.8	56.7	5.3 (3.8)	0.0 (0.0)	0.0 (0.0)	-0.0 (-0.0)	-0.0 (-0.0)
13	13.2	71.6	5.1 (4.8)	0.0 (0.0)	0.0 (0.0)	-0.0 (-0.0)	-0.0 (-0.0)
14	13.4	73.7	-5.6 (-5.2)	0.1 (0.1)	0.1 (0.1)	-0.1 (-0.1)	-0.1 (-0.1)
15	13.6	43.7	-1.7 (-1.7)	0.0 (-0.0)	-0.0 (-0.0)	0.0 (-0.0)	-0.0 (0.0)
16	14.3	67.2	-0.6 (-0.1)	-0.0 (-0.0)	-0.0 (-0.0)	-0.0 (-0.0)	-0.0 (-0.0)
17	14.3	75.0	-4.6 (-4.2)	0.0 (0.0)	0.0 (0.0)	-0.0 (-0.0)	-0.0 (-0.0)
18	15.6	66.8	1.2 (0.8)	-0.0 (0.0)	-0.0 (-0.0)	-0.0 (-0.0)	-0.0 (-0.0)

† Nearest neighbors

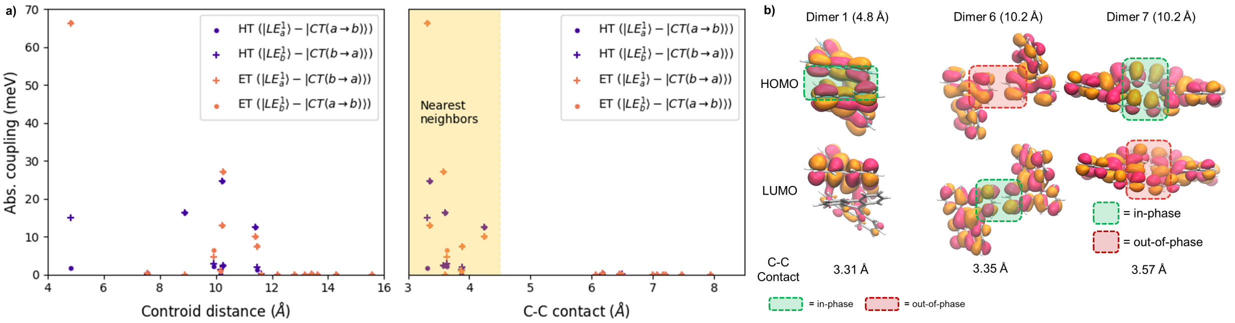

Figure S7: a) Density plots of the HOMO and LUMO of Dimers 1, 6, and 7, where the centroid gap is given in parentheses. The visualizations are annotated with the phase of the orbitals between monomers. The shortest distance between carbon atoms (C-C contact) is also shown. b) Absolute LE-CT couplings plotted in terms of the centroid distance between dimers, and divided into the photo-induced electron transfer (ET, orange) and hole transfer (HT, purple).

vary widely large (0.01−66 meV). For instance, Dimers 6 and 7 exhibit very different HT and ET couplings, despite having similar Frenkel couplings (6−8 meV), a similar centroid distance (10.2 Å), and similar contact distances (3.3−.5 Å). Dimer 7 shows much stronger photo-induced electron transfer (∼ 30 meV) than hole transfer (∼ 1 meV), whereas in Dimer 6 these couplings are relatively similar (12-19 meV).

Based off transfer integrals between orbitals, larger HT couplings arise from significant overlap between monomer HOMOs, and ET between LUMOs. As such, if the monomer orbitals are overlapping in-phase or out-of-phase determines the strength of the coupling. Consequently, Figure S7b suggests the much larger HT coupling in Dimer 7 arises due to overlapping HOMO orbitals, as can be seen from inspection of orbital plots. Similarly, for Dimer 6, the ET coupling is 7 meV larger from in-phase LUMO interaction, where the closer contact point (3.35 Å) contributes to larger couplings in both cases. The case is more complex for Dimer 1 as the orbitals are more strongly mixed. There is a strong in-phase interaction between HOMO orbitals, explaining the strong hole transfer coupling (∼ 63 meV).

S3.4.1 Influence of electrostatic embedding

Figure 4 shows that electrostatic embedding has a modest influence on the couplings. For the appreciable $|LE_a^1\rangle$–$|LE_b^1\rangle$ couplings ($\geq$5 meV), electrostatic embedding typically reduces the coupling magnitude by approximately 5–10%, with the largest reduction observed in Dimer 6 (24%). This trend holds for couplings obtained from both V_{TrESP}(EE) and V_{diab}(EE) approaches, where the inclusion of electrostatics lowers couplings by 1–2 meV, with the largest decrease again found in Dimer 1. V_{TrESP}(EE) were the smallest couplings across all estimation methods. As is expected, the couplings between strongly bright excitations ($|LE_a^S\rangle$-$|LE_b^S\rangle$) were much larger (i.e. $\geq$100 meV for dimer 1). The difference between the V_{diab} and V_{diab}(EE) couplings is still around around 1-2 meV, resulting in smaller relative differences between the FrD and FrD(EE) methods. Overall, both TrESP and diabatisation approaches provide comparable results when electrostatic couplings is included. This suggests that in organic crystals with polar molecules, both approaches should benefit form the inclusion of point charges.

For the couplings larger than 5 meV, the embedding typically modified the LE-CT couplings by $\leq$ 20% compared to FrD calculation. Whether this effect increased or decreased the couplings was less predictable. In Dimer 1, coupling between $|LE_b^1\rangle$ and $|CT(b \rightarrow a)\rangle$ rose from 10 to 15 meV, a 50% increase. Similarly, the couplings of Dimer 4 doubled with electrostatic embedding. A large shift in the excitation energy of $|LE_b^1\rangle$ was observed, explaining the larger coupling. In combination with other factors, these data suggest electrostatic embedding can have a substantial impact on both the coupling between local excitations and charge transfer states. As we will see later on, changes of this nature can influence quantum dynamics simulations. Additionally, the RESP charges from the DBC monomer are very small (-0.21 to 0.15 e); the polarizing effect of the environment will be larger in organic molecular crystals containing heteroatoms.

S3.4.2 Effect of Ewald, S_1, and self-consistent electrostatic embedding schemes

Figure S8 shows the excitation energies and excitonic couplings obtained from FrD(EE) diabatisation under alternative point charge embedding schemes implemented in `fromage`. Overall, the effect of changing the charge distribution is small. However, when using S_1 charges (i.e. point charges derived from a population analysis of the S_1 density), we observe a lower energy $|CT(a \rightarrow b)\rangle$ state and higher energy $|CT(b \rightarrow a)\rangle$ state than when using S_0 charges. The use of self-consistent charges (i.e. allowing the charge distribution to relax) leads to smaller couplings, although the change is small. The small differences between diabatic states was anticipated as the adiabatic states under each distribution were very similar. Nevertheless, in certain organic molecular crystals, it is essential to tune the charge distribution to obtain accurate adiabatic states (See Ref. 11 for a detailed analysis in the X23 molecular crystal database).

S3.4.3 Effective charge screening (V_{eff}) in DBC π-stack

The effect of electrostatic coupling to the environment should be larger in more polar systems (i.e. those containing heteroatoms). Similarly, a dielectric screening effect should be present between between local excitations. Consequently, we built a test to artificially increase the polarity of the charge distribution, and investigate the effect on this effective screening. First, a 14-molecule π-column was extracted from the relaxed

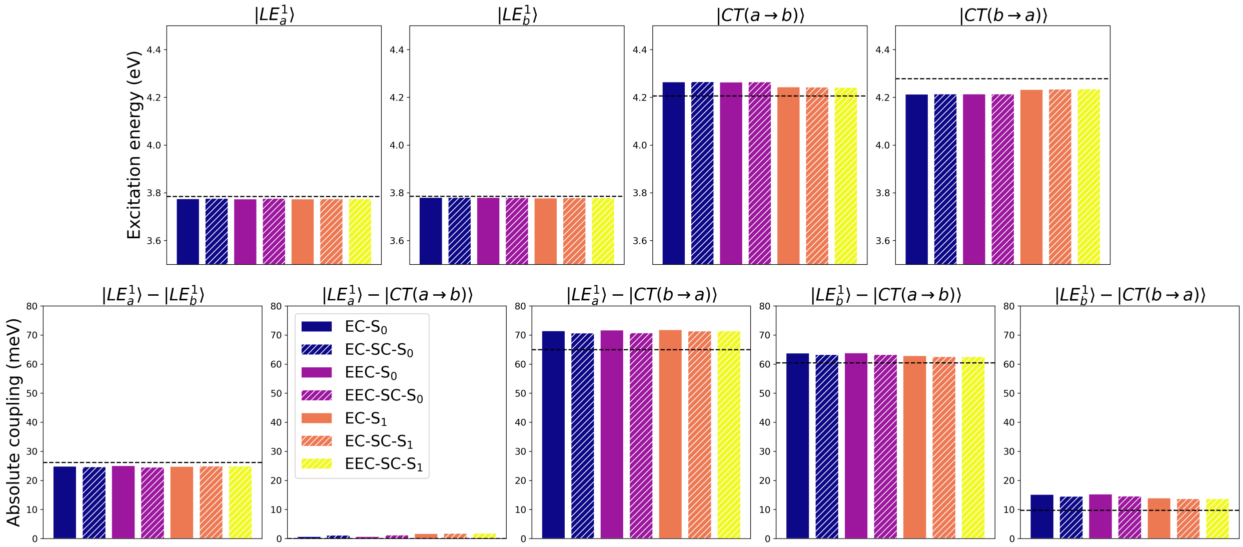

Figure S8: Excitation energies and absolute excitonic couplings of Dimer 1, computed with FrD(EE) using various electrostatic embedding schemes in `fromage` (TD-M06-2X/cc-pVDZ). Embeddings: S_0 (ground-state RESP), S_1 (excited-state RESP), SC (self-consistent), EC (embedded cluster), and EEC (Ewald embedded cluster). The dashed line indicates the value from calculation without embedding.

crystal, from which 5 dimer models (EC-S_0 in RESP charges) were extracted (Figure S9). Importantly, there are point charges between monomers in the dimer in models 2-5, enabling screening of the intermolecular interactions between monomers. Next, the RESP-based charge distribution was multiplied by factors of 2, 3, 5, and 10. For each model and scaling factor, FrD ($V_{\text{diab}}^{\text{vac}}$) and FrD(EE) ($V_{\text{diab}}^{\text{EE}}$) procedures were followed as before to obtain the $|LE_a^1\rangle - |LE_b^1\rangle$ coupling, with and without point charges. 60 singlet excited states were used for the dimer calculation (TD-M06-2X/cc-pVDZ). The effective screening, V_{eff} was straightforward to calculate,

$$V_{\text{eff}} = |V_{\text{diab}}^{\text{vac}} - V_{\text{diab}}^{\text{EE}}| \tag{S4}$$

In Figure S9, the $|LE_a^1\rangle - |LE_b^1\rangle$ couplings obtained from FrD (i.e. in vacuum) decay according to the centroid distance ($|V| \propto r^{-3}$) as outlined previously. However, in models 2 and 3, there are still large couplings between local excitations when the molecular are separated (11.7 meV, centroid distance of 7.6 Å). At very long distances, there is very small coupling (1.1 meV at 19.1 Å). In principle, dielectric screening in the solid should significantly reduce the couplings in non-nearest neighbor dimers, even if the molecules are relatively close in space (i.e. model 2). The unmodified charge distribution (1Q) has a minimal screening effect ($V_{\text{eff}} \leq 0.18$ meV) across all 5 models. However, the most polarising distribution (10Q) results in a decrease as large as 5.6 meV. The bottom panel of Figure S9 shows the percentage difference as a function of the multiplicative charge scaling, n. The 1Q model introduces a modest screening effect of approximately 0.2–5.7%, whilst 5Q results in a more substantial reduction of 10.6–17.9%. The 10Q model has a dramatic effect, reducing the excitonic coupling by 42.9–49.1%, effectively halving the size of the couplings relative to the vacuum calculation. This suggests that the number of molecules in the screen is actually less important than polarity of the distribution.

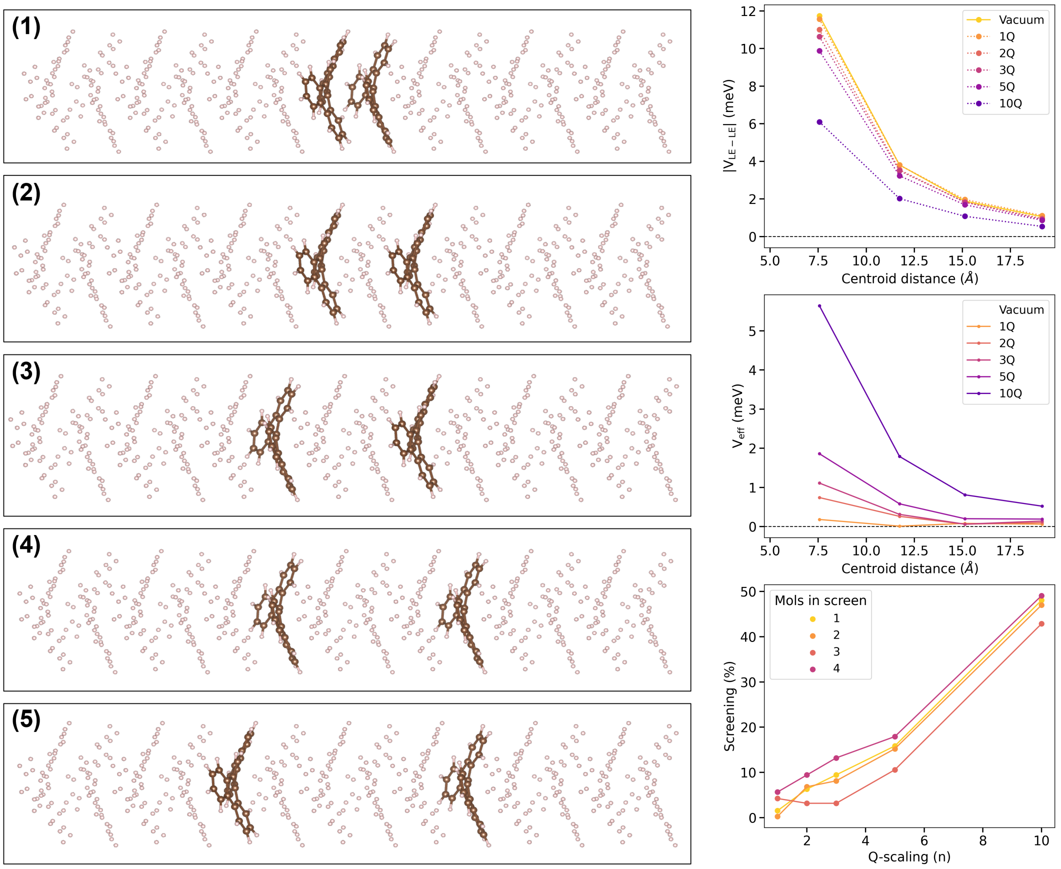

Figure S9: 1−5) Dimer models extracted for the artificial charge screening test (C: brown, H/point charges: white). Initially a π-stack of 14 DBC monomers was extracted from the relaxed crystal geometry, and point charges (white) were assigned from a population analysis on the monomer. This approach ensures a balanced treatment of charge through each model. Top) Absolute $|LE_a^1\rangle - |LE_b^1\rangle$ couplings obtained from FrD and FrD(EE) calculations. The charge distribution is scaled by a factor n, nQ where 1Q is the unmodified charge distribution. Middle) The effective screening (V_{eff}); the difference between each embedded cluster and vacuum calculation. Bottom) The difference between $|LE_a^1\rangle$ diabatic site energies on each monomer in the diabatic (FrD) basis. Calculations were performed at periodic DFT geometry (TD-M06-2X/cc-pVDZ).

S4 DBC monomer and π stacks

state	Gas Phase	THF
S_1	3.901(0.188)	3.859(0.301)
S_1-min	**3.283(0.249)** *0.30*	**3.116(0.530)** *0.40*
S_2	3.955(0.014)	3.955(0.021)
S_3	4.298(0.000)	4.299(0.000)
S_4	4.620(0.859)	4.517(1.105)
S_5	4.851(0.001)	4.867(0.000)
S_6	4.920(0.000)	4.890(0.000)

Table S5: Energies (eV) and oscillator strength (in parentheses) of the lowest energy adiabatic states of DBC monomer in the gas phases and in THF. The reorganization energy and the emission energy (in eV) are reported in italics and in bold. M062X/cc-pvdz calculations. Solvent effect inlcuded with the PCM model. [Aarabi *et al.* in preparation]

S4.1 The π stacks in the crystal (No embedding)

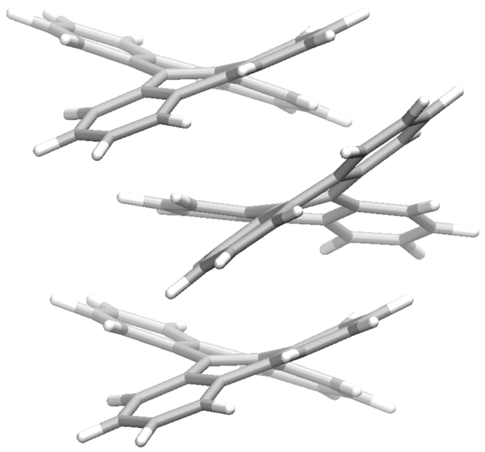

Figure S10: π stack trimer, as extracted from the DBC crystal.

S4.2 Natural transition orbitals

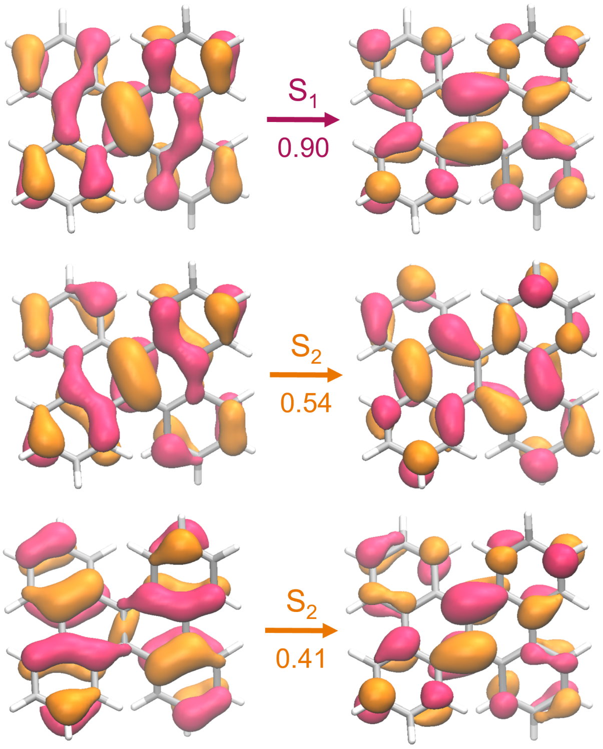

Figure S11: Visualization of the Natural Transition Orbitals associated to the two lowest energy excited states of DBC, according to M06-2X/cc-pVDZ calculations in the gas phase. Gas phase partial geometry optimizations keeping the same dihedral angles as in the crystal. The coefficients of the different Hole→Particle transitions are also shown.

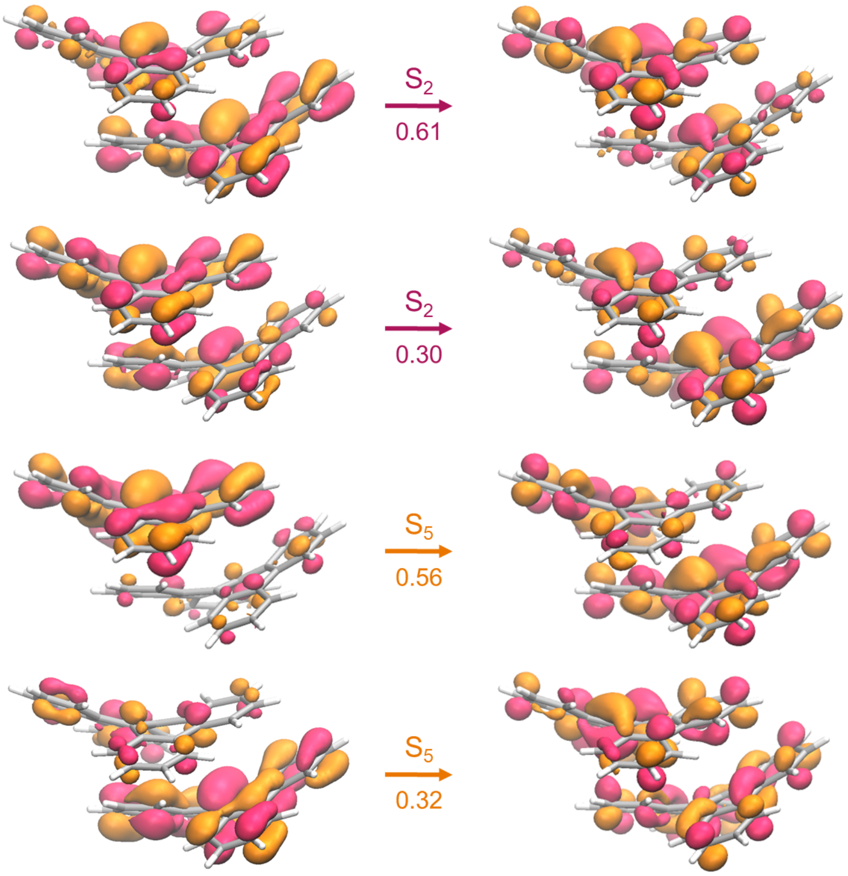

Figure S12: Visualization of the Hole→Particle Natural Transition Orbitals associated to S_2 and S_5 excited states of the DBC dimer in a 'crystal-like' arrangement (see text), according to M06-2X/cc-pVDZ partial geometry optimizations in the gas phase. The coefficients of the different Hole→Particle transitions are shown below the arrow.

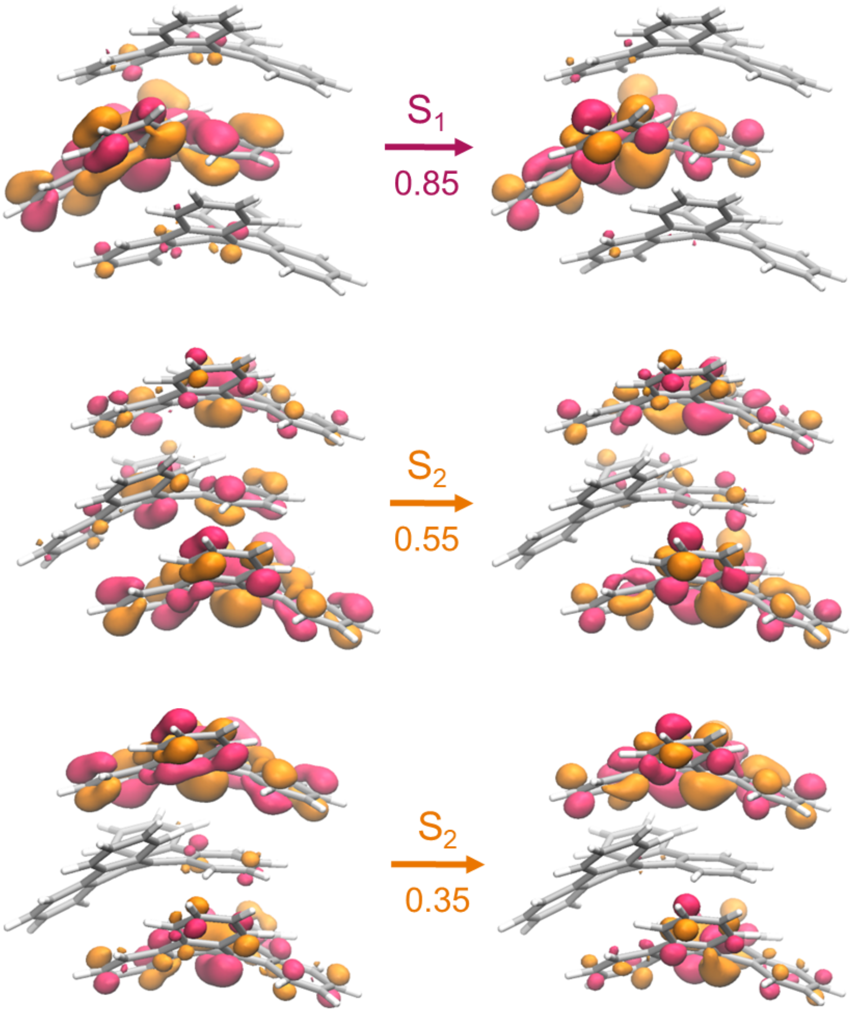

Figure S13: Visualization of the Natural Transition Orbitals associated to the two lowest energy excited states of a π stack trimer, in a 'crystal-like' arrangement (see text), according to M06-2X/cc-pVDZ partial geometry optimizations in the gas phase. The coefficients of the different Hole→Particle transitions are shown below the arrow. The NTOs associated to S_3 are similar to those shown for S_2.

S4.3 Partial geometry optimizations

We have studied a dimer and a trimer (see Figure S10) of a π stack, keeping the same stacking arrangement (see Table S6) adopted in the crystal after geometry relaxation (see Computational details). Before describing the results of this analysis, it is however, important to remember that any arbitrary partition of the crystal is prone to some artifact. For example, the two monomers in our dimer are not perfectly equivalent, as one can be considered a weakly hydrogen bond donor and another a weakly bond acceptor (Figure S10).

Table S6: Energies (eV) and oscillator strength (in parentheses) of the lowest energy adiabatic states of DBC monomer, and crystal-like π-dimer, and π-trimers. Gas-phase calculations with monomer arrangements from the relaxed crystal structure. The reorganization energy and emission energy (in eV) are reported in italics and bold, respectively.

	State	Monomer	Dimer	Trimer
	S_1	3.863 (0.188)	3.782 (0.010)	3.714 (0.010)
	S_1-min	**3.283** (0.249)*0.30**	**3.229** (0.140)*0.25*	**3.152**(0.080)*0.28*
Crystal-like	S_2	3.922 (0.009)	3.842 (0.226)	3.798 (0.005)
			**3.562**(0.279)*0.14*	**3.199** (0.130)*0.26*
	S_3	4.273 (0.002)	3.909 (0.005)	3.833 (0.257)
				**3.244** (0.129)*0.28*
	S_4	4.593 (0.880)	3.926 (0.017)	3.880 (0.007)
	S_5		4.103 (0.003)	3.905 (0.009)
	S_6		4.222 (0.010)	3.933 (0.011)
	S_7		4.290 (0.005)	4.025 (0.007)
	S_8		4.387 (0.016)	4.145 (0.000)
	S_9			4.179 (0.004)

Notes. *) This is considered to be the same as for the free monomer.

In the crystal-like dimer, the two lowest energy excited states derive by the combination of the lowest energy excited state of the monomers (see Tables S6). One of the resulting exciton is intense (S_2), whereas the other is very weak (S_1). Analogously, the two following excited states, S_3 and S_4, derives from the combination of the second lowest energy excited states of the monomer, and are also very weak. In the crystal like dimer, S_5 has a partial, yet small charge transfer (CT) character (the NTOs associated to S_2 and S_5 are reported in Figure S12). Interestingly, in the dimer examined both S_1 and S_2 are red-shifted with respect to the lowest energy excited state in the monomer.

Partial geometry optimization of S_1 in the dimer (see Table S6) leads to a minimum where the excitation is essentially localized on a single DBC monomer (see Figure S14), with a small red-shift of the emission energy with respect to the isolated monomer and a substantial decrease of the oscillator strength. S_2 partial optimization leads instead to a delocalized minimum, where the involved NTOs do not change with respect to those in the FC region (shown in Figure S12). This minimum is still on the S_2 adiabatic PES, and it is 0.18 eV less stable than the S_1 minimum, and its reorganization energy is one half of that associated to the localized minimum.

In the crystal-like trimer, the three lowest energy singlet excited states originate from the mixing of the HOMO→LUMO transitions of the three monomers. Interestingly, visualisation of the NTOs (Figure S13) reveals the lowest energy one is mainly localized on the 'central' monomer, whereas there is a much larger

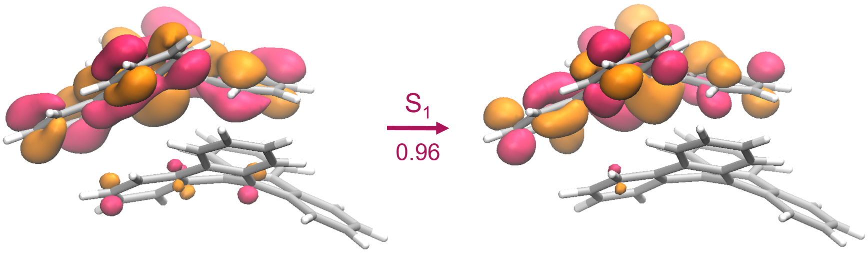

Figure S14: Visualization of the Natural Transition Orbitals associated to the S_1 minimum of a dimer in a 'crystal like' arrangement (see text) according to M06-2X/cc-pVDZ partial geometry optimizations in the gas phase. The coefficients of the main Hole→Particle transition is shown below the arrow.

contribution from the 'external' monomers in S_2 (dark) and S_3 (bright).

Partial geometry optimizations of these excited states lead to localized minima, always on the lowest energy adiabatic surface, on the central monomer (S_1) or on the two external ones (S_2 and S_3). In all the cases, a small delocalization on the stacked partners is present, as shown in Figure S15. The emission energy from the 'central' S_1 minimum is red-shifted and less intense with respect the two other localized minima, likely because it receives a small contribution from the two stacked partners. The other two minima, as might be expected, exhibit features similar to those found for the dimer.

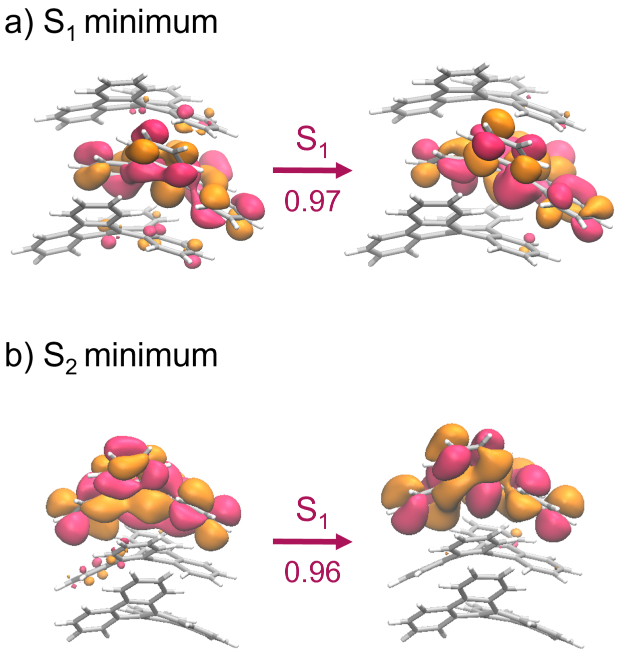

Figure S15: Visualization of the Natural Transition Orbitals associated to the S_1 (top) and S_2 (bottom) minima of a trimer in a 'crystal like' arrangement (see text), according to M06-2X/cc-pVDZ partial geometry optimizations in the gas phase. The coefficients of the main Hole→Particle transitions are shown below the arrow.

S4.4 FrD and FrD(EE) calculations on the π-stacked dimer

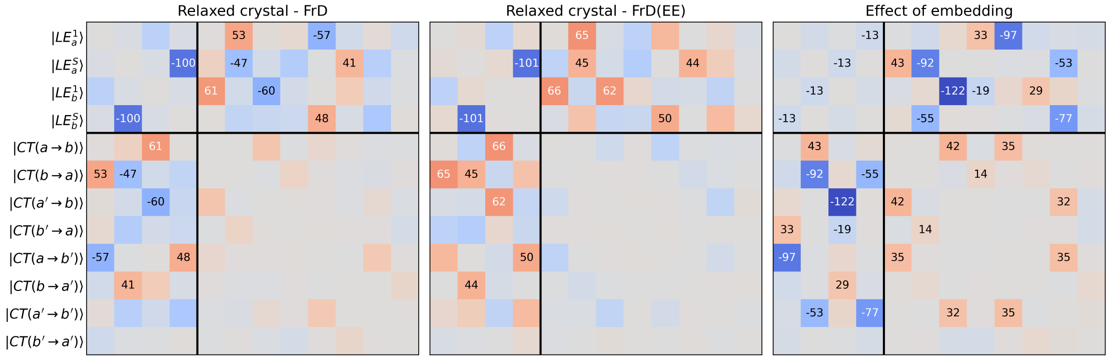

(a) 'Crystal' geometry

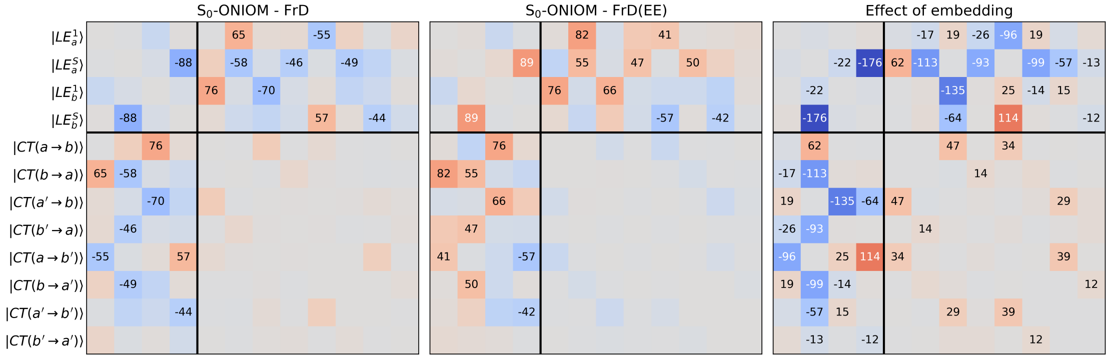

(b) S_0-ONIOM geometry

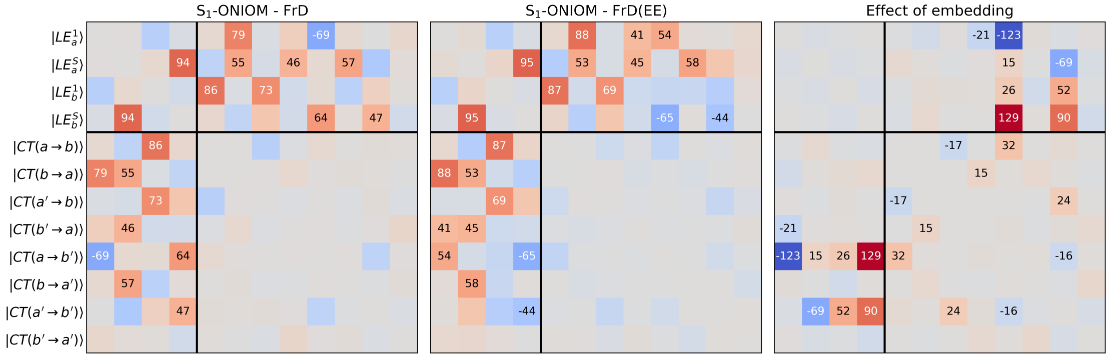

(c) S_1-ONIOM geometry

Figure S16: Excitonic couplings between diabatic states obtained from the FrD and FrD(EE) (TD-M06-2X/cc-pVDZ), and the difference matrix representing the effect of the embedding.

S4.5 Consistency of adiabatic eigenstates between FrD-LVC and FrD-LVC(EE) models

Figure S16 shows each diabatic Hamiltonian, including the sign of each coupling. The phase of the sign relates to the alignment of transition dipoles. During the screening, the phase of the sign was consistent between FrD and FrD(EE) for all of the couplings studies. However, when using a larger diabatic basis and relaxing with ONIOM, we observe a change of sign in many of the couplings. For instance, the strongest coupling ($|LE_a^S\rangle$-$|LE_b^S\rangle$) is positive at the crystal geometry and negative at the S_1 minimum, for both FrD and FrD(EE). However, at the S_0-ONIOM, embedding induces a change of sign of this coupling, and many of the others.

Interestingly, inspection of the transition dipole moments (Figure S18) obtained from the adiabatic LVC states (i.e., diagonalising the diabatic basis), shows that the TDMs are very similar across all states the S_1 minimum, however a significant change is detected along each Cartesian axis for the 'crystal' and S_0-ONIOM models. In the 'crystal' model, there are fewer changes of sign compared to the S_0-ONIOM, and although the the transition dipole moment on each axis is very different between 'crystal' and S_0-ONIOM structures, they are fairly symmetric, resulting in a the same relative alignment of dipoles (i.e. constructive or destructive) in each case. In contrast, for the S_0-ONIOM structure, the distribution along the y-axis is very similar, meaning the relative dipoles (with the x- and z-axes) mostly change in sign, along with the type of aggregation. Nevertheless, this likely has little effect on the excited states; when we take the sum-of-squares, we get very similar TDMs in each case. Similarly, we obtained very similar eigenstates (Figure S17), where the minor differences arise from the embedding. Finally, for comparison, both figures are annotated with the excitation energy and TDM from the adiabatic dimer states, which further validates the quality of this projection in the reduced (8 state) diabatic basis.

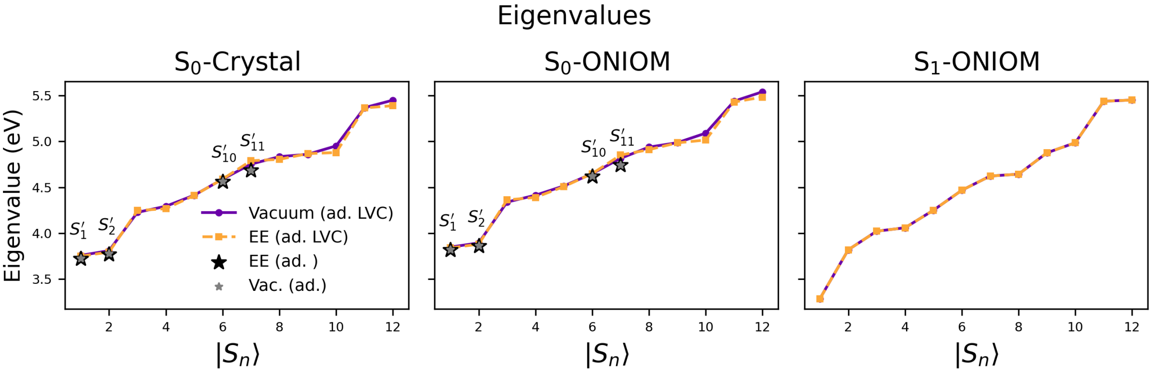

Figure S17: For each geometry of the π-dimer: Eigenvalues of the diabatic Hamiltonian (LVC adiabatic eigenstates) for each state ($|S_n\rangle$). Results are shown with and without electrostatic embedding. The true adiabatic states used for the projection (S'_n) are indicated with stars.

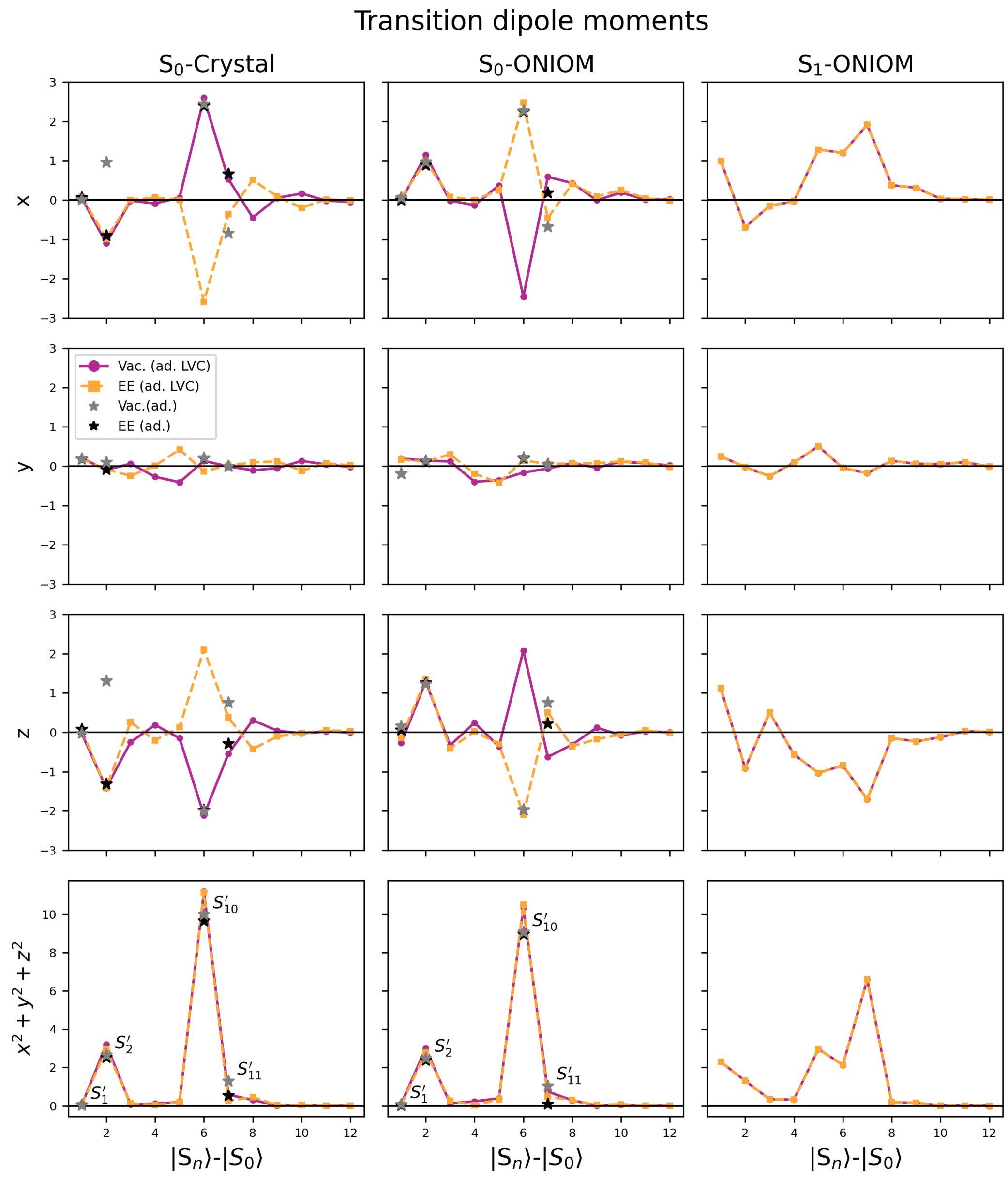

Figure S18: Transition dipole moments o along each Cartesian coordinate and the sum of their squares for each LVC adiabatic state ($|S_n\rangle$) with $|S_0\rangle$. Results are shown with and without electrostatic embedding. The true adiabatic states used for the projection (S'_n) are indicated stars.

S4.6 FrD(EE) on the π Trimer

Finally, to validate that the π dimer is a sufficiently large model, FrD(EE) was performed on the a trimer from the π stack. The trimer geometry was obtained from ONIOM relaxations as before, in Ewald charges (EEC-S_0) as before, using the Ewald charge distribution generated for the monomer. Analogous diabatic states to the dimer were defined (Figures S2 and S3), where a is DBC molecule at the centre of the trimer, and b and c are the outer molecules in the dimer (i.e. introducing $|LE_c^1\rangle$ and $|LE_c^S\rangle$ local excitations). Similarly, charge transfer states were defined for HOMO→LUMO transitions between monomers as before. For the trimer, 75 singlet states were used to converge the projection.

Moreover, these results indicate that the diabatic states of the π-trimer are to the π dimer, and that adding an additional DBC molecule does not significantly change the couplings or excitation energies. Consequently, a π dimer model is sufficient for our quantum dynamics simulations to study the interaction between monomers present in the crystal.

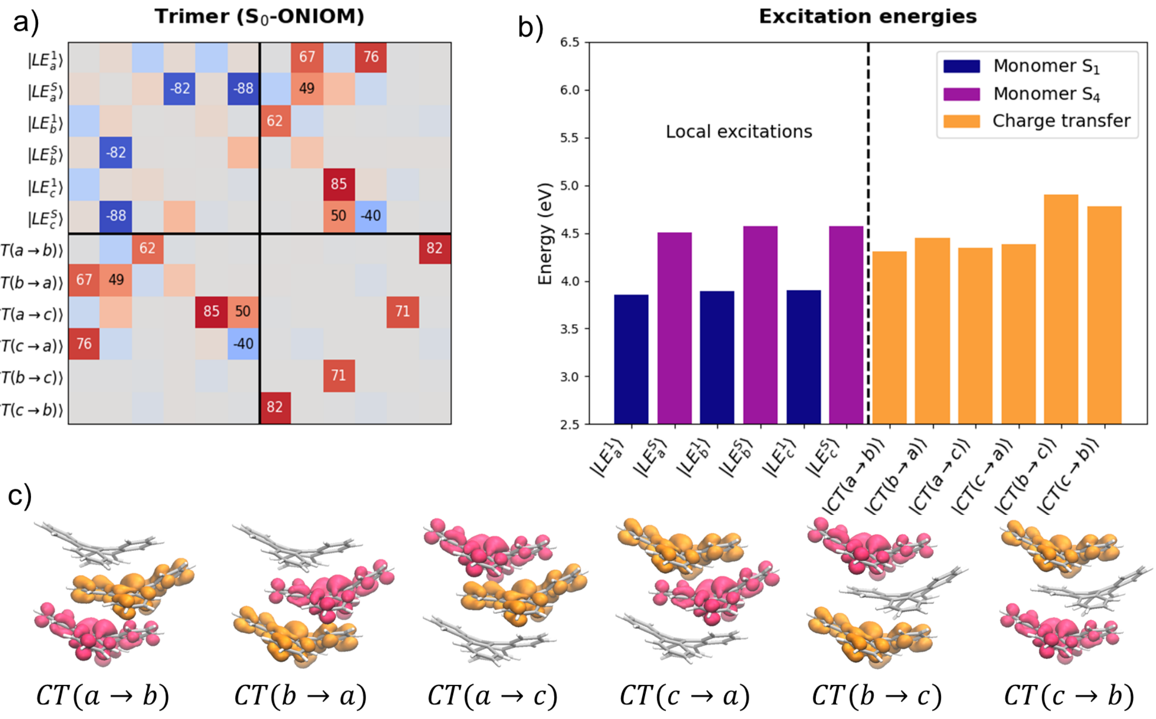

Figure S19: a) Heat map of the diabatic Hamiltonian off-diagonal elements at the S_0-ONIOM geometry of the π-trimer. The elements represent excitonic couplings (in meV) between diabatic states. b) Diabatic site energies (in eV). c) Visualization of the user-defined CT states in the diabatic basis. The HOMO (blue/green) and LUMO (yellow/orange) from the adiabatic states of each monomer, visualised on the entire trimer.

S5 FrD-LVC and quantum dynamics

S5.1 Monomer properties

Table S7: Adiabatic reference states (TD-M06-2X/cc-pVDZ) of each Ewald-embedded monomer at the S_0-ONIOM geometry. The second monomer is indicated below in parentheses.

Excited State	Energy (eV)	Osc.	Transition	Character	Weight (%)
S_1	3.90	0.180	H → L	π/π^*	87
	(3.90)	(0.170)	(H → L)	(π/π^*)	(81)
S_2	3.95	0.030	H−1 → L	$n, \pi/\pi^*$	56
			H → L+1	π/π^*	34
	(3.96)	(0.034)	(H−1 → L)	$(n, \pi/\pi^*)$	(52)
			(H → L+1)	(π/π^*)	(33)
S_3	4.29	0.010	H → L+2	π/π^*	57
			H−2 → L	π/π^*	25
	(4.29)	(0.004)	(H → L+2)	(π/π^*)	(57)
			(H−2 → L)	(π/π^*)	(26)
S_4	4.63	0.850	H−1 → L	$n, \pi/\pi^*$	52
			H → L+1	π/π^*	31
	(4.62)	(0.845)	(H−1 → L)	$(n, \pi/\pi^*)$	(53)
			(H → L+1)	(π/π^*)	(33)

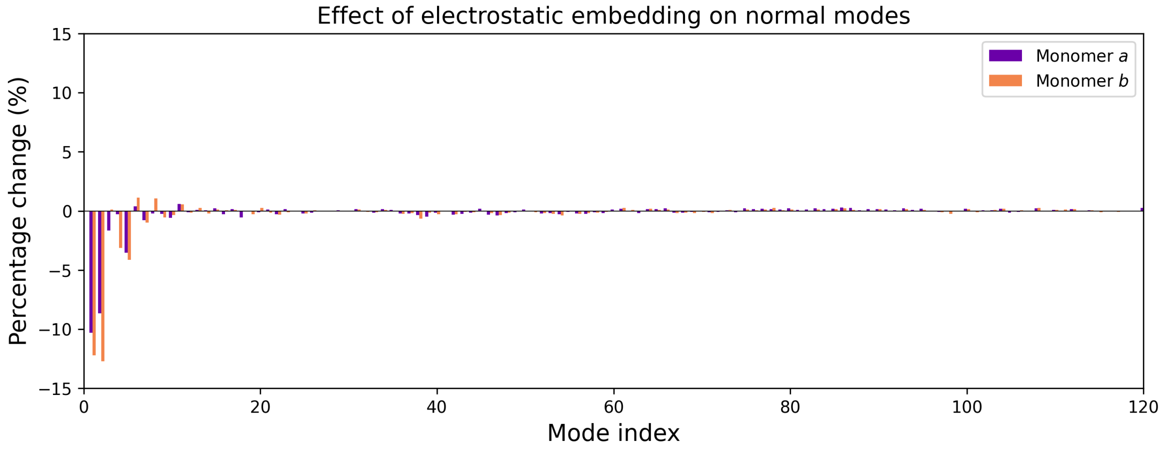

Figure S20: Difference in normal modes due to electrostatic embedding. The monomer modes were calculated at the S_0-ONIOM minimum of the dimer. The normal modes in vacuum were calculated at the FC point of the monomer (optimized in gas-phase). The percentage difference with the vacuum is calculated for both monomers. Calculations at the M06-2X/cc-pVDZ level of theory.

S5.2 FrD-LVC(EE) and quantum dynamics involving the strong ($|LE^S\rangle$) local excitations

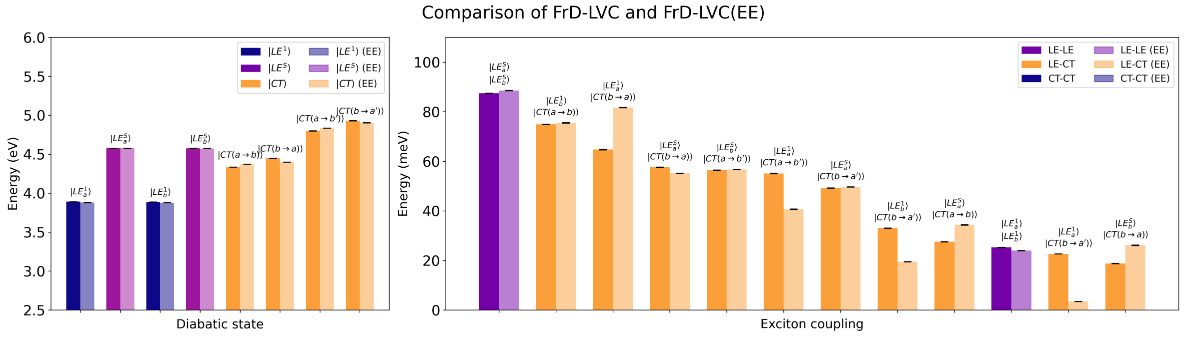

Figure S21: Effect of the electrostatic embedding on the excitation energies and absolute exciton couplings, obtained via the FrD-LVC and FrD-LVC(EE) approaches for Dimer 1. The standard deviation across all displayed geometries are shown shown as error bars. Only the 12 strongest couplings are shown. Calculations at the M06-2X/cc-pVDZ level of theory.

Table S8: The properties of the adiabatic LVC states (labeled S_1-S_8) of Dimer 1, including their energy (in eV), oscillator strength, and projection (weight) on the different diabatic states. The locally excited states are correspond to the S_1 (LE^1) and the 'strong' S_4 (LE^S) states of the monomers predicted with the FrD-LVC(EE) and FrD-LVC (in square brackets) Hamiltonian models, parameterized at M06-2X/cc-pVDZ level of theory.

State	S_1	S_2	S_3	S_4	S_5	S_6	S_7	S_8
$\|LE_a^1\rangle$	0.481	0.493	0.007	0.013	0.003	0.001	0.002	0.000
	[0.429]	[0.554]	[0.000]	[0.011]	[0.002]	[0.001]	[0.003]	[0.001]
$\|LE_a^S\rangle$	0.000	0.001	0.132	0.027	0.274	0.537	0.004	0.026
	[0.000]	[0.001]	[0.026]	[0.205]	[0.200]	[0.537]	[0.010]	[0.023]
$\|LE_b^1\rangle$	0.499	0.476	0.019	0.005	0.000	0.000	0.001	0.000
	[0.553]	[0.418]	[0.028]	[0.000]	[0.000]	[0.000]	[0.000]	[0.001]
$\|LE_b^S\rangle$	0.000	0.000	0.065	0.024	0.474	0.387	0.048	0.003
	[0.000]	[0.000]	[0.009]	[0.119]	[0.447]	[0.356]	[0.066]	[0.003]
$\|CT(a \to b)\rangle$	0.012	0.010	0.473	0.470	0.026	0.007	0.003	0.000
	[0.013]	[0.013]	[0.931]	[0.031]	[0.008]	[0.003]	[0.000]	[0.000]
$\|CT(b \to a)\rangle$	0.007	0.018	0.303	0.457	0.204	0.012	0.000	0.000
	[0.004]	[0.010]	[0.006]	[0.632]	[0.322]	[0.026]	[0.000]	[0.000]
$\|CT(a \to b')\rangle$	0.000	0.002	0.000	0.003	0.018	0.036	0.939	0.002
	[0.001]	[0.002]	[0.000]	[0.002]	[0.019]	[0.057]	[0.918]	[0.000]
$\|CT(b \to a')\rangle$	0.000	0.000	0.001	0.001	0.002	0.022	0.004	0.968
	[0.000]	[0.001]	[0.000]	[0.001]	[0.002]	[0.021]	[0.002]	[0.972]
E (in eV)	3.843	3.886	4.364	4.391	4.515	4.653	4.853	4.911
	[3.853]	[3.898]	[4.341]	[4.416]	[4.522]	[4.652]	[4.817]	[4.939]
f	0.004	0.270	0.028	0.004	0.038	1.202	0.058	0.038
	[0.007]	[0.290]	[0.012]	[0.026]	[0.046]	[1.181]	[0.089]	[0.035]

Table S9: Norm (in eV) of the linear coupling vectors for the Dimer 1, predicted with the FrD-LVC(EE) and FrD-LVC (in square brackets) Hamiltonian models, where the locally excited (LE) states are defined to correspond to the adiabatic S_1 and S_4 bright states of the monomers. Parameterization is done at the M06-2X/cc-pVDZ level of theory.

States	$\|LE_a^1\rangle$	$\|LE_a^S\rangle$	$\|LE_b^1\rangle$	$\|LE_b^S\rangle$	$\|CT(a \to b)\rangle$	$\|CT(b \to a)\rangle$	$\|CT(a \to b')\rangle$	$\|CT(b \to a')\rangle$
$\|LE_a^1\rangle$	0.290							
	[0.300]							
$\|LE_a^S\rangle$	0.207	0.219						
	[0.215]	[0.230]						
$\|LE_b^1\rangle$	0.007	0.006	0.281					
	[0.008]	[0.007]	[0.297]					
$\|LE_b^S\rangle$	0.007	0.011	0.194	0.220				
	[0.008]	[0.010]	[0.212]	[0.229]				
$\|CT(a \to b)\rangle$	0.019	0.015	0.017	0.017	0.285			
	[0.020]	[0.014]	[0.018]	[0.016]	[0.292]			
$\|CT(b \to a)\rangle$	0.019	0.015	0.020	0.017	0.007	0.275		
	[0.017]	[0.014]	[0.021]	[0.016]	[0.004]	[0.282]		
$\|CT(a \to b')\rangle$	0.020	0.014	0.007	0.013	0.146	0.004	0.277	
	[0.022]	[0.014]	[0.006]	[0.013]	[0.149]	[0.004]	[0.280]	
$\|CT(b \to a')\rangle$	0.005	0.012	0.015	0.012	0.002	0.141	0.003	0.280
	[0.006]	[0.012]	[0.018]	[0.013]	[0.002]	[0.143]	[0.003]	[0.280]

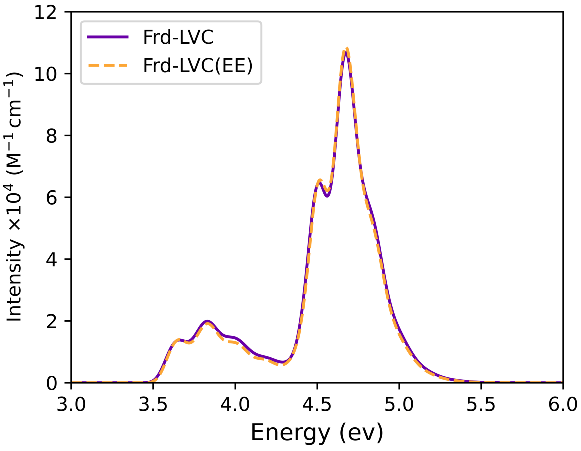

Figure S22: The nonadiabatic vibronic absorption spectrum of Dimer 1 calculated from ML-MCTDH dynamics using the FrD-LVC and FrD-LVC(EE) Hamiltonians. The stick transitions were convoluted with a Guassian broadening with HWHM=0.05 eV.

S5.3 Normal mode convergence

Several simulations have been done to assess the convergence of the dynamics with respect to the number of modes, specifically using 30, 36, 46, 70, 90, and 110 modes (Figs. S23-S26). The exclusion/inclusion of the modes, presented in Table S10, is done based on the magnitude of the inter/intra-state couplings. A symmetric distribution of equivalent normal modes across the two monomers was maintained.

Table S10: The vibrational modes included in different quantum dynamics simulations to assess convergence with respect to the number of modes.

30 modes	36 modes	46 modes	70 modes	90 modes	110 modes
17	13, 17	13, 14, 17	13, 14, 17	13, 14, 17	13, 14, 17
82, 85, 88	64	21	21	21	21
89	82, 83, 85	34	34	34	34
91, 95, 96	88, 89	60, 63, 66	41	41	41
98, 99	91, 95, 96	70, 72, 79	60, 63, 64	60, 63, 64	59
100, 101, 102	98, 99	85, 88	66	66, 69	60, 61, 62
103, 104	100, 101, 102	91, 95, 96	70, 71, 72	70, 71, 72	63, 64, 65
137	103, 104	98, 99	75, 79	73, 74, 75	66, 69
202, 205, 208	133, 137	100, 101, 102	80, 83, 85	76, 77, 79	70, 71, 72
209	184	103, 104	86, 88, 89	80, 82, 83	73, 74, 75
211, 215, 216	202, 203, 205	133, 134, 137	91, 92, 93	84, 85, 86	76, 77, 78
218, 219	208, 209	141	95, 96, 98	87, 88, 89	79
220, 221, 222	211, 215, 216	154	99	91, 92, 93	80, 82, 83
223, 224	218, 219	180, 183, 186	100, 101, 102	95, 96, 97	84, 85, 86
	220, 221, 222	190, 193, 199	103, 104, 106	98, 99	87, 88, 89
	223, 224	205, 208	119	100, 101, 102	90, 91, 92
		211, 215, 216	133, 134, 137	103, 104, 106	93, 94, 95
		218, 219	141	119	96, 97, 98
		220, 221, 222	154	120	99
		223, 224	161	133, 134, 137	100, 101, 102
			180, 183, 184	141	103, 104, 106
			186	154	107
			190, 191, 193	161	113, 118, 119
			195, 199	180, 183, 184	120
			200, 203, 205	186, 189	133, 134, 137
			206, 208, 209	190, 191, 192	141
			211, 212, 213	193, 194, 195	154
			215, 216, 218	196, 197, 199	161
			219	200, 202, 203	179
			220, 221, 222	204, 205, 206	180, 181, 182
			223, 224, 226	207, 208, 209	183, 184, 185
			239	211, 212, 213	186, 189
				215, 216, 217	190, 191, 192
				218, 219	193, 194, 195
				220, 221, 222	196, 197, 198
				223, 224, 226	199
				239	200, 202, 203
				240	204, 205, 206
					207, 208, 209
					210, 211, 212
					213, 214, 215
					216, 217, 218
					219
					220, 221, 222
					223, 224, 226
					227
					233, 238, 239
					240

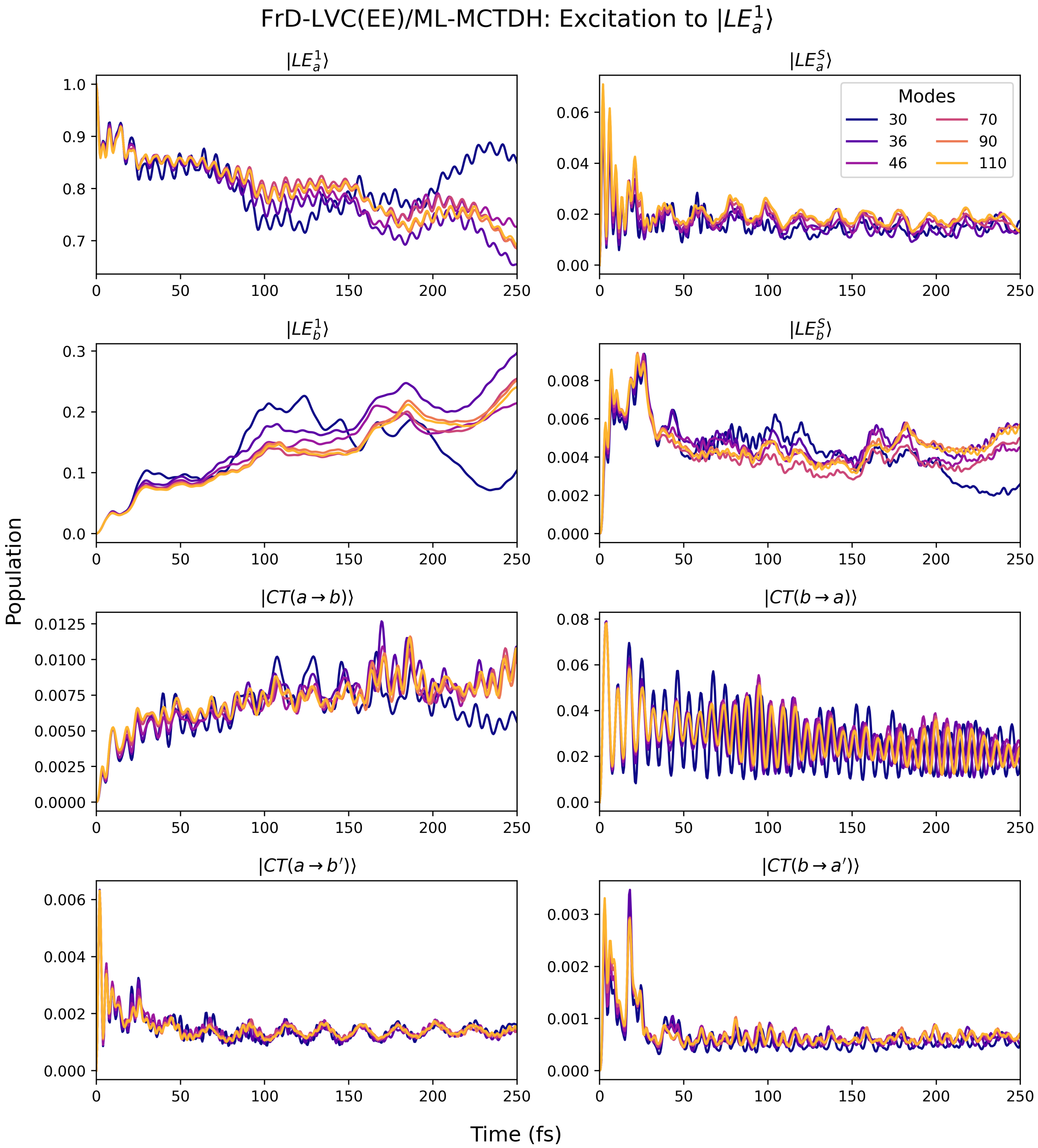

Figure S23: Population dynamics of the diabatic states of the DBC dimer, where the locally excited (LE) states are defined to correspond to the S_1 (LE^1) and S_4 (LE^S) states of the monomers, following photoexcitation from $|LE_a^1\rangle$, using different number of vibrational modes. The simulations are based on a FrD-LVC(EE) Hamiltonian comprising 8 diabatic states, parameterized at the M06-2X/cc-pVDZ level of theory with Ewald embedding (EEC-S_0).

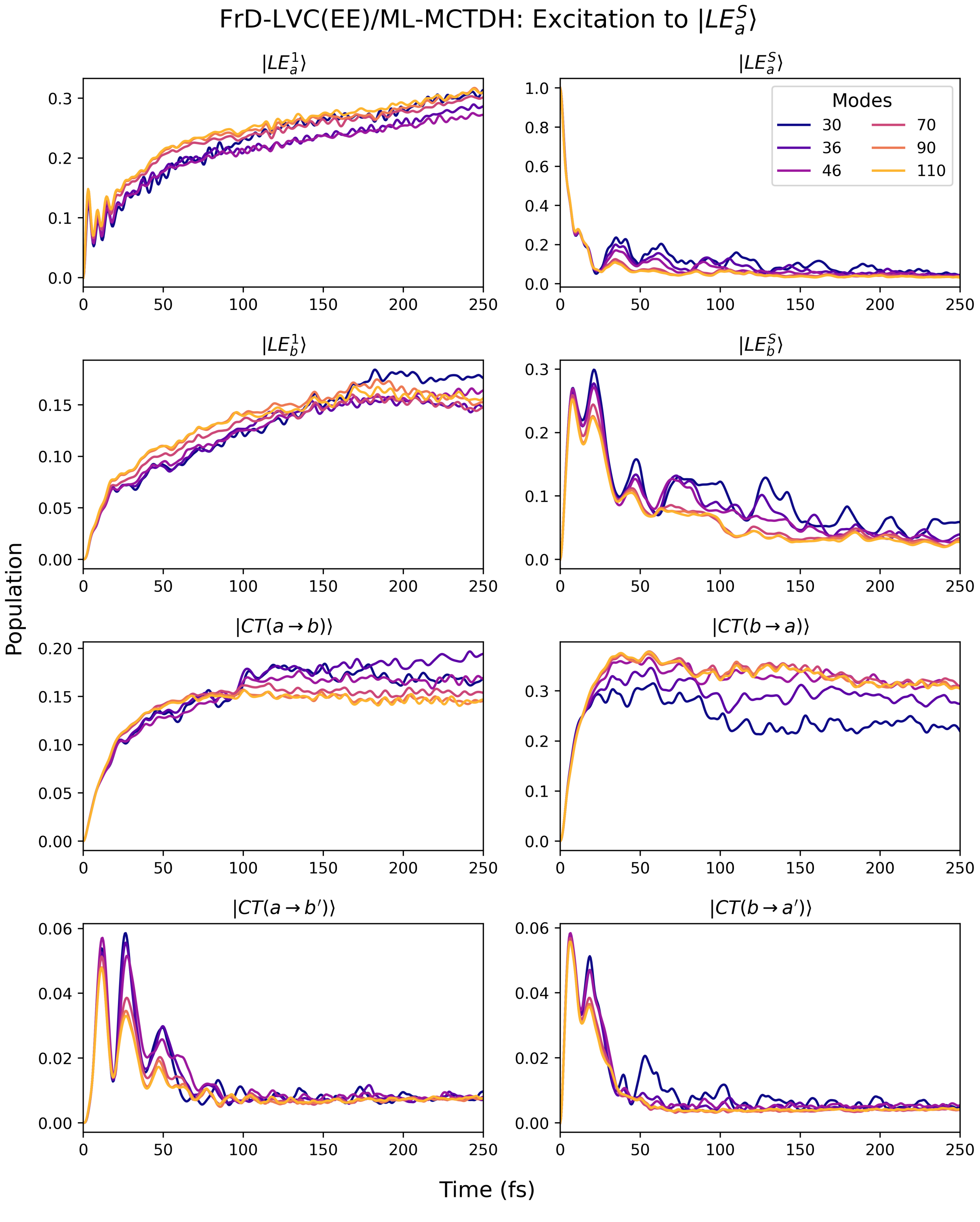

Figure S24: Population dynamics of the diabatic states of the DBC dimer, where the locally excited (LE) states are defined to correspond to the S_1 (LE^1) and S_4 (LE^S) states of the monomers, following photoexcitation from $|LE_a^S\rangle$, using different number of vibrational modes. The simulations are based on a FrD-LVC(EE) Hamiltonian comprising 8 diabatic states, parameterized at the M06-2X/cc-pVDZ level of theory with Ewald embedding (EEC-S_0).

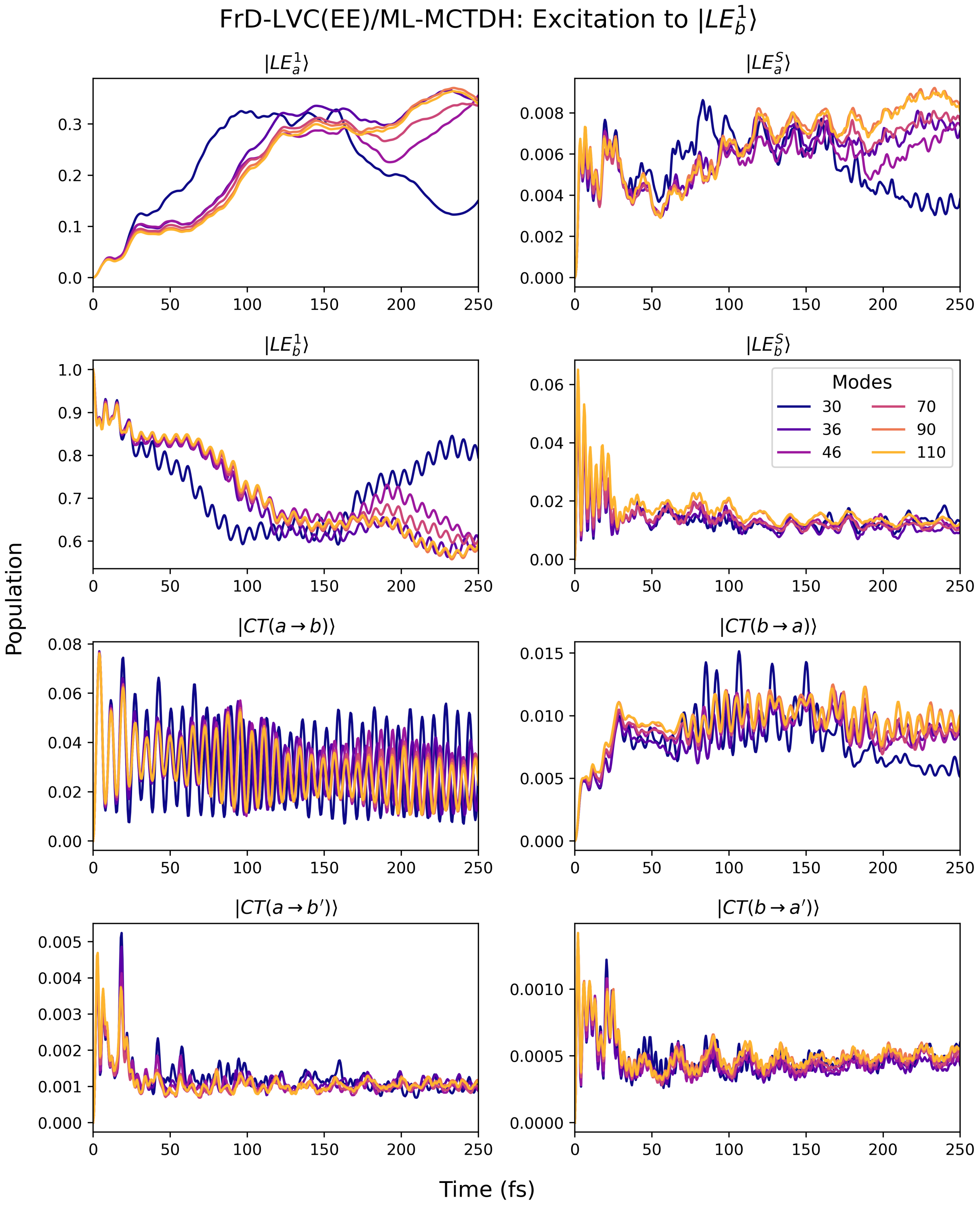

Figure S25: Population dynamics of the diabatic states of the DBC dimer, where the locally excited (LE) states are defined to correspond to the S_1 (LE^1) and S_4 (LE^S) states of the monomers, following photoexcitation from $|LE_b^1\rangle$, using different number of vibrational modes. The simulations are based on a FrD-LVC(EE) Hamiltonian comprising 8 diabatic states, parameterized at the M06-2X/cc-pVDZ level of theory with Ewald embedding (EEC-S_0).

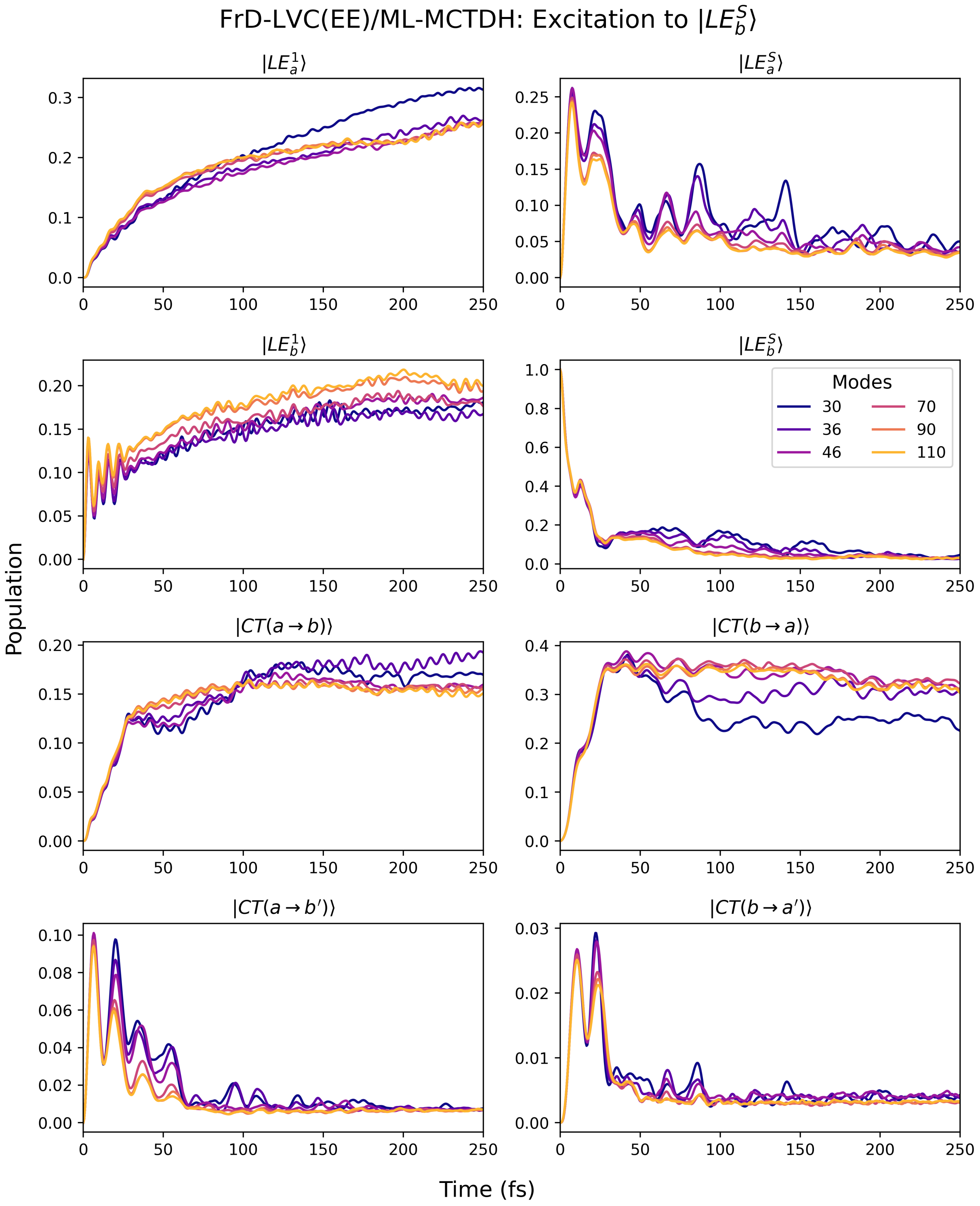

Figure S26: Population dynamics of the diabatic states of the DBC dimer, where the locally excited (LE) states are defined to correspond to the S_1 (LE^1) and S_4 (LE^S) states of the monomers, following photoexcitation from $|LE_b^S\rangle$, using different number of vibrational modes. The simulations are based on a FrD-LVC(EE) Hamiltonian comprising 8 diabatic states, parameterized at the M06-2X/cc-pVDZ level of theory with Ewald embedding (EEC-S_0).

S5.4 Photoexciting adiabatic states

Figure S27 reports the time-evolving populations of the delocalized states corresponding to the adiabatic states at the FC position (S_1-S_8), following initial excitation to S_2 (left panels) and S_6 (right panels). The analysis of the adiabatic LVC states reported in Table S8 shows that S_2 contains a strong mixing of LE states, with dominant contributions from $|LE_a^1\rangle$ and $|LE_b^1\rangle$, whereas S_6 is mainly composed of $|LE_a^S\rangle$ and $|LE_b^S\rangle$ with additional contributions from CT states, indicating a partial LE-CT mixed character. Analysis of the time-evolving wavepacket in terms of localized LE and CT diabatic populations shows that, when starting from the less bright S_2 delocalized exciton, over 50% of the population decays to S_1 within 5 fs, followed by modest oscillations arising from population exchange between the two states up to 250 fs, with almost no contribution from the CT states. However, when starting from the most intense S_6 state, more than 50% of the total population decays into CT states within 25 fs, resulting eventually in a total population of >0.5 distributed over the quasi-dark states (S_3, S_4, S_7, and S_8), all of which involve significant CT character. The bottom panel of Figure S27, where the states exhibit pure character, confirms a remarkable population of the CT states, reaching ~50% within 25 fs.

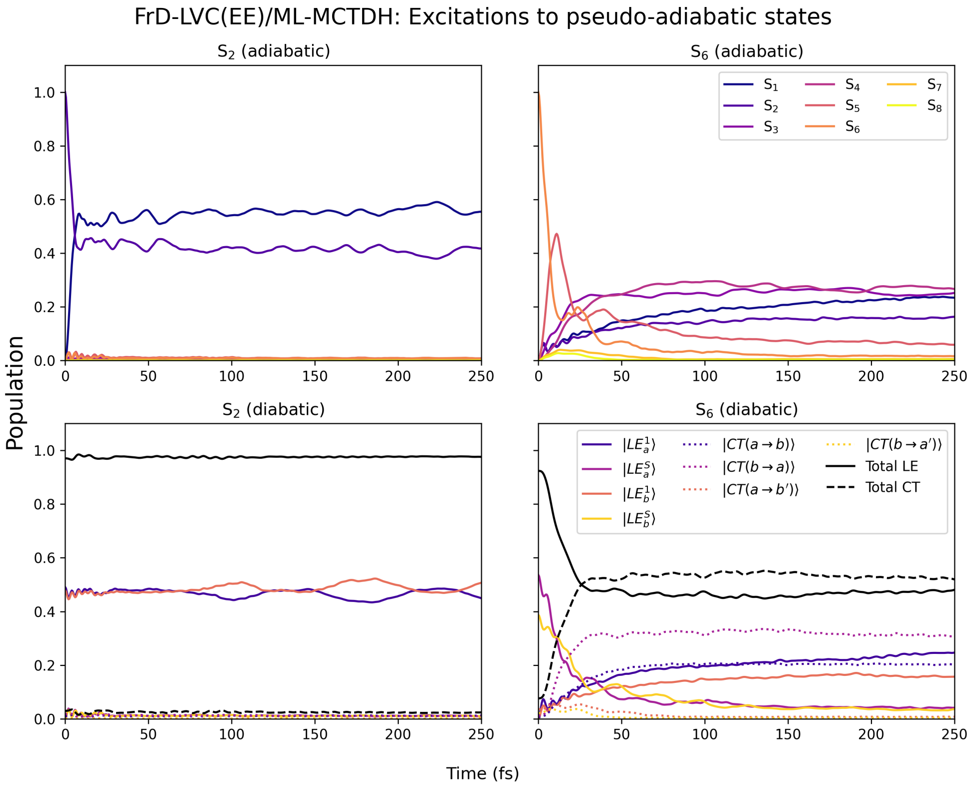

Figure S27: Population dynamics following excitation to the bright pseudo-adiabatic states (S_2 and S_4 of Dimer 1). The populations are shown both in the LVC adiabatic (top) and diabatic (bottom) representations. The LVC adiabatic states are defined in Table S8. For the diabatic representation, the total populations for local excitation and charge transfer states is shown.

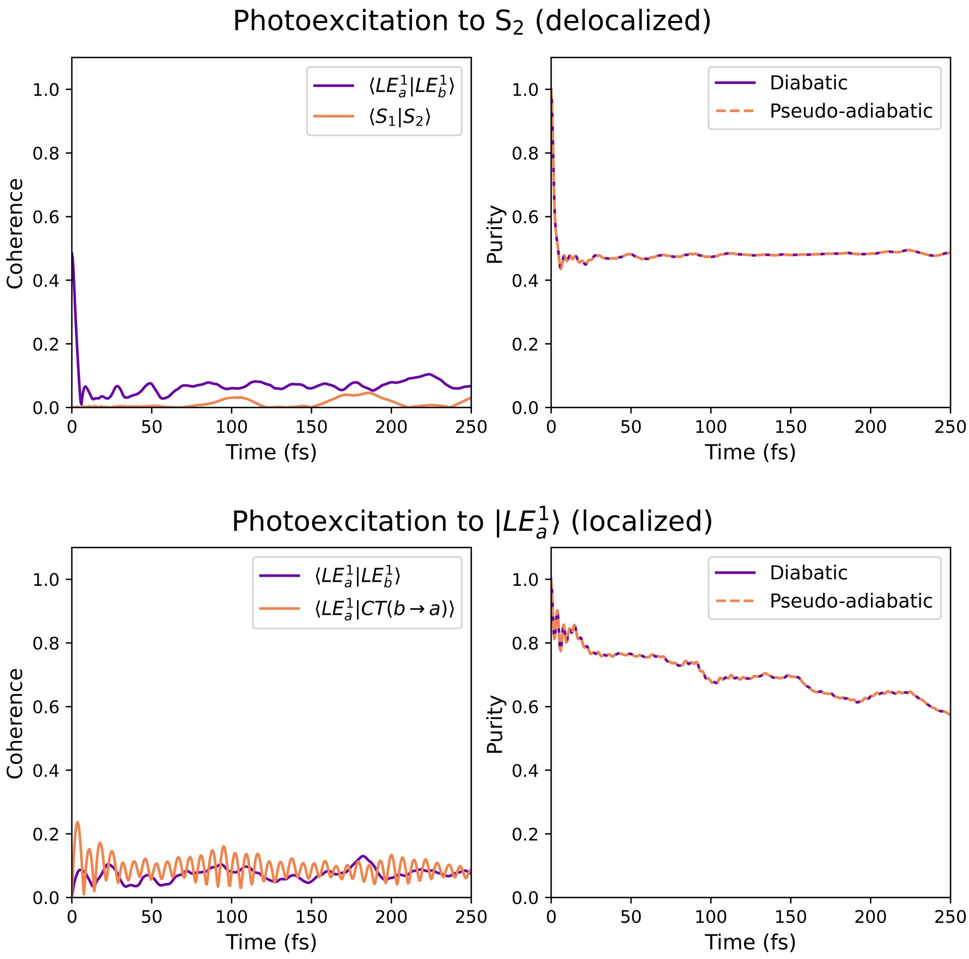

Figure S28: Coherence and purity of the diabatic and pseudo-adiabatic states following photoexcitation to S_2 and $|LE_1^a\rangle$.

S5.5 FrD-LVC(EE) and quantum dynamics involving the weak ($|LE^W\rangle$) local excitations

Table S11: LVC Hamiltonian in the diabatic representation for the DBC stacked dimer, where the locally excited states correspond to the S_1 (LE^1) and S_2 (LE^W, where the superscript 'W' denotes weak) adiabatic states of the monomers. Parameterized with the FrD-LVC(EE) Hamiltonian model at M06-2X/cc-pVDZ level of theory, including electrostatic embedding. The matrix is symmetric and only half is reported. Terms in the diagonal represent the vertical energies, $E_{ii}^d(0)$, while the off diagonal terms are the constant coupling, $E_{ij}^d(0)$. All terms are in eV.

State	$\|LE_a^1\rangle$	$\|LE_a^W\rangle$	$\|LE_b^1\rangle$	$\|LE_b^W\rangle$	$\|CT(a\to b)\rangle$	$\|CT(b\to a)\rangle$	$\|CT(a\to b')\rangle$	$\|CT(b\to a')\rangle$
$\|LE_a^1\rangle$	3.879							
$\|LE_a^W\rangle$	0.001	3.942						
$\|LE_b^1\rangle$	-0.024	-0.002	3.877					
$\|LE_b^W\rangle$	-0.009	-0.002	-0.003	3.945				
$\|CT(a\to b)\rangle$	0.006	-0.020	0.076	0.033	4.372			
$\|CT(b\to a)\rangle$	0.082	-0.029	-0.012	0.027	0.001	4.399		
$\|CT(a\to b')\rangle$	0.041	0.013	-0.019	0.065	-0.025	-0.002	4.835	
$\|CT(b\to a')\rangle$	0.003	0.066	-0.019	0.004	-0.001	-0.011	-0.000	4.903

Table S12: Energies (in eV) of Reorganization (ReE), Franck-Condon (FC) and minimum (Min) of the eight diabatic states for DBC dimer, where the locally excited (LE) states are defined to correspond to the S_1 (LE^1) and S_2 (LE^W) states of the monomers calculated with the contributions of 90 coordinates. FrD-LVC(EE) Hamiltonian model including 8 diabatic states parameterized at M06-2X/cc-pVDZ level of theory, including electrostatic embedding.

Energies	$\|LE_a^1\rangle$	$\|LE_a^W\rangle$	$\|LE_b^1\rangle$	$\|LE_b^W\rangle$	$\|CT(a \to b)\rangle$	$\|CT(b \to a)\rangle$	$\|CT(a \to b')\rangle$	$\|CT(b \to a')\rangle$
E^{FC}	3.879	3.942	3.877	3.945	4.372	4.399	4.835	4.903
E^{ReE}	0.269	0.190	0.259	0.190	0.243	0.230	0.212	0.222
E^{Min}	3.610	3.752	3.618	3.755	4.129	4.169	4.623	4.680
f	0.145	0.025	0.137	0.027	0.000	0.001	0.000	0.000

Table S13: The properties of the adiabatic LVC states (labeled S_1-S_8), including their energy (in eV), oscillator strength and their projection (weight) on the different diabatic states, for the DBC dimer, where the locally excited states correspond to the S_1 (LE^1) and S_2 (LE^W) states of the monomers predicted with the FrD-LVC(EE) model parameterized at M06-2X/cc-pVDZ level of theory, including electrostatic embedding.

State	S_1	S_2	S_3	S_4	S_5	S_6	S_7	S_8
$\|LE_a^1\rangle$	0.501	0.427	0.000	0.048	0.000	0.02	0.002	0.000
$\|LE_a^W\rangle$	0.002	0.002	0.933	0.053	0.001	0.004	0.000	0.005
$\|LE_b^1\rangle$	0.449	0.528	0.000	0.001	0.021	0.000	0.001	0.000
$\|LE_b^W\rangle$	0.024	0.015	0.059	0.888	0.006	0.003	0.005	0.000
$\|CT(a \to b)\rangle$	0.013	0.009	0.001	0.006	0.962	0.007	0.003	0.000
$\|CT(b \to a)\rangle$	0.010	0.017	0.002	0.001	0.007	0.963	0.000	0.001
$\|CT(a \to b')\rangle$	0.001	0.002	0.001	0.003	0.003	0.000	0.989	0.001
$\|CT(b \to a')\rangle$	0.000	0.000	0.004	0.000	0.000	0.001	0.001	0.994
E (in eV)	3.841	3.885	3.935	3.940	4.385	4.415	4.843	4.908
f	0.004	0.247	0.015	0.061	0.006	0.002	0.001	0.001

Table S14: Norm (in eV) of the linear coupling vectors for the Dimer 1, predicted with the FrD-LVC(EE) Hamiltonian model, where the locally excited states correspond to the S_1 (LE^1) and S_2 (LE^W), where the superscript 'W' denotes weak) adiabatic states of the monomers. Parameterization is done at M06-2X/cc-pVDZ level of theory, including electrostatic embedding.

State	$\|LE_a^1\rangle$	$\|LE_a^W\rangle$	$\|LE_b^1\rangle$	$\|LE_b^W\rangle$	$\|CT(a \to b)\rangle$	$\|CT(b \to a)\rangle$	$\|CT(a \to b')\rangle$	$\|CT(b \to a')\rangle$
$\|LE_a^1\rangle$	0.290							
$\|LE_a^W\rangle$	0.061	0.250						
$\|LE_b^1\rangle$	0.007	0.003	0.281					
$\|LE_b^W\rangle$	0.003	0.004	0.075	0.249				
$\|CT(a \to b)\rangle$	0.019	0.014	0.017	0.015	0.285			
$\|CT(b \to a)\rangle$	0.019	0.012	0.020	0.019	0.007	0.275		
$\|CT(a \to b')\rangle$	0.020	0.015	0.007	0.015	0.146	0.004	0.277	
$\|CT(b \to a')\rangle$	0.005	0.015	0.015	0.015	0.002	0.141	0.003	0.280

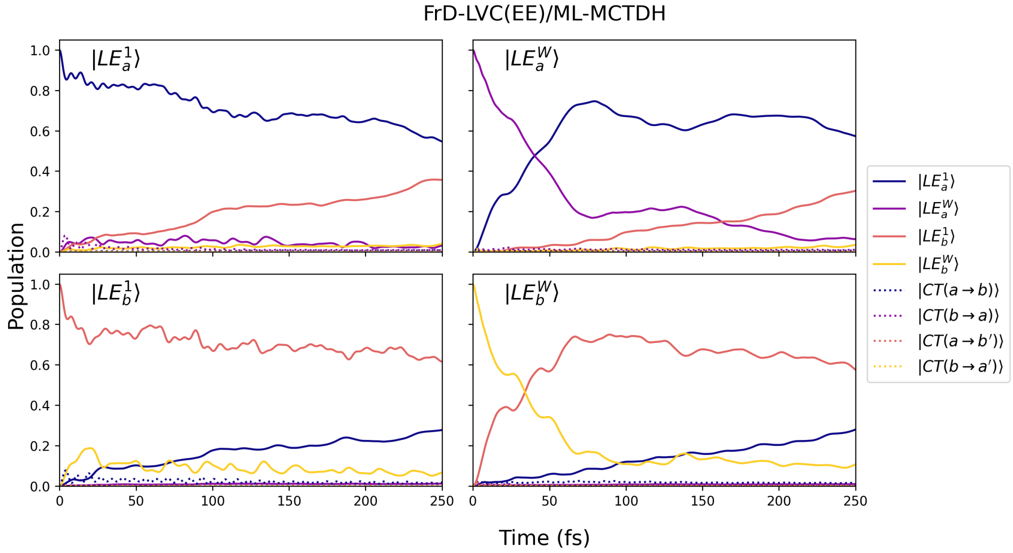

Figure S29: Population dynamics of the diabatic states of the DBC dimer, where the locally excited (LE) states are defined to correspond to the S_1 ($|LE^1\rangle$) and S_2 ($|LE^W\rangle$) states of the monomers, following photoexcitation from different initial diabatic states. The simulations are based on a FrD-LVC(EE) Hamiltonian comprising 8 diabatic states, parameterized at the M06-2X/cc-pVDZ level of theory with Ewald embedding (EEC-S_0). ML-MCTDH dynamics, including 90 effective coordinates.

References

[1] S. Giannini, W.-T. Peng, L. Cupellini, D. Padula, A. Carof and J. Blumberger, Exciton transport in molecular organic semiconductors boosted by transient quantum delocalization, *Nature Communications*, 2022, **13**, 2755.

[2] M. E. Madjet, A. Abdurahman and T. Renger, Intermolecular Coulomb Couplings from Ab Initio Electrostatic Potentials: Application to Optical Transitions of Strongly Coupled Pigments in Photosynthetic Antennae and Reaction Centers, *The Journal of Physical Chemistry B*, 2006, **110**, 17268–17281.

[3] C.-P. Hsu, G. R. Fleming, M. Head-Gordon and T. Head-Gordon, Excitation energy transfer in condensed media, *The Journal of Chemical Physics*, 2001, **114**, 3065–3072.

[4] M. F. Iozzi, B. Mennucci, J. Tomasi and R. Cammi, Excitation energy transfer (EET) between molecules in condensed matter: A novel application of the polarizable continuum model (PCM), *The Journal of Chemical Physics*, 2004, **120**, 7029–7040.

[5] G. D. Scholes, Long-Range Resonance Energy Transfer in Molecular Systems, *Annual Review of Physical Chemistry*, 2003, **54**, 57–87.

[6] B. P. Krueger, G. D. Scholes and G. R. Fleming, Calculation of Couplings and Energy-Transfer Pathways between the Pigments of LH2 by the ab Initio Transition Density Cube Method, *The Journal of Physical Chemistry B*, 1998, **102**, 5378–5386.

[7] M. J. Frisch, G. W. Trucks, H. B. Schlegel, G. E. Scuseria, M. A. Robb, J. R. Cheeseman, G. Scalmani, V. Barone, G. A. Petersson, H. Nakatsuji, X. Li, M. Caricato, A. V. Marenich, J. Bloino, B. G. Janesko, R. Gomperts, B. Mennucci, H. P. Hratchian, J. V. Ortiz, A. F. Izmaylov, J. L. Sonnenberg, D. Williams-Young, F. Ding, F. Lipparini, F. Egidi, J. Goings, B. Peng, A. Petrone, T. Henderson, D. Ranasinghe, V. G. Zakrzewski, J. Gao, N. Rega, G. Zheng, W. Liang, M. Hada, M. Ehara, K. Toyota, R. Fukuda, J. Hasegawa, M. Ishida, T. Nakajima, Y. Honda, O. Kitao, H. Nakai, T. Vreven, K. Throssell, J. A. Montgomery, Jr., J. E. Peralta, F. Ogliaro, M. J. Bearpark, J. J. Heyd, E. N. Brothers, K. N. Kudin, V. N. Staroverov, T. A. Keith, R. Kobayashi, J. Normand, K. Raghavachari, A. P. Rendell, J. C. Burant, S. S. Iyengar, J. Tomasi, M. Cossi, J. M. Millam, M. Klene, C. Adamo, R. Cammi, J. W. Ochterski, R. L. Martin, K. Morokuma, O. Farkas, J. B. Foresman and D. J. Fox, *Gaussian16, Revision A.03*, 2016.

[8] M. Yaghoubi Jouybari, Y. Liu, R. Improta and F. Santoro, Ultrafast Dynamics of the Two Lowest Bright Excited States of Cytosine and 1-Methylcytosine: A Quantum Dynamical Study, *Journal of Chemical Theory and Computation*, 2020, **16**, 5792–5808.

[9] J. A. Green, H. Asha, F. Santoro and R. Improta, Excitonic Model for Strongly Coupled Multichromophoric Systems: The Electronic Circular Dichroism Spectra of Guanine Quadruplexes as Test Cases, *Journal of Chemical Theory and Computation*, 2021, **17**, 405–415.

[10] J. A. Green, M. Yaghoubi Jouybari, H. Asha, F. Santoro and R. Improta, Fragment Diabatization Linear Vibronic Coupling Model for Quantum Dynamics of Multichromophoric Systems: Population of the Charge-Transfer State in the Photoexcited Guanine–Cytosine Pair, *Journal of Chemical Theory and Computation*, 2021, **17**, 4660–4674.

[11] A. Sidat, M. Ingham, M. Rivera, A. J. Misquitta and R. Crespo-Otero, Performance of point charge embedding schemes for excited states in molecular organic crystals, *The Journal of Chemical Physics*, 2023, **159**, 244108.
